# Genetic factors define CPO and CLO subtypes of nonsyndromicorofacial cleft

**DOI:** 10.1371/journal.pgen.1008357

**Published:** 2019-10-14

**Authors:** Lulin Huang, Zhonglin Jia, Yi Shi, Qin Du, Jiayu Shi, Ziyan Wang, Yandong Mou, Qingwei Wang, Bihe Zhang, Qing Wang, Shi Ma, He Lin, Shijun Duan, Bin Yin, Yansong Lin, Yiru Wang, Dan Jiang, Fang Hao, Lin Zhang, Haixin Wang, Suyuan Jiang, Huijuan Xu, Chengwei Yang, Chenghao Li, Jingtao Li, Bing Shi, Zhenglin Yang

**Affiliations:** 1 The Sichuan Provincial Key Laboratory for Human Disease Gene Study, Department of Clinical Laboratory, Sichuan Provincial People’s Hospital, School of Medicine, University of Electronic Science and Technology of China, Chengdu, China; 2 State Key Laboratory of Oral Diseases and National Clinical Research Center for Oral Diseases and Department of cleft lip and palate, West China Hospital of Stomatology, Sichuan University, Chengdu, China; 3 Institute of Chengdu Biology, and Sichuan Translational Medicine Hospital, Chinese Academy of Sciences, Chengdu, China; 4 Department of Stomatology, Sichuan Provincial People’s Hospital, University of Electronic Science and Technology of China, Chengdu, China; 5 Division of Growth and Development and Section of Orthodontics, School of Dentistry, University of California, Los Angeles, United States of America; 6 Department of basic medicine, School of Medicine, University of Electronic Science and Technology of China, Chengdu, China; Stanford University School of Medicine, UNITED STATES

## Abstract

Nonsyndromic orofacial cleft (NSOFC) is a severe birth defect that occurs early in embryonic development and includes the subtypes cleft palate only (CPO), cleft lip only (CLO) and cleft lip with cleft palate (CLP). Given a lack of specific genetic factor analysis for CPO and CLO, the present study aimed to dissect the landscape of genetic factors underlying the pathogenesis of these two subtypes using 6,986 cases and 10,165 controls. By combining a genome-wide association study (GWAS) for specific subtypes of CPO and CLO, as well as functional gene network and ontology pathway analysis, we identified 18 genes/loci that surpassed genome-wide significance (*P* < 5 × 10^−8^) responsible for NSOFC, including nine for CPO, seven for CLO, two for both conditions and four that contribute to the CLP subtype. Among these 18 genes/loci, 14 are novel and identified in this study and 12 contain developmental transcription factors (TFs), suggesting that TFs are the key factors for the pathogenesis of NSOFC subtypes. Interestingly, we observed an opposite effect of the genetic variants in the *IRF6* gene for CPO and CLO. Moreover, the gene expression dosage effect of *IRF6* with two different alleles at the same single-nucleotide polymorphism (SNP) plays important roles in driving CPO or CLO. In addition, *PAX9* is a key TF for CPO. Our findings define subtypes of NSOFC using genetic factors and their functional ontologies and provide a clue to improve their diagnosis and treatment in the future.

## Author summary

Although GWAS have discovered 43 genes/loci associated with NSOFC, most previous studies used mixed samples of CL/P subtypes rather than CPO or CLO separately. Our findings define the CPO and CLO subtypes using genetic factors and their functional ontologies based on CPO and CLO GWAS data. In this study, we identified 18 genes/loci that contribute to CPO, CLO or CLP. Fourteen of them are novel and identified, and 12 contain developmental transcription factors (TFs), suggesting that TFs are the key factors for the pathogenesis of NSOFC subtypes. We observed an opposite effect in the strongest associated locus *IRF6* for CPO and CLO; this information was omitted by previous CL/P GWAS. Furthermore, we reveal that the gene expression dosage of *IRF6* plays important roles in driving CPO or CLO. In addition, we found that *PAX9* is a strong genetic factor for CPO. These results suggest that transcription factors are the key genetic reason for the pathogenesis of NSOFC subtypes.

## Introduction

Cleft lip and cleft palate are orofacial disruptions of the normal facial structure that can cause problems with feeding, speaking, hearing and social integration among affected individuals [1, 2]. Estimates have suggested that orofacial clefts occur in approximately 1 in 700 live births worldwide [2–4]. The majority of orofacial clefts lack additional defects in other tissues and are categorized as nonsyndromic cleft lip with or without cleft palate (CL/P) [5], which accounts for 70% of all orofacial clefts cases. CL/P cases include cleft palate only (CPO), cleft lip only (CLO) and cleft lip with cleft palate (CLP) [6]. Asian and Native American ancestry populations generally exhibit the highest birth prevalence rates for nonsyndromic orofacial cleft (NSOFC), whereas European ancestry populations have intermediate prevalence rates, and African ancestry populations have the lowest prevalence rates [7]. The overall prevalence of NSOFC in China is 1.67 per 1,000 newborns, with rates for CPO (2.7), CLO (5.6) and CLP (8.2 per 10,000 newborns) [8].

Both genetic factors and environmental risk factors contribute to the pathogenesis of NSOFC [9]. However, it has been difficult to identify specific etiologic factors for this disorder because the defects arise during early embryological development and because recurrence is both fairly common and unpredictable [1]. Moreover, because cleft lip and cleft palate are highly genetically heterogeneous [10], it is crucial to understand the genetic contributions of facial development in order to improve the clinical care of affected individuals. Genome-wide association studies (GWAS) have led to the discovery of at least 43 genes/loci associated with NSOFC [11–20], with genetic variants in the region of the *IRF6* gene showing the strongest association with nonsyndromic CL/P among different populations [11, 16, 21–23]. Given that the lip and primary palate have distinct developmental origins from the secondary palate, and that CPO, CLO and CLP have different phenotypes, it seems reasonable to hypothesize that these disorders might also harbor different genetic etiologies. However, most previous genetic studies of NSOFC used mixed samples of different subtypes (CL/P) rather than analyzing CPO, CLO or CLP separately. Only recently, a CLP GWAS identified 14 novel loci and suggested that the CPO, CLO and CLP subtypes harbor different genetic etiologies [24]. Therefore, the genetic association signals for specific CPO or CLO subtypes may have been missed in previous studies because the true signals for the specific subtypes could have been diluted by other subtypes.

On the other hand, the typical GWAS method has limited power to identify the associated genes with disease. For example, some genes that could be genuinely associated with disease status might not reach a stringent genome-wide significance threshold via typical GWAS [25]. The low signal-to-noise ratio, inherent in the majority of large datasets, presents a major difficulty in the analysis of complex biological systems [26]. Therefore, we combined the typical GWAS method with the gene network and ontology analysis methods to explore the genetic contributions to each NSOFC subtype.

## Results

### Typical GWAS and replications identified nine novel loci responsible for NSOFC

To identify the NSOFC susceptibility genes/loci that are specific to CPO or CLO, we genotyped 935 unrelated CPO patients, 948 unrelated CLO patients and 5,050 unrelated control individuals of Southern Han Chinese ancestry using the Illumina HumanOmniZhongHua-8 BeadChip [27], which has 900,015 single-nucleotide polymorphisms (SNPs) in our discovery stage. Sample collection for the cohorts is shown in Table 1 and S1 Fig. Additional details regarding patient recruitment and disease definition are provided in the Methods section. Full details of the experimental workflow are provided in S2 Fig. The total genotyping rate of the dataset is 0.9949.

**Table 1:** Sample collection for the cohorts.

Cohort	CPO	CLO	CLP	Control	Genotype method
Discovery (Southern Han Chinese, cohort 1)	930	945	\	5068	HumanOmniZhongHua-8 Bead Chips (Illumina)
Replication (Southern Han Chinese, cohort 2)	724	781	\	3265	Sequenom Mass ARRAY system
Replication (Northern Han Chinese, cohort 3)	417	492	\	1832	Sequenom Mass ARRAY system
Replication (Southern Han Chinese, cohort 4)	\	\	2270	3265	Sequenom Mass ARRAY system
Replication (Northern Han Chinese, cohort 5)	\	\	427	1832	Sequenom Mass ARRAY system
Total	2071	2218	2697	10165	

**Fig 1:**
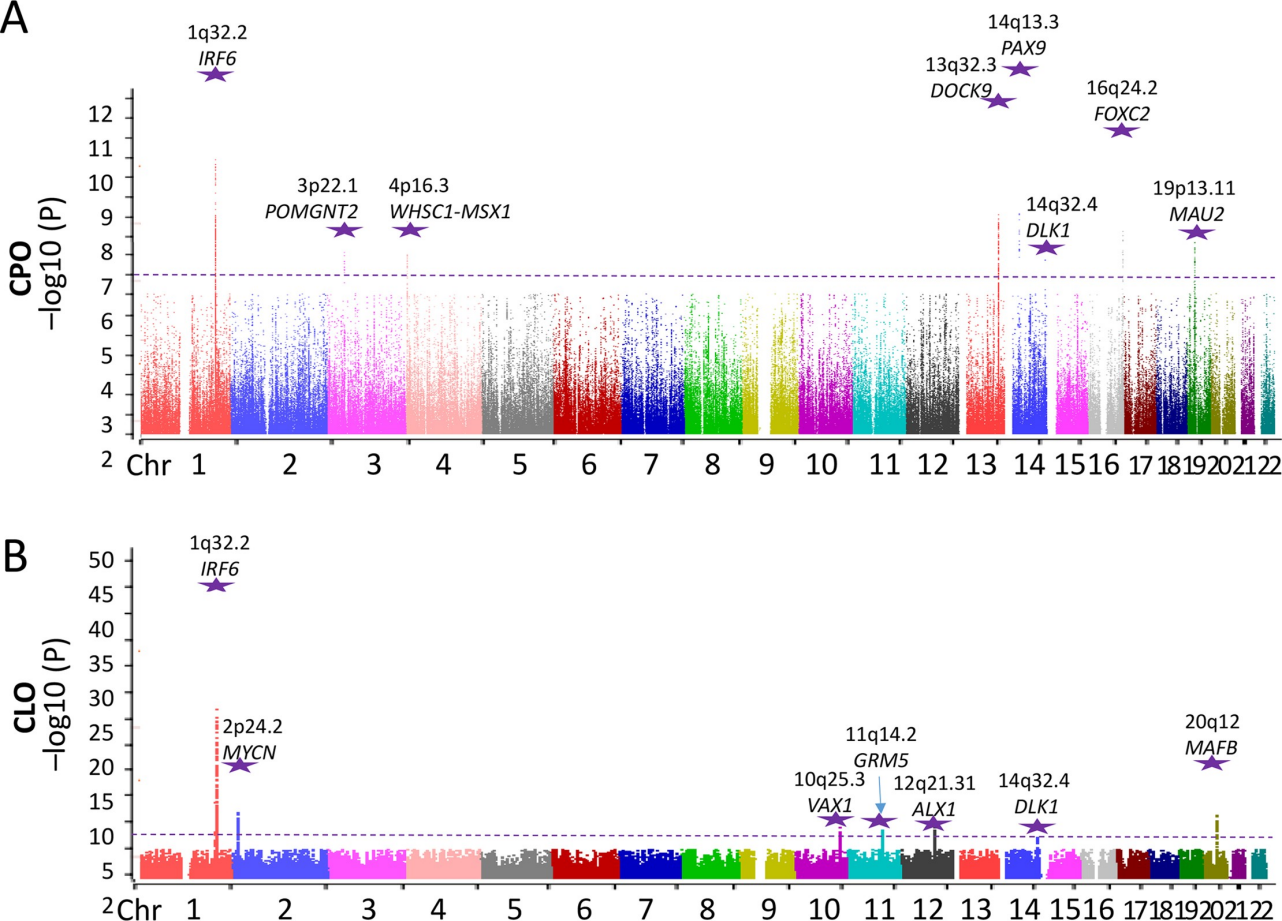
Manhattan plots for genes/loci identified by typical GWAS. Manhattan plot showing the −log_10_*P* values of the association analysis of SNPs passing the Hardy–Weinberg test with a *P* value > 1.0 × 10^−6^. The plot includes GWAS data for the discovery cohort, comprising 930 CPO patients **(A)**, 945 CLO patients **(B)** and 5048 healthy control individuals of Southern Han Chinese ethnicity, plotted against the respective positions of SNPs on the autosomes. The purple dashed line shows the exome-wide significance threshold for this study (*P* = 9 × 10^−7^). Purple stars indicate the results of a final meta-analysis incorporating the GWAS discovery cohort and two replication cohorts, comprising a total of 2071 CPO cases, 2218 CLO cases and 10165 control individuals.

After standard quality-control filtering for the participants and the SNPs (see Methods) and after excluding samples with poor quality and genetic heterogeneity using population stratification analysis, we obtained genotype data for 930 isolated CPO patients, 945 isolated CLO patients and 5,048 control individuals (Table 2, the discovery cohort). The principal component analysis (PCA) results indicated that the remaining cases and controls were genetically well matched, without evidence of gross population stratification (S3 Fig). The genomic inflation factor (λ_GC_) in the discovery cohort was 1.016 for CPO and 1.031 for CLO, suggesting that the association test statistics were not substantially confounded by the population substructure. We performed GWAS analysis using the logistic test with adjustment for sex and PCs (C1, C2, C3 and C4) using PLINK version 1.9 software. To further increase the genome coverage, we performed an imputation analysis to infer the genotypes of additional common SNPs (see Methods). The quantile-quantile (QQ) plots (using R package “qqman”) of the association results are shown in S4 Fig. The Manhattan plot (using R package “qqman”) of the *P* values is shown in Fig 1. CPO did not show a strong association signal in the discovery stage. If we used the normal GWAS significance cutoff, such as *P* < 5 × 10^−8^, we may omit some true association genes at the first replication. The association of CPO would be weak because the previous CPO GWAS analysis based on trios did not discover many CPO main effect genes/loci. Previous studies suggested that the genetic model of CPO should be composed of many minor effect genes/loci. Therefore, we used the *P* < 9 × 10^−7^ cutoff in the discovery stage for SNP selection for the first round replication. A total of 22 loci showed evidence of a significant association with CPO or CLO (i.e., surpassing *P* < 9 × 10^−7^) at the GWAS discovery stage. All the association results of CPO and CLO adjusted for sex and PCs (PC1, PC2, PC3 and PC4) with P values less than e-5 in the discovery stage (935 CPO patients, 948 CLO patients and 5,050 control individuals) were listed in **S1 Data**.

**Table 2:** Genes/loci identified for NSOFC by typical GWAS. Results of typical GWAS for SNPs significant at a multiple-testing correction level (*P* < 5 × 10^−8^) in CPO or CLO by meta-analyses of discovery and replication results.

SNP	Loci	Affected Gene (a)	Alleles	Cohort	Discovery (c) N = 6943			Replication CHS (d) N = 7040			Replication CHB (e) N = 3163			Combined (f) N = 17151		
					MAF (b)	*P*	OR	MAF (b)	*P*	OR	MAF (b)	*P*	OR	*P*	OR	I (g)
**Association results for markers from the novel risk loci (n = 9)**																
rs6791526	3p22.1	*POMGNT2*	T/C	CPO	0.107/0.073	6.23 × 10^−7^	1.53	0.093/0.068	2.13 × 10^−3^	1.40	0.086/0.063	1.43 × 10^−2^	1.4	1.62 × 10^−10^	1.46	0
				CLO	0.083/0.073	0.129	1.15	0.081/0.068	7.19 × 10^−2^	1.21	0.085/0.063	1.37 × 10^−2^	1.39	1.39 × 10^−4^	1.22	0
				CLP				0.078/0.068	3.41 × 10^−2^	1.16	0.060/0.063	0.796	1.047	5.8 × 10^−2^	1.14	0
rs3468	4p16.3	*WHSC1*	G/A	CPO	0.522/0.455	8.28 × 10^−7^	1.33	0.499/0.454	1.24 × 10^−3^	1.20	0.496/0.455	8.73 × 10^−2^	1.18	5.4 × 10^−11^	1.256	20.56
				CLO	0.451/0.455	0.7388	0.99	0.471/0.454	0.252	1.07	0.455/0.455	0.714	1.00	0.57	1.02	0
				CLP				0.474/0.454	5.61 × 10^−2^	1.083	0.460/0.455	0.803	1.02	5.52 × 10^−2^	1.070	0
rs57700751	11q14.2	*GRM5*	C/T	CPO	0.069/0.068	0.87	1.02	0.061/0.055	0.43	1.12	0.062/0.071	0.403	0.868	0.79	1.01	0
				CLO	0.101/0.068	4.75 × 10^−7^	1.547	0.069/0.055	3.92 × 10^−2^	1.287	0.089/0.071	9.2 × 10^−2^	1.27	3.0 × 10^−8^	1.40	16.4
				CLP				0.072/0.055	5.08 × 10^−4^	1.335	0.073/0.071	0.347	1.024	1.2 × 10^−3^	1.25	60.23
rs765366	12q21.31	*ALX1*	G/A	CPO	0.185/0.168	7.1 × 10^−2^	1.125	0.144/0.136	0.786	1.073	0.169/0.163	0.786	1.040	5.8 × 10^−2^	1.09	0
				CLO	0.216/0.168	5.26 × 10^−7^	1.37	0.156/0.136	4.84 × 10^−2^	1.174	0.189/0.163	6 × 10^−2^	1.19	4.30 × 10^−8^	1.27	30.0
				CLP				0.145/0.136	0.181	1.08	0.143/0.163	0.831	0.86	0.58	1.027	77.66
rs4646211	13q32.3	*DOCK9*	C/T	CPO	0.423/0.346	2.82 × 10^−10^	1.38	0.333/0.296	8.71 × 10^−3^	1.184	0.314/0.273	2.84 × 10^−2^	1.22	4.78 × 10^−12^	1.28	50
				CLO	0.348/0.346	0.495	1.00	0.315/0.296	0.181	1.091	0.273/0.273	0.11	1.00	0.42	1.03	0
				CLP				0.311/0.296	0.11	1.07	0.259/0.273	1.11	0.928	0.28	1.04	54.32
rs730643	14q13.3	*PAX9*	G/A	CPO	0.263/0.338	2.84 × 10^−8^	0.70	0.326/0.388	1.26 × 10^−5^	0.76	0.314/0.363	8.90 × 10^−3^	0.804	2.92 × 10^−16^	0.74	7.63
				CLO	0.334/0.338	0.863	0.98	0.372/0.388	0.226	0.93	0.363/0.363	0.792	1.00	0.32	0.97	0
				CLP				0.377/0.388	0.287	0.96	0.382/0.363	0.3	1.083	0.68	0.983	41.14
rs2415363	14q13.3	*PAX9*	A/C	CPO	0.263/0.338	2.84 × 10^−8^	0.70	0.326/0.385	2.17 × 10^−5^	0.77	0.324/0.363	7.03 × 10^−2^	0.841	2.76 × 10^−14^	0.75	44.52
				CLO	0.334/0.338	0.863	0.98	0.356/0.385	4.56 × 10^−2^	0.88	0.36/0.363	0.896	0.987	0.11	0.75	10.89
				CLP				0.380/0.385	0.652	0.97	0.385/0.363	0.241	1.101	0.91	1.00	51.74
rs60708031	14q13.3	*PAX9*	T/G	CPO	0.263/0.338	9.29 × 10^−8^	0.70	0.323/0.384	1.70 × 10^−5^	0.76	0.317/0.360	2.03 × 10^−2^	0.825	4.39 × 10^−15^	0.75	29.66
				CLO	0.334/0.338	0.932	0.983	0.363/0.384	0.136	0.915	0.360/0.360	0.764	1.00	0.28	0.96	0
				CLP				0.392/0.384	0.387	1.035	0.388/0.360	0.173	1.125	0.28	0.996	0
rs730570	14q32.2	*DLK1*	A/G	CPO	0.243/0.197	1.71 × 10^−5^	1.309	0.240/0.204	3.32 × 10^−3^	1.228	0.225/0.181	3.32 × 10^−3^	1.34	6.59 × 10^−10^	1.28	0
				CLO	0.248/0.197	6.14 × 10^−7^	1.344	0.233/0.204	1.50 × 10^−2^	1.18	0.206/0.181	8.70 × 10^−2^	1.18	2.53 × 10^−8^	1.253	22.1
				CLP				0.219/0.204	7.84 × 10^−2^	1.09	0.159/0.181	0.184	0.856	0.31	1.045	77.77
rs72812203	16q24.2	*FOXC2-FOXL1*	G/T	CPO	0.276/0.213	8.90 × 10^−8^	1.40	0.239/0.213	2.83 × 10^−2^	1.16	0.237/0.201	2.83 × 10^−2^	1.23	4.2× 10^−10^	1.28	56.83
				CLO	0.253/0.213	1.13 × 10^−3^	1.174	0.237/0.213	3.88 × 10^−2^	1.15	0.189/0.201	0.646	1.00	2.4× 10^−3^	1.129	14.61
				CLP				0.221/0.213	0.321	1.046	0.189/0.201	0.646	0.924	0.63	1.13	24.41
rs8061677	16q24.2	*FOXC2-FOXL1*	C/T	CPO	0.242/0.205	5.79 × 10^−8^	1.40	0.234/0.207	2.10 × 10^−2^	1.17	0.231/0.196	2.10 × 10^−2^	1.29	9.11 × 10^−11^	1.29	48.84
				CLO	0.241/0.205	1.18 × 10^−4^	1.23	0.235/0.207	1.52 × 10^−2^	1.180	0.196/0.196	0.951	0.991	2.3 × 10^−4^	1.16	50.49
				CLP				0.226/0.207	1.52 × 10^−2^	1.12	0.194/0.196	0.951	0.991	3.7 × 10^−2^	1.09	23.25
rs1009136	19p13.11	*MAU2*	A/G	CPO	0.359/0.295	8.84 × 10^−7^	1.34	0.341/0.311	2.90 × 10^−2^	1.14	0.330/0.288	1.52 × 10^−2^	1.21	2.66 × 10^−9^	1.25	46.17
				CLO	0.309/0.295	2.2 × 10^−2^	1.069	0.307/0.311	0.721	0.98	0.289/0.288	0.84	1.00	0.51	1.02	0
				CLP				0.295/0.311	7.03 × 10^−2^	0.927	0.295/0.288	0.689	1.035	0.27	0.95	22.4
**Association results for markers from the replicated risk loci (n = 4)**																
rs2235371	1q32.2	*IRF6*	T/C	CPO	0.472/0.405	3.49 × 10^−7^	1.314	0.471/0.390	2.07 × 10^−6^	1.394	0.373/0.378	0.819	0.9817	1.90 × 10^−9^	1.254	33.72
				CLO	0.268/0.405	1.44 × 10^−26^	0.5387	0.273/0.390	1.78 × 10^−13^	0.588	0.242/0.378	6.25 × 10^−13^	0.528	1.25 × 10^−48^	0.551	0
				CLP				0.319/0.390	2.49 × 10^−12^	0.7335	0.263/0.378	1.79 × 10^−9^	0.5886	4.50 × 10^−19^	0.702	39.68
rs2235377	1q32.2	*IRF6*	C/T	CPO	0.483/0.412	7.98 × 10^−8^	1.333	0.479/0.392	2.16 × 10^−4^	1.426	0.479/0.384	2.16 × 10^−4^	1.43	1.02 × 10^−13^	1.368	0
				CLO	0.273/0.412	2.89 × 10^−27^	0.5356	0.275/0.392	8.91 × 10^−7^	0.589	0.250/0.384	7.55 × 10^−6^	0.535	3.70 × 10^−36^	0.546	0
				CLP				0.225/0.392	2.89 × 10^−27^	0.5356	0.251/0.384	7.55 × 10^−6^	0.535	4.23 × 10^−31^	0.536	0
rs12405750	1q32.2	*IRF6*	T/C	CPO	0.483/0.412	7.98 × 10^−8^	1.333	0.480/0.393	3.47 × 10^−7^	1.427	0.389/0.387	0.985	1.001	8.80 × 10^−11^	1.276	33.91
				CLO	0.272/0.412	1.82 × 10^−27^	0.5341	0.278/0.393	1.29 × 10^−12^	0.6014	0.249/0.387	2.92 × 10^−13^	0.526	8.13 × 10^−49^	0.553	1.66
				CLP				0.321/0.393	1.21 × 10^−12^	0.7304	0.268/0.387	6.22 × 10^−10^	0.5819	9.56 × 10^−20^	0.698	31.14
rs10863790	1q32.2	*IRF6*	C/A	CPO	0.481/0.411	1.47 × 10^−7^	1.325	0.483/0.387	5.04 × 10^−5^	1.481	0.379/0.387	0.762	0.9723	1.20 × 10^−8^	1.27	32.47
				CLO	0.272/0.411	1.30 × 10^−27^	0.5331	0.279/0.387	1.13 × 10^−7^	0.611	0.246/0.387	1.30 × 10^−9^	0.52	1.16 × 10^−40^	0.548	0
				CLP				0.321/0.387	5.51 × 10^−10^	0.749	0.2676/0.387	7.00 × 10^−10^	0.5823	7.64 × 10^−17^	0.709	34.24
rs72741048	1q32.2	*IRF6*	T/A	CPO	0.588/0.506	8.64 × 10^−10^	1.392	0.588/0.510	8.18 × 10^−7^	1.36	0.5158/0.493	0.321	1.094	3.07 × 10^−15^	1.314	48.33
				CLO	0.368/0.506	9.56 × 10^−26^	0.5665	0.37/0.510	1.39 × 10^−8^	0.612	0.352/0.493	2.85 × 10^−9^	0.558	8.22 × 10^−40^	0.575	0
				CLP				0.418/0.510	9.43 × 10^−11^	0.749	0.3937/0.493	3.91 × 10^−7^	0.664	5.20 × 10^−16^	0.728	10.62
rs189000	1q32.2	*IRF6*	A/G	CPO	0.349/0.419	9.55 × 10^−8^	0.7438	0.341/0.408	9.62 × 10^−5^	0.752	0.388/0.410	0.237	0.9099	2.28 × 10^−10^	0.782	17.32
				CLO	0.499/0.419	1.14 × 10^−9^	1.381	0.505/0.408	1.91 × 10^−9^	1.484	0.476/0.410	7.56 × 10^−4^	1.3	1.17 × 10^−19^	1.395	0
				CLP				0.468/0.408	6.81 × 10^−9^	1.279	0.476/0.410	7.97 × 10^−4^	1.31	2.39 × 10^−11^	1.285	0
rs1387934	1q32.2	*IRF6*	T/C	CPO	0.349/0.418	1.17 × 10^−7^	0.7453	0.342/0.409	9.25 × 10^−5^	0.752	0.391/0.411	0.298	0.9203	4.06 × 10^−10^	0.785	21.52
				CLO	0.500/0.418	6.95 × 10^−10^	1.387	0.506/0.409	2.12 × 10^−9^	1.482	0.476/0.411	7.45 × 10^−4^	1.3	7.47 × 10^−20^	1.397	0
				CLP				0.468/0.409	1.16 × 10^−8^	1.274	0.479/0.411	4.58 × 10^−4^	1.32	2.54 × 10^−11^	1.284	0
rs4832468	2p24.2	*MYCN*	C/T	CPO	0.2516/0.2913	9.63 × 10^−4^	0.818	0.233/0.281	2.46 × 10^−2^	0.777	0.233/0.288	2.46 × 10^−2^	0.777	5.38 × 10^−6^	0.803	0
				CLO	0.213/0.2913	3.24 × 10^−11^	0.6584	0.204/0.281	3.41 × 10^−4^	0.6525	0.201/0.288	1.88 × 10^−3^	0.624	5.21 × 10^−16^	0.653	0
				CLP				0.178/0.282	3.24 × 10^−12^	0.6584	0.201/0.288	1.88 × 10^−3^	0.624	9.37 × 10^−11^	0.652	0
rs7552	2p24.2	*MYCN*	A/G	CPO	0.254/0.292	1.39 × 10^−3^	0.8236	0.229/0.284	1.11 × 10^−2^	0.7488	0.229/0.290	1.11 × 10^−2^	0.749	2.52 × 10^−6^	0.796	0
				CLO	0.211/0.292	7.23 × 10^−12^	0.6489	0.199/0.284	1.01 × 10^−4^	0.625	0.204/0.290	3.32 × 10^−3^	0.628	6.44 × 10^−17^	0.642	0
				CLP				0.178/0.284	3.23 × 10^−12^	0.648	0.204/0.290	3.32 × 10^−3^	0.628	8.25 × 10^−14^	0.645	0
rs7902527	10q25.3	*VAX1*	A/G	CPO	0.297/0.279	0.15	1.088	0.275/0.303	0.202	0.8724	0.275/0.287	0.202	0.8724	0.977	1.001	22.39
				CLO	0.346/0.279	3.21 × 10^−8^	1.366	0.335/0.303	4.0 × 10^−2^	1.23	0.301/0.287	0.619	1.07	3.15 × 10^−8^	1.273	10.25
				CLP				0.194/0.303	3.21 × 10^−8^	1.366	0.301/0.287	0.619	1.07	1.25 × 10^−7^	1.318	23.82
rs4752028	10q25.3	*VAX1*	C/T	CPO	0.343/0.334	0.483	1.04	0.348/0.347	0.959	1.005	0.348/0.321	0.959	1.005	0.556	1.026	0
				CLO	0.408/0.334	3.62 × 10^−9^	1.378	0.395/0.347	3.90 × 10^−2^	1.23	0.367/0.32	0.113	1.23	2.58 × 10^−10^	1.328	0
				CLP				0.450/0.347	3.62 × 10^−9^	1.378	0.367/0.32	0.113	1.23	1.55 × 10^−9^	1.354	0
rs17820943	20q12	*MAFB*	T/C	CPO	0.434/0.456	0.105	0.9166	0.435/0.464	0.224	0.8878	0.476/0.482	0.776	0.9748	5.65 × 10^−2^	0.924	0
				CLO	0.370/0.456	6.95 × 10^−11^	0.7015	0.372/0.464	0.40 × 10^−4^	0.482	0.404/0.482	1.05 × 10^−3^	0.729	2.08 × 10^−17^	0.702	0
				CLP				0.378/0.464	0.38 × 10^−14^	0.482	0.385/0.482	1.02 × 10^−6^	0.673	3.77 × 10^−20^	0.695	0

(a) Most possible susceptibility genes in this region

(b) MAF: Affected/Unaffected

(c) 930 CPO, 945 CLO and 5048 Controls

(d) 724 CPO, 781 CLO, 2,270 CLP and 3,265 Controls

(e) 417 CPO, 492 CLO, 427 CLP and 1,832 Controls

(f) totally 17,151 samples: 2,071 CPO, 2,218 CLO, 2,697 CLP and 10,165 Controls

(g) I^2^ heterogeneity. CHS: Southern Chinese; CHN: Northern Chinese.

Additionally, 22 previously reported loci including 32 SNPs responsible for CL/P, CPO or CLP also showed marginal association (*P* < 0.05) with CPO or CLO (S1 Table). Among these loci, two reported CPO SNPs rs61776460 in 1p36.11 (*GRHL3*, *P* = 9.31× 10^−3^) and rs604328 in 5p13.2 (*UGT3A2*, *P* = 3.15× 10^−3^) are weakly associated with CPO in this study [28]. For the four reported CPO SNPs by Butali A *et al*. (rs80004662 and rs113691307 in *CTNNA2*, rs62529857 in *SULT2A1* and rs2325377 in *DACH1* [29], we could not find these SNPs in our chips. Two reported CLP SNPs rs908822 in 4q28.1 (*LOC285419*, *P* = 2.34 × 10^−3^) and rs13317 in 8p11.23 (*BAG4/FGFR1*, *P* = 4.21 × 10^−3^)[24] are weakly associated with CPO in this study. Two reported CL/P SNPs rs987525 in 8q24.21 (*AC068570*.*1*, *P* = 5.53 × 10^−3^)[11, 13] [13, 16]and rs60417080 in 13q31.1 (*RP11-501G7*.*1*, *P* = 8.83 × 10^−3^)[15, 16, 18] are also weakly associated with CPO in this study. Reported CLP SNPs rs560426 and rs66515264 in 1p22.1–21.3 (*ARHGAP29*, *P* = 1.79 × 10^−3^ and *P* = 4.83 × 10^−3^ respectively)[28], as well as rs1034832 in 8q21.3 (*DCAF4L2/CTB-118P15*.*2*, *P* = 2.60 × 10^−5^)[24] are weakly associated with CLO. In the same locus, rs12543318 which was reported associated with CL/P, is also weakly associated with CLO (*P* = 3.79 × 10^−5^)[15, 16]. Three more reported CL/P SNPs, rs8049367 in 16p13.3 (*RP11-462G12*.*2*, *P* = 2.95 × 10^−4^)[19], rs4791774 in 17p13.1 (*NTN1*, *P* = 5.87 × 10^−5^)[19] and rs227731 in 17q22 (*NOG*, P = 4.35 × 10^−4^)[15, 16, 18] showed weakly association with CLO in this study.

In order to replicate the associations that arose from our discovery cohort, we first selected 48 SNPs in the 22 discovered loci mentioned above for replication assays (see Methods, S2 Table) among 724 of the CPO patients, 781 of the CLO patients and 3,265 of the control individuals (the Southern Chinese replication cohort). Thirty-two SNPs in 15 loci maintained statistically significant associations with CPO or CLO (*P* < 0.05). We then genotyped these 32 SNPs in a secondary replication cohort of Northern Chinese ethnicity, comprising 417 unrelated CPO patients, 492 unrelated CLO patients and 1,832 unrelated normal control individuals.

**Fig 2:**
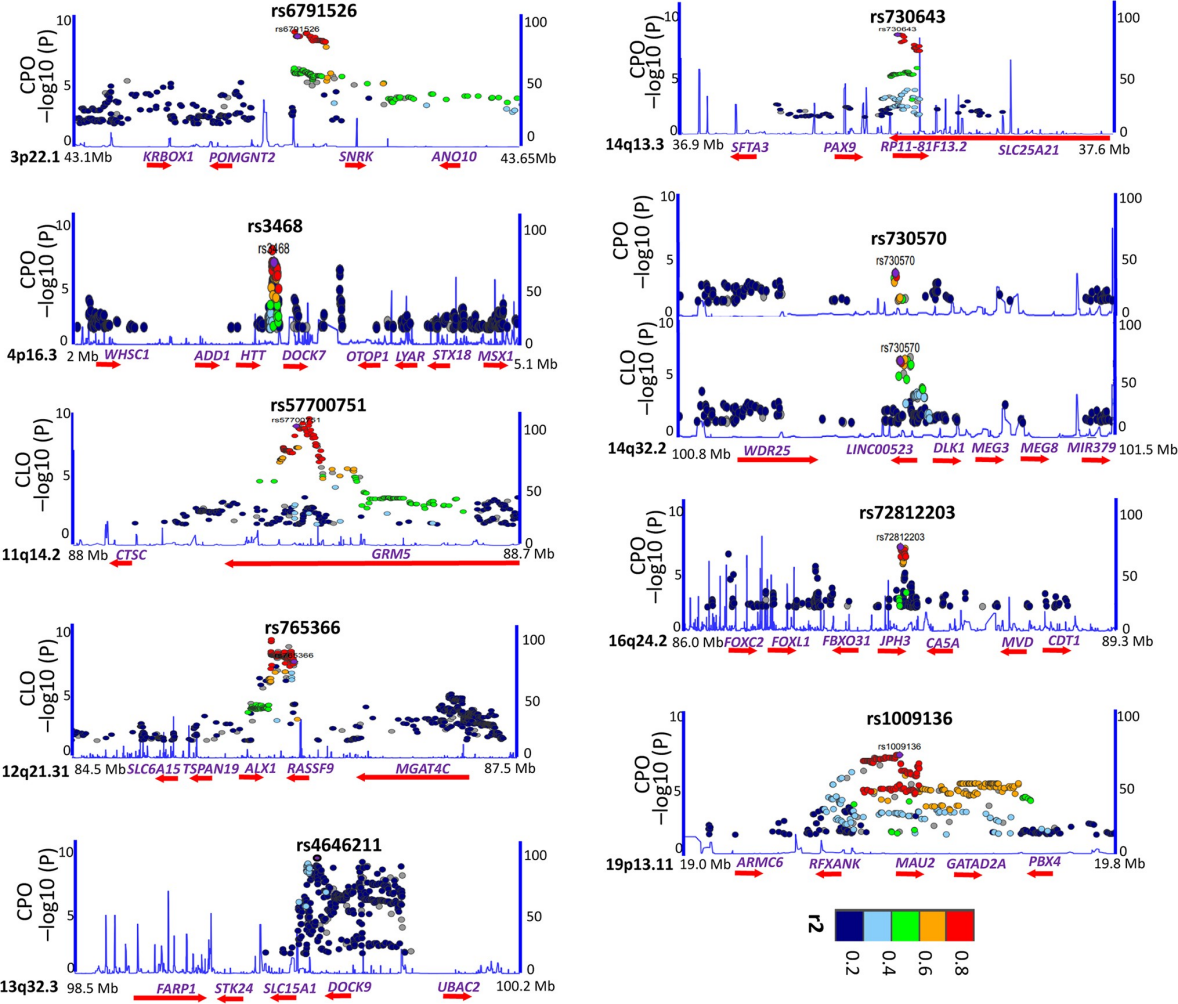
Regional association for the nine newly discovered loci by typical GWAS. Regional association plots indicate the −log_10_*P* values of the genotyped SNPs of each locus. The sequence data were aligned to human hg19. The Y axis represents the negative logarithm (base 10) of the SNP *P* value and the X axis represents the position on the chromosome, with the name and location of genes in the UCSC Genome Browser shown in the bottom panel. The SNP with the lowest meta analyzed *P* value in the region was marked by a purple star. The colors of the other SNPs indicate the r^2^ of these SNPs with the lead SNP. Plots were generated with LocusZoom using hg19/1000 genome build LD for ASN population (2014).

We performed a meta-analysis of all the three cohorts and yielded eight genes/loci associated with CPO that surpassed genome-wide statistical significance (*P* < 5.0 × 10^−8^) (Fig 1A, Fig 2 and Table 2). One of the eight genes/loci, *IRF6*, was previously reported to be associated with CPO: *IRF6* (rs12405750, *P* = 8.8 × 10^−11^). Meanwhile, seven genes/loci were novel: *POMGNT2* (rs6791526, *P* = 1.62 × 10^−10^), *WHSC1* (rs3468, *P* = 5.4 × 10^−11^), *DOCK9* (rs4646211, *P* = 4.78 × 10^−12^), *PAX9* (rs730643, *P* = 2.92 × 10^−16^), *DLK1* (rs730570, *P* = 6.59 × 10^−10^), *FOXC2-FOXL1* (rs72812203, *P* = 4.2 × 10^−10^) and *MAU2* (rs1009136, *P* = 2.66 × 10^−9^). Seven genes/loci were associated with CLO that surpassed genome-wide statistical significance (Fig 1B, Fig 2 and Table 2); while three were novel: *GRM5* (rs57700751, *P* = 3.0 × 10^−8^), *ALX1* (rs765366, *P* = 4.30 × 10^−8^) and *DLK1* (rs730570, *P* = 2.53 × 10^−8^); and four were previously reported to be associated with CL/P: *IRF6* (rs12405750, *P* = 8.13 × 10^−49^), *MYCN* (rs4832468, *P* = 5.21 × 10^−16^), *VAX1* (rs4752028, *P* = 3.15 × 10^−8^) and *MAFB* (rs17820943, *P* = 2.08 × 10^−17^). We noted that *DLK1* and *IRF6* were associated with both CPO and CLO (Table 2), therefore, 13 genes/loci were identified as being associated with CPO or CLO and nine of them are novel.

Furthermore, to assess whether these CPO or CLO associated 13 genes/loci were also associated with CLP, we genotyped the 24 SNPs in the 13 genes/loci in an independent cohort consisting of 2,270 unrelated sporadic cases of CLP and 3,265 control individuals of the Southern Han Chinese population, along with 427 CLP patients and 1,832 control individuals of the Northern Han Chinese population (Table 2). However, we found that only the four genes (*IRF6*, *MYCN*, *VAX1* and *MAFB*) that were previously reported to be associated with CL/P were significantly associated with CLP (*P* < 5 × 10^−8^). These results demonstrate that the novel genes can only be identified by the specific NSOFC subtypes for association studies, and the genetic factors for CL/P that were previously identified are closer to the genetic factors for CLO and CLP than for CPO.

### Expression dosage effect of *IRF6* with different alleles associated with CPO or CLO

**Fig 3:**
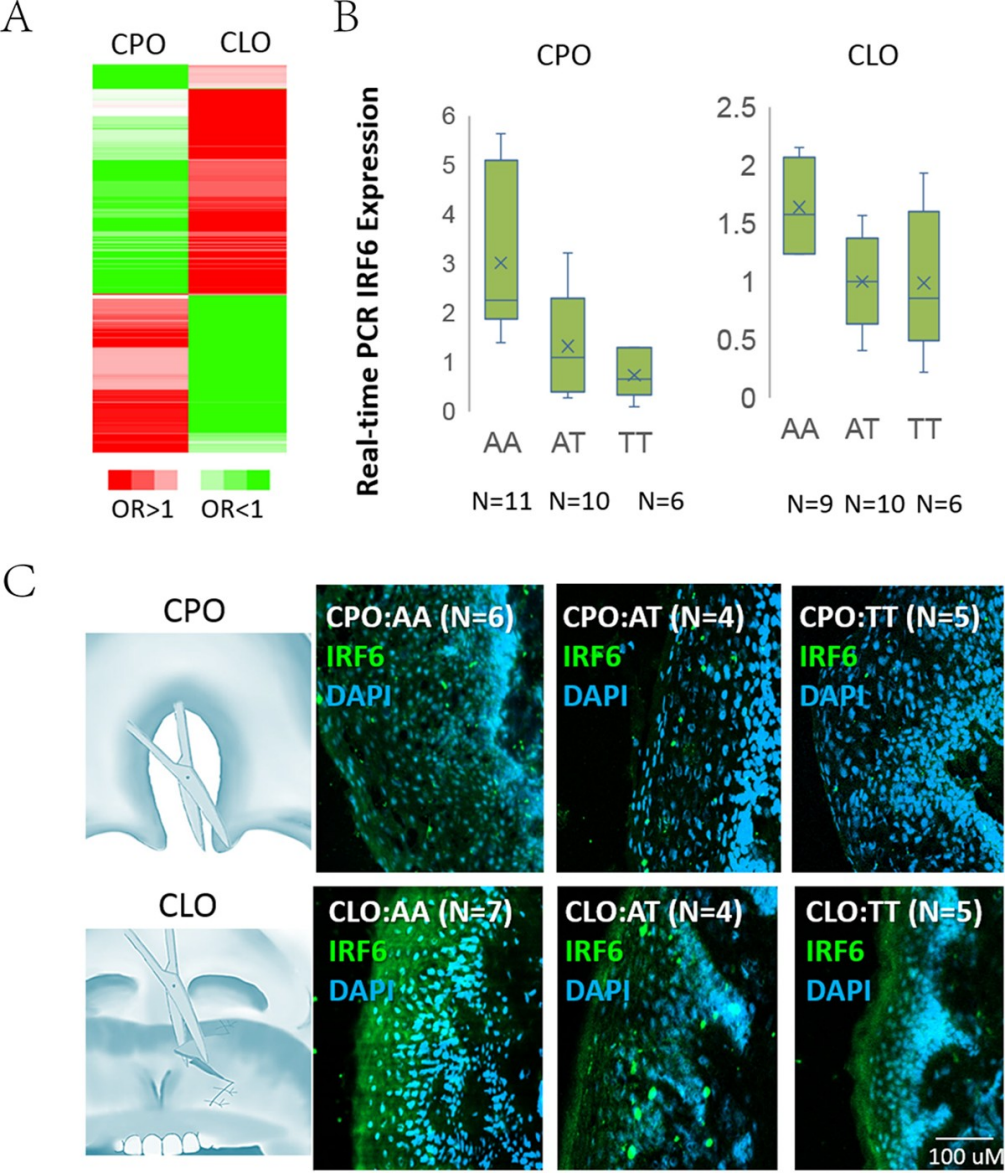
*IRF6* gene expression in CPO and CLO affected tissues. **(A)** Cluster of the odds ratios of 537 significant markers (*P*< 9×10^−7^) for CPO or CLO in the *IRF6* locus LD in the discovery stage using Cluster 3.0. **(B)** Gene expression of *IRF6* for rs72741048 in 1q32.2. Box plots of ratios of AA, AT genotypes comparing to TT genotype. Using real time PCR to detect gene expression in rim of uvula tissues of CPO patients and edge of upper lip cleft tissues of CLO patients. **(C)** Immunofluorescence of IRF6 in affected tissues. Left panels: IRF6 (green) was expressed in the tissues from the rim of the uvula of CPO patients (upper) and edge of the upper lip cleft of CLO patients (lower panel). Right panels: Box plots of ratios of fluorescence values of AA and AT genotypes comparing to TT genotypes. Fluorescence values were measured with ImageJ software.

Consistent with previous findings [11, 16, 21–23], we also confirmed that the *IRF6* gene had the strongest association with both CPO and CLO, supported by the 537 statistically significant SNPs in this region (*P* < 9 × 10^−7^) that were identified in the discovery stage (Fig 3A, S3 Table). After the meta-analysis of the results from the discovery and replication cohorts, the *IRF6* gene region showed the most significantly associated SNPs with either CPO or CLO (Table 2B, Fig 1). However, the contributions of *IRF6* to CPO and CLO were distinctly different based on the following findings: 1) the two alleles of the same associated SNP in the *IRF6* showed an opposite direction of association between CPO and CLO, and 2) the SNPs in the *IRF6* showed a stronger signal of association with CLO in comparison to CPO. For example, the T allele in rs72741048, had *P* = 3.07 × 10^−15^ and odds ratio (OR) = 1.314 for CPO; while it had *P* = 8.22 × 10^−40^ and OR = 0.575 for CLO (Fig 1, Table 2).

Convergent evidence from CPO and CLO points to the *IRF6* gene as being a critical factor for the pathogenesis of NSOFC. Most of the SNPs associated with CPO and CLO are located in the 5’UTR and intronic regions of *IRF6*, which contain enrichment signals of active transcription start sites, transcriptions, enhancers and ChIP-seq chromatin profiling signals (S4 Table, S5 Fig). This suggests that these regulatory elements might control *IRF6* gene expression.

We next sought to determine whether the associated variants for CPO and CLO can affect the levels of *IRF6* gene expression in human CPO or CLO disease tissues. *IRF6* gene expression in palatine uvula mucosa from 64 patients with CPO and the edge of the upper lip cleft from 49 patients with CLO were assessed, separately, using real-time polymerase chain reaction (PCR). For example, in the SNP rs72741048, T was a risk allele for CPO, but it was a protective allele for CLO. We also found that *IRF6* was down-regulated 1.6 times in the TT genotype in comparison to the AA genotype in CPO, but it was down-regulated 4.1 times in the TT genotype in comparison to the AA genotype in CLO (Fig 3B). In addition, *IRF6* was down-regulated about 2.0 times in the TT genotype in comparison to the AA genotype in most of the normal tissues (Genotype-Tissue Expression (GTEX) data) (see the list of URLs), suggesting that a relatively low expression level of *IRF6* is a risk for CPO but is protective for CLO in the affected tissues. The genotype-specific expression patterns were also confirmed at the protein level by immunohistochemistry in the 31 patient-derived tissue samples (Fig 3C). It should be mentioned that the expression of *IRF6* was higher in the edge of the upper lip cleft tissue (the main area affected in CLO condition) than in the uvula tissue (the main area affected in CPO condition) in the normal condition (Fig 3C). Although the sample size was limited, these test results suggest that different CPO and CLO phenotypes are partially associated with dosage imbalances in the gene expression of *IRF6* in the disease-related tissues.

### Five genes/loci were identified by gene network and ontology analysis, and further replications

Our typical GWAS results revealed the importance of developmental transcription factors (TFs) in the regulation of the disease direction (CPO versus CLO), as evidenced by the association signals found near the following TFs: *WHSC1*, *PAX9*, *FOXC2*, *IRF6*, *MYCN*, *VAX1* and *MAFB*. Given the limited sample size in the genetic study, some true disease genes could be missed by the typical GWAS analysis due to insufficient power. To explore more potential associated genes for CPO or CLO in our data, we conducted the following five steps for further analysis:

1. We conducted first round candidate of a set of NSOFC candidate genes from published references [1, 2, 5], the GWAS Catalog, the Human Gene Mutation Database, Phynolizer [30] and Human Phenotype Ontology (see URLs; see Methods).
2. We conducted second round candidate of NSOFC genes using GeneMANIA network analysis [31] and ontology, as well as pathway analysis using Database for Annotation, Visualization and Integrated Discovery (DAVID) [32] with the first round candidate NSOFC genes as queries to obtain more functional related genes. A total of 243 genes were enriched either by interaction with the genetic factors or in the same ontology as the candidate genes for NSOFC. The gene interaction and ontology analysis of the enriched genes are shown in S5 Table, S6 Fig and S7 Fig.
3. We next explored whether these candidate genes were associated with CPO or CLO, using our GWAS datasets. In addition to the identified association genes/loci in our typical GWAS analysis as we described in the first part of our results, we also found an additional 44 SNPs in 43 of the 243 enriched gene regions with at least one SNP in CPO or CLO surpassing *P* < 2.06 × 10^−4^ (*P* < 0.05/243 *=* 2.06 × 10^−4^ for Bonferroni correction) in the discovery stage. This suggests that these enriched genes might also have genetic contributions to CPO or CLO.
4. In order to confirm the associations that arose from step (3), we validated these 44 SNPs using Sequenom MassARRAY genotyping and association analysis in the Southern Han Chinese ethnicity replication cohort (724 CPO patients, 781 CLO patients, 2,270 CLP patients and 3,265 control individuals) and Northern Han Chinese ethnicity cohort (417 unrelated CPO patients, 492 unrelated CLO patients, 427 CLP patients and 1,832 unrelated normal controls).
5. We conducted the final meta-analysis of the discovery and replications cohorts.

Five novel genes/loci surpassed genome-wide statistical significance (*P* < 5 × 10^−8^) in the final meta-analysis of the discovery and replications cohorts (Table 3), with three for CPO and two for CLO. For CPO: rs72688980 in 4q32.1 between *CTSO* and *PDGFC* (*P* = 1.89 × 10^−8^, OR = 1.456), rs698406 in 7q32.1 in *PAX4* (*P* = 6.18 × 10^−9^, OR = 0.812) and rs78669990 near *FOXF1* in 16q24.1 (*P* = 1.71 × 10^−8^, OR = 1.299). For CLO: rs116910459 near *IRF2* in 4q35.1 (*P* = 4.31 × 10^−8^, OR = 1.32) and rs625882 in 5q35.2 between *FGFR4* and *NSD1* (*P* = 2.28 × 10^−8^, OR = 1.208). Interestingly, four of the five genes/loci contain TFs (*IRF2*, *NSD1*, *PAX4* and *FOXF1*), again suggesting that the TFs play important roles in the development of CPO or CLO. The validation results of the other SNPs that did not surpass the genome-wide statistical significant level are listed in S6 Table.

**Table 3:** Genes/loci for NSOFC identified by gene network and ontology analysis and further replications. CPO, CLO or CLP associated SNPs selected by gene network and ontology and replication analysis which reached significant at a multiple-testing correction level (*P* < 5 × 10^−8^) in CPO or CLO by meta-analysis of discovery and replication results.

SNP	Cytoband	Affected Gene ^(a)^	Alleles	Cohort	Discovery ^(c)^			Replication CHS ^(d)^			Replication CHB ^(e)^			Combined ^(f)^		
					MAF ^(b)^	*P*	OR	MAF ^(b)^	*P*	OR	MAF ^(b)^	*P*	OR	*P_meta*	OR_meta	I^g^
**Association results for markers from the novel risk loci enriched by network analysis (n = 5)**																
rs72688980	4q32.1	*CTSO-PDGFC*	G/A	CPO	0.045/0.026	8.49 × 10^−6^	1.80	0.20/0.092	1.27 × 10^−3^	1.347	0.08/0.061	5.27 × 10^−2^	1.34	1.89 × 10^−8^	1.456	46.94
				CLO	0.041/0.026	6.13 × 10^−4^	1.63	0.11/0.092	2.16 × 10^−2^	1.23	0.080/0.061	4.79 × 10^−2^	1.307	1.06 × 10^−6^	1.331	0
				CLP				0.10/0.092	0.603	1.041	0.07/0.061	0.603	1.16	7.35 × 10^−2^	0.871	33.96
rs116910459	4q35.1	*IRF2-LINC02427*	C/T	CPO	0.136/0.104	2.84 × 10^−5^	1.37	0.123/0.102	2.58 × 10^−2^	1.22	0.139/0.113	8.79 × 10^−2^	1.27	1.60 × 10^−5^	1.314	0
				CLO	1.142/0.104	1.00 × 10^−6^	1.43	0.128/0.102	5.5 × 10^−3^	1.287	0.137/0.113	4.23 × 10^−2^	1.25	4.31× 10^−8^	1.32	0
				CLP				0.108/0.102	0.337	1.061	0.117/0.113	0.337	1.04	0.217	0.908	48.29
rs625882	5q35.2	*FGFR4-NSD1*	T/C	CPO	0.410/0.412	0.83	0.99	0.368/0.413	1.41 × 10^−3^	0.828	0.446/0.441	0.425	1.02	1.28× 10^−3^	1.127	44.64
				CLO	0.457/0.412	2.48 × 10^−4^	1.20	0.439/0.413	6.06 × 10^−3^	1.19	0.498/0.441	1.09× 10^−3^	1.256	2.28 × 10^−8^	1.208	0
				CLP				0.416/0.413	0.775	1.01	0.464/0.441	0.775	1.09	6.14× 10^−2^	1.113	0
rs698406	7q32.1	*PAX4*	G/C	CPO	0.345/0.393	9.56 × 10^−5^	0.813	0.330/0.372	2.5 × 10^−3^	0.83	0.330/0.395	5.60 × 10^−3^	0.78	6.18 × 10^−9^	0.812	0
				CLO	0.373/0.393	0.104	0.92	0.390/0.372	0.203	1.077	0.395/0.395	0.579	1.00	0.498	1.026	0
				CLP				0.365/0.372	0.433	0.969	0.384/0.395	0.433	0.96	0.228	1.061	38.71
rs78669990	16q24.1	*FENDRR-FOXF1*	T/C	CPO	0.155/0.116	1.08 × 10^−6^	1.39	0.138/0.112	5.22 × 10^−3^	1.273	0.147/0.121	7.39 × 10^−2^	1.25	1.71× 10^−8^	1.299	0
				CLO	0.139/0.116	4.50 × 10^−3^	1.22	0.111/0.112	0.922	0.992	0.121/0.121	0.892	0.982	1.35 × 10^−3^	1.221	48.74
				CLP				0.118/0.112	0.307	1.065	0.129/0.121	0.307	1.07	0.576	1.039	0

(a) Most possible susceptibility genes in this region

(b) MAF: Affected/Unaffected

(c) 930 CPO, 945 CLO and 5048 Controls

(d) 724 CPO, 781 CLO, 2,270 CLP and 3,265 Controls

(e) 417 CPO, 492 CLO, 427 CLP and 1,832 Controls

(f) totally 17,151 samples: 2,071 CPO, 2,218 CLO, 2,697 CLP and 10,165 Controls

(g) I^2^ heterogeneity. CHS: Southern Chinese; CHN: Northern Chinese.

## Discussion

In this study, we showed the advantage of using GWAS combined with gene network and ontology analysis to identify genetic factors for NSOFC subtypes. We identified 13 genes/loci for NSOFC by using the typical GWAS method, which followed discovery (*P* < 9 × 10^−7^) and replication steps to obtain the significant genes/loci (*P* < 5 × 10^−8^ for combined results of discovery and replications). We also identified five additional genes/loci for NSOFC (*P* < 5 × 10^−8^ for the combined discovery and replication cohort results), which might be missed by typical GWAS analysis, by the combined method. In the later method, we applied a combination strategy to mine the possible true disease genes by using network and ontology analysis plus further genotype validation.

**Fig 4:**
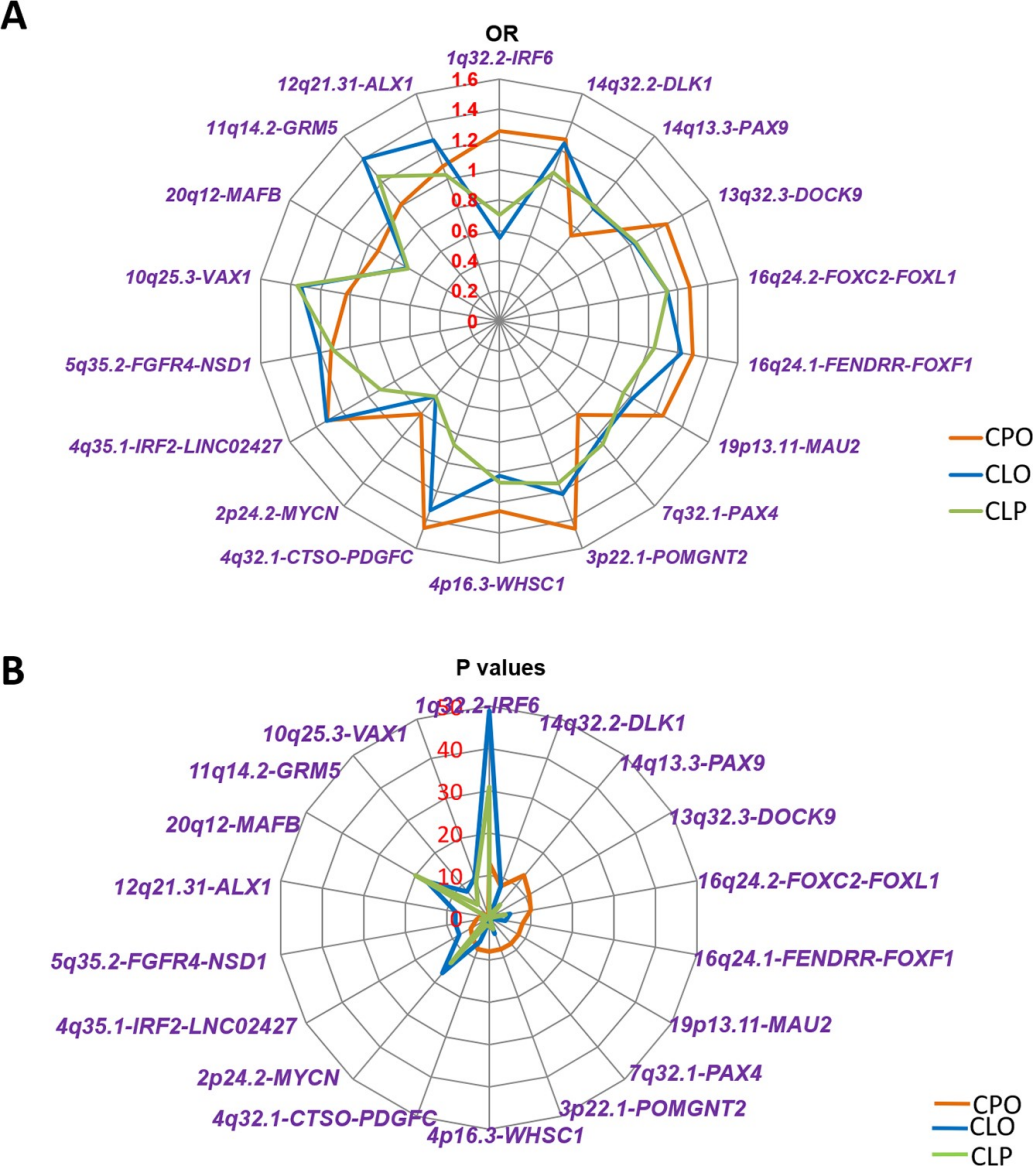
The contribution analysis of associated genes/loci identified in this study for subtypes of NSOFC. TFs were colored in dark pink and other genes were colored in black. The values of the picture were the ORs **(A)** and the −log_10_*P* values **(B)** of the meta results of the most significant SNP of that gene (adjusted for sample size). The genetic association patterns of CLO and CLP were much more similar when compared with that of CPO **(C)**. Some genes/loci were more specific to CPO.

In all, we identified 11 genes/loci for CPO, 10 of which were novel. We also identified nine genes/loci for CLO, five of which were novel. Among these 18 genes (14 of which were novel), *IRF6* and *DLK1* (novel) were associated with both CPO and CLO, *IRF6* was associated with CLP and *MYCN*, *VAX1* and *MAFB* were associated with CLO and CLP. By comparing the *P* values and ORs of CPO, CLO and CLP subtypes, we found that the genetic pattern of CLO is more similar to that of CLP than that of CPO (Fig 4A and 4B). This phenomenon was consistent with a recent study by Carlson JC *et al*., which showed more significant association signals in CLP vs. CP group rather than CL vs. CLP group [28]. Besides *IRF6* locus, CLO also shared *VAVX*, *MAFB* and *MYCN* loci. This finding is consistent with a previous CLP GWAS that CLP and CLO shared more genetic factors [24].

Our results also revealed the importance of developmental TFs in the pathogenesis of these three NSOFC subtypes (Fig 4). We identified 12 genes/loci containing TFs that contribute to CL/P. These TFs include seven families:

1. Interferon regulatory factors: *IRF6* and *IRF2*. *IRF6* was the strongest associated gene for all three subtypes: CPO, CLO and CLP; while *IRF2* was found to significantly contribute to CLO.
2. Paired box TFs: *PAX9* and *PAX4*. These two TFs played roles in the development of CPO specifically.
3. Forkhead box TFs: *FOXC2* and *FOXF1*. Both of these mainly contributed to CPO.
4. Homeobox TFs: *VAX1* and *ALX1*. *VAX1* was found to be associated with both CLO and CLP, while *ALX1* mainly contributed to CLO.
5. Nuclear receptor-binding SET domain proteins: *WHSC1* (also known as *NSD2*) and *NSD1*. *WHSC1* was associated with CPO, while *NSD1* was associated with CLO.
6. Basic helix-loop-helix TF: *MYCN*. *MYCN* mainly contributed to CLO and CLP, but it also showed a weak association with CPO.
7. MAF bZip TF: *MAFB*. *MAFB* mainly contributed to CLO and CLP.

We suggested that these groups of TFs and their target genes function in a coordinated manner to direct palate and lip tissue specialization during embryonic development and intermittently in response to external signals. Besides these TFs, Carlson JC *et al*. also identified validated TFs *PAX7* (1p36.13) and *GRHL3* (1p36.11) were also associated with CLP vs. CP group, and transcriptional corepressor *TLE1* (9q21.32) was also associated with CLP vs. CP group [28].

Using gene network analysis, we found that a total of 243 genes were either enriched by interaction with the genetic factors or in the same ontology as the candidate genes for NSOFC with different biological functions. Although we confirmed only five genes that surpassed the genome-wide statistical significance in the final meta-analysis of the discovery and replications cohorts, we could not exclude the rest of the genes as candidates with NSOFC for the following reasons: (1) the power might be insufficient to archive the genome-wide statistical significance level due to the limited number of samples in this study, (2) the genetic contributions responsible for NSOFC were missed in the GWAS because of the limited covered regions in the genome of the chip design and (3) their pathogenesis roles for NSOFC might be at the functional modulation level, not at the genetic variation effect, through interaction with the genetic factors.

Strikingly, the directions of the associated SNPs in the *IRF6* gene with CPO and CLO were opposite, suggesting that these two subtypes have different pathogenesis with *IRF6*, probably by regulating its expression via the associated SNPs. For example, for one of the leading SNP rs72741048 in this region, OR__T_ for CPO was 1.314 suggesting its risk effect, while the OR__T_ for CLO was 0.575 suggesting its protective effect. *IRF6* belongs to a family of transcription activators that share a highly conserved, helix-turn-helix, DNA-binding domain. The AP-2α enhancer was previously reported to be associated with CLO through binding with the rs642961 site in the intron of *IRF6* to regulate its expression [33]. It is likely that the expression of *IRF6* is precisely controlled by the coordination of multiple regulatory elements located in the associated SNPs in the gene region [34, 35]. The phenotypes of *IRF6* mouse models further suggest that the gene dosage balance might be critical for palate and lip development. *IRF6*-null mouse embryos showed oral cavity adhesion [36], implying that the normal palate development was impaired without *IRF6*. However, approximately 22.0% of *IRF6* transgenic mice exhibit an absence of calvaria, but they retain normal palatal shelf fusion. In contrast, 2.7% of these mice had a cleft lip [37], which supports the high expression level of *IRF6* as a risk for CLO. Further investigations are needed to dissect the pathogenesis of the dosage effect of *IRF6* for the CPO and CLO.

We searched the GWAS Catalog and found five studies based on CPO-related GWAS [12, 15, 38–40]. These studies did not discover *IRF6* locus in CPO perhaps because of 1) a small sample size in the discovery stage and 2) a large degree of population mixture in the discovery stage. From our results, we can see that the power of *IRF6* locus in CPO is much lower than that in CLO, so it may be beyond the cutoff threshold of power for GWAS discovery in the small and mixed discovery samples used in the preceding CPO GWAS. However, at least three studies suggested some clues to the association of *IRF6* locus with CPO or suggested the opposite OR directions in the SNPs of *IRF6* locus in CLO versus CPO. In 2008, Rahimov F. et al. reported that the rs2235371 and rs642961 haplotypes located in the *IRF6* region are associated with CL/P. They further validated them in CLO and CPO. These results suggest opposite OR directions in CLO versus CPO in most of the study populations (Norway, Denmark, EUROCRAN, Europe and Philippines) [33]. In a 2016 Chinese GWAS of nonsyndromic CLP in the discovery stage, the authors identified that *IRF6* locus was associated with CLP. Further, they replicated rs861020 in the intron of *IRF6* in the replication cohorts of CLP, CLO and CPO. They found that the A allele of this SNP is significantly associated with CLP versus CLO (OR = 0.72, *P* = 2.05 × 10^−9^) or CLP versus CPO (OR = 1.51, *P* = 8.69 × 10^−11^), suggesting opposite OR directions in these two diseases [24]. Another study reported that rs2235375 in the intron of *IRF6* was associated with CPO but not with CLO and CLP in a South Indian population [41].

After the *IRF6* gene locus, the *PAX9* locus in 14q13.3 was the second strongest associated locus for CPO. In the *PAX9* locus, the associated SNPs are located in the intron of the *SLA25A* gene, ~100kb downstream of *PAX9*. A previous study indicated that mice with a deleted *SLA25A* gene presented obvious phenotypes of CPO via reduction of *PAX9* expression [42]. *PAX9* encodes a key TF that was reported to play a role in organs derived from neural crest mesenchyme [43]. *PAX9* was required for secondary palate development in mice [44–46]. The absence of teeth and the formation of a cleft secondary palate in *PAX9*-deficient mice have been reported [45]. Furthermore, mutations in *PAX9* can cause tooth agenesis in humans [47]. Therefore, it is likely that the CPO associated genetic variants decreased the *SLA25A* expression and further down-regulated *PAX9* expression to associate with the disease. This needs to be addressed in future work.

In a recent GWAS, Yu et al. found in a Chinese population 14 loci based on the nonsyndromic CLP in the GWAS discovery stage, but not CPO or CLO [24]. In the current study, we used CPO and CLO in the discovery stage to find new loci for each group. This is very different at the beginning of the study design from Yu et al.’s study. Because the phenotype CLP is different from CPO or CLO, the genetic factors of these phenotypes may be different; that is the question we raised in this paper. We found that the main effect loci, such as *IRF6*, *MSX1*, *VAX1* and *MAFB*, are shared by CLP and CLO. We think the difference between CPO, CLO and CLP are caused by minor effect multi-genetic factors rather than heterogeneity among populations.

In summary, our study advanced current understanding of the genetic architectures of CPO, CLO and CLP. These findings defined the NSOFC subtypes using genetic factors and their functional ontologies. They also provide a clue to improving a diagnosis and treatment of these conditions in the future. However, the current understanding of the biology of these processes in humans remains largely unknown, and it is expected to be complicated. Further functional studies of the genes for NSOFC identified in this study should be conducted to promote drug development and novel therapeutic approaches to treat the disorder.

## Methods

### Subjects

All of the CPO, CLO and CLP cases were nonsyndromic. The CPO cases included the complete cleft palate (the hard and soft cleft palate) and the soft cleft palate. The diagnoses, which were made by professional maxillofacial doctors before surgery, were based on a series of tests, including electrocardiogram, radiography, biochemical test, physical examination, speech evaluation, ultrasonic test and genetic counseling as necessary. Only those controls who had no family history of congenital disease were included in this study. All of these evaluations were done by at least three doctors, including a surgeon, a speech clinician and a geneticist.

A three-stage GWAS for CPO and CLO was conducted, with further replications for CLP. The discovery stage involved 935 CPO patients, 948 CLO patients and 5,050 control individuals (cohort 1). The first replication study was performed among an additional 724 unrelated CPO cases, 781 CLO cases and 3,265 controls (cohort 2). The second replication study was performed among an additional 417 unrelated CPO cases, 492 CLO cases and 1,832 controls (replication cohort: Northern Han Chinese, cohort 3). The first replication for CLP involved 2,270 CLP cases and 3,265 controls (replication cohort: Southern Han Chinese, cohort 4). The second replication study for CLP was performed among an additional 427 unrelated CLP cases and 1,832 controls (replication cohort: Northern Han Chinese, cohort 5). The same controls were used for CPO, CLO and CLP. No obvious geographic areas or genetic differences occurred in this study. In the discovery stage, the CPO and CLO samples were collected by the same team in the same hospital of Southern Han Chinese people (West China Hospital of Stomatology, Chengdu). The control samples were also collected in the same city of Southern Han Chinese people (Sichuan Provincial People’s Hospital, Chengdu). The same controls were used for CPO and CLO. For the CPO, CLO and control samples, we used the same chip for genotyping (HumanOmniZhongHua-8 BeadChip, Illumina). In the replication studies, we enrolled the CPO, CLO, CLP and control samples in the same hospitals (CPO, CLO, CLP and controls were enrolled together).

### Ethics statement

The study was approved by the institutional ethics committee of West China Hospital of Stomatology of Sichuan University and Sichuan Provincial People’s Hospital and was conducted according to the Declaration of Helsinki principles[48]. All controls were healthy individuals without NSOFC or family history of NSOFC (including first-, second- and third-degree relatives). Written informed consent was obtained from all the participants or their guardians. Approximately 4 ml of venous blood was collected from each participant and placed in a tube containing ethylenediaminetetraacetic acid (EDTA) as the anticoagulant. Genomic DNA was extracted from peripheral blood lymphocytes using the standard sodium dodecylsulfate (SDS)–proteinase K-phenol/chloroform method.

### Genotyping and quality control in the GWAS

The discovery cohort DNA samples were genotyped by Jinneng Biotech (Shanghai, China) using HumanOmniZhongHua-8 BeadChip (Illumina), according to the manufacturer’s protocol, with a starting number of 900,015 SNPs. Any SNPs with call rates of less than 90% were removed from further analysis. SNPs located on the X and Y chromosomes, mitochondrial SNPs, and copy number variant probes were removed from further analysis, in keeping with current GWAS practices. After quality filtering and cleaning, 870,261 SNPs remained in the association analysis for CPO and CLO. Full details of the experimental workflow are provided in S2 Fig. Sex and PCs (PC1, PC2, PC3 and PC4) were adjusted as covariates in the logistic model in PLINK. Close relatives among the participants were calculated using genome-wide IBS/BD among all the samples using PLINK (–genome). We found that z0 was very close to 1 and z1 was very close to 0 in all the samples. Also, there are no related people (IBD pi-hat > 0.2) within these samples.

### Association analysis

After chip genotyping, PCA was performed for both CPO and CLO separately to remove samples with outlying samples from further analysis using the R statistical software package (see URLs). A total of 930 CPO cases, 945 CLO cases and 5,048 control individuals in the discovery cohort passed quality control for the GWAS discovery stage. Next, we examined potential genetic relatedness on the basis of pairwise identity by state for all of the successfully genotyped samples using PLINK version 1.9 software. The genomic inflation estimate (λGC) was calculated for variants with MAF > 1% using only directly genotyped SNPs using PLINK 1.9 (see URLs). Single-marker association analyses were performed using PLINK 1.9 adjusted for sex and MDS (PC1, PC2, PC3 and PC4) as covariates with SNPs showed missing values < 10%, MAF > 1% and HWE *P* > 10^−6^.

### Genotype imputation

Genotypes were converted to PLINK binary format, and SNPs with missing values > 10%, MAF < 1% and HWE *P* < 10^−6^ for phasing were excluded (see URLs). The clean data were then phased using SHAPEIT2 [49] (see URLs). After that, the dataset was imputed with 1000 Genomes phase 1 (version 3) of CHB (Han Chinese) and CHS (Southern Han Chinese) (hg19) with Minimac3 [50] (see URLs) with r^2^ > 0.6. The association analysis of the imputed dosage data was calculated using PLINK version 2.0 using sex and MDS (PC1, PC2, PC3 and PC4) as covariates.

### SNP selection for replication studies

SNPs showing an association with CPO and CLO exceeding *P* ≤ 9 × 10^−7^ in the GWAS discovery stage were included in the replication stage and analyzed in a similar manner to the discovery stage. In total, 48 SNPs were selected for replication analysis.

### Genotyping and quality control in the replication studies

Genotyping of the SNPs selected for the replication studies were conducted using the Sequenom MassARRAY system genotyping as previously described [27]. The association analysis of the replication genotype data was conducted using PLINK 1.9 adjusted for sex.

### Meta-analysis

PLINK 1.9 was used to perform combined meta-analyses of the GWAS discovery and replication data sets for CPO and CLO. The two CLP replication datasets were also combined using the PLINK 1.9 meta-analysis method.

### Manhattan plots, QQ plots and LocusZoom plots

Manhattan plots were generated using QQ, and Manhattan plots for the GWAS Data R package were generated using SNPs with imputed *P* values less than 0.05 (see URLs). QQ plots were generated using QQ, and Manhattan plots for the GWAS Data R package were generated using all the direct genotyped SNPs (see URLs). LocusZoom plots were generated online from LocusZoom (hg19 November 2014 ASN population) using SNPs with *P* values less than 0.05 (see URLs).

### Epigenetic annotation

Epigenomic annotation of genetic variants for 31 tissues was performed using the Roadmap Epigenome Browser, which was based on the WashU (Washington University) Epigenome Browser and integrates data from both the NIH (National Institutes of Health) Roadmap Epigenomics Consortium and ENCODE (Encyclopedia of DNA Elements) in a visualization [51] (see URLs).

### HI-C data browser

Hi-C contact matrices were visualized as heatmaps using the 3D Genome Browser [52] (see URLs). The TAD dataset of normal human epidermal keratinocytes and juvenile foreskin primary cell Hi-C data were used. The hg19 SNP region of the regional association maps was used as the input region.

### Gene expression

Human tissue samples were obtained from CPO and CLO patients during surgical cleft repair. Tissues were collected from the rim of the uvula of CPO patients and from the edge of the upper lip cleft of CLO patients. According to the principles of interdisciplinary team care for cleft lip and palate, the patient’s age at operation is usually between three and six months for CLO patients and between one and two years for CPO patients. We collected 211 tissue samples (105 palate tissues, 106 lip tissues) for gene expression analysis. Because the patients were young at the time of surgery and the lesion range is relatively limited, the sample size we collected was very small (about 3 mm*4 mm*2 mm) for each patient. In almost all cases, only one tissue sample was collected from one patient. Written informed consent was obtained from all guardians on behalf of the patients. All tissues were stored in liquid nitrogen immediately after incision and then transferred to –80°C for storage.

### Confocal immunohistochemistry

Human tissue samples were fixed in 4% paraformaldehyde in phosphate-buffered saline (PBS) for two hours, embedded in optimal cutting temperature compound and processed for sectioning (12 μm). The tissue sections were then blocked with 5% normal donkey serum for one hour (Jackson ImmunoResearch). The sections were then incubated with the primary anti-*IRF6* monoclonal mouse antibody (1:500, R&D Systems Antibodies) in the blocking buffer at 4°C overnight. Alexa488-conjugated goat anti-mouse secondary antibody was incubated for two hours at room temperature (diluted 1:500; Molecular Probes). Nuclei were visualized by counterstaining with 4′-6-diamidino-2-phenylindole (DAPI; Sigma-Aldrich) in the secondary antibody buffer. After PBS wash and mountant to cure overnight at room temperature, images were obtained using an inverted confocal microscope (Leica Microsystems CMS).

### Real-time PCR

RNA from human tissue was extracted with TRIzol (Invitrogen) treated with DNase I (RNase-free) (Ambion, Life Technologies) according to the protocol. The RNA concentration was determined using a NanoDrop ND-1000 spectrophotometer. Reverse transcription was performed using a commercial kit (Invitrogen). Real-time PCR was performed with an ABI7500 fast system according to the manufacturer’s instructions (Applied Biosystems). For each sample, the real-time PCR experiments were repeated three to four times. TaqMan probes (Life Technologies) were used to quantify *IRF6* and *GAPDH* expression. Analysis of relative *IRF6* gene expression data was done using the 2^-ΔΔ*C*^T method [53].

### Candidate gene network and ontology analysis

#### Candidate gene analysis

A series of genes for CPO, CLO and CLP were used to perform candidate analyses: (1) genes reported in review references; (2) GWAS catalog genes (*P* < 9.0 × 10^−6^), which were included in the database; (3) Human Gene Mutation Database (HGMD) genes; (4) phenolyzer seed genes; and (5) human phenotype ontology (HPO) genes. As CL/P combines CPO, CLO and CLP phenotypes and was typically studied as a whole object, we also analyzed its candidate genes as CL/P to provide additional information for understanding the similarities and differences among orofacial cleft subtypes. For the above search sources, only a clear phenotype or functional illustration of “cleft lip only,” “cleft palate only,” “cleft lip and palate” and “cleft lip with or without palate” were kept as “candidate genes,” while other related phenotypes or syndrome-related genes were not included.

#### Gene network analysis

Gene network analysis was conducted using the Cytoscape GeneMANIA app, which can import interaction networks from public databases from our candidate genes with their annotations and putative functions. Network visualization was processed by Cytoscape 3.3.

#### Gene ontology and network analysis

Candidate gene lists were functionally noted by DAVID to produce ontology categories, terms, gene sets and *P* values. An ontology category false discovery rate (FDR) less than 0.05 was used for ontology network analysis. Ontology network analysis was produced by the Cytoscape EnrichmentMap app using the DAVID output ontology categories. Network visualization was processed by Cytoscape 3.3 (S6 Fig).

#### Gene ontology clustering

Gene ontology clustering was produced using Cluster 3.0 with -log10^FDR^ values. An FDR cutoff of less than 0.001 was used for clustering to provide the most statistically significant information (S7 Fig).

The final candidate genes are listed in S5 Table. In total, 243 genes were enriched, including 98 CPO genes, 56 CLO genes, 68 CLP genes and 57 CL/P genes.

#### SNP selection for replication by candidate gene analysis

Forty-four SNPs in the 243 candidate gene regions (*P* ≤ 2.06 × 10^−4^) in the GWAS discovery stage were included in the replication studies in Southern Han Chinese and Northern Han Chinese cohorts.

### URLs

GWAS catalog (the NHGRI-EBI Catalog of published genome-wide association studies): http://www.ebi.ac.uk/gwas/;

ANNOVAR: http://annovar.openbioinformatics.org/en/latest/;

HI-C data browser: http://promoter.bx.psu.edu/hi-c/;

Minimac3: http://genome.sph.umich.edu/wiki/Minimac3_Imputation_Cookbook;

PLINK 1.9: http://zzz.bwh.harvard.edu/plink/;

PLINK 2.0: https://www.cog-genomics.org/plink/2.0/;

R statistical program package: https://www.r-project.org/;

Q-Q and Manhattan Plots for GWAS Data R package: https://cran.r-project.org/web/packages/qqman/index.html;

SHAPEIT2: https://mathgen.stats.ox.ac.uk/genetics_software/shapeit/shapeit.html;

Chromatin state annotations for selected Roadmap Epigenomic tissues: http://epigenomegateway.wustl.edu/browser/roadmap/;

LocusZoom: http://locuszoom.org/;

EnrichmentMap: http://apps.cytoscape.org/apps/enrichmentmap;

GeneMANIA: http://apps.cytoscape.org/apps/genemania;

GTEx Portal: https://gtexportal.org/home/;

HaploReg database v3: http://archive.broadinstitute.org/mammals/haploreg/haploreg_v3.php;

Human Phenotype Ontology (HPO): http://human-phenotype-ontology.github.io/;

MGI (Mouse Genome Informatics): http://www.informatics.jax.org/;

Online Mendelian Inheritance in Man (OMIM): https://www.omim.org/;

Phenolyzer: http://phenolyzer.wglab.org/;

The Human Gene Mutation Database (HGMD): http://www.hgmd.cf.ac.uk/ac/index.php.

## Supporting information

S1 Data The association results of CPO and CLO adjusted for sex and PCs (PC1, PC2, PC3 and PC4) analyzed by PLINK with P values less than e-5 in the discovery stage (935 CPO patients, 948 CLO patients and 5,050 control individuals). CHR: Chromosome; SNP: Physical position in Chromosome; BP: Physical position (base-pair); A1: Minor allele name (based on whole sample); TEST: Type of test; OR: Estimated odds ratio (for A1, i.e. A2 is reference); SE: Standard error; L95: Lower bound of 95% confidence interval for odds ratio; U95: Upper bound of 95% confidence interval for odds ratio; STAT: Coefficient t-statistic; P: Asymptotic p-value for t-statistic.
(XLSX)

S1 Fig The phenotype of the diseases. Bilateral cleft lip only (CLO), Cleft palate only (CPO),Cleft lip with cleft palate (CLP).The pictures were authorized for use in this paper.
(PDF)

S2 Fig Experimental workflow of this study. (PDF)

S3 Fig Gross population stratification. Principal component analysis (PCA) for the GWAS samples: 930 CPO patients (BLACK), 945 CLO patients (RED) and 5,048 control individuals (GREEN). Although there are a bit structure among the controls in C2 vs. C3 and C2 vs. C4, the values are very low.
(PDF)

S4 Fig QQ plots of the association results of CPO and CLO. (PDF)

S5 Fig The regional information of the IRF6 LD. Most of the associated SNPs in theIRF6 region were located in the 5’UTR and intronic regions, containing enrichment signals of active transcription start site (TSS), transcription, enhancers and ChIP-seq chromatin profiling signals.
(PDF)

S6 Fig Gene functional maps of NSOFC. (a)The sketch contribution map of statistically significant transcription factors validated for CPO or CLO. (b-h) Gene networks analyzed by GeneMANIA. The queried genes were shown in the pink nodes. The predicted genes were shown in the light green nodes. The blue lines between nodes indicate a physical interaction; green lines indicated genetic interactions; light red lines indicated co-localization; and brown lines indicated predictions. The candidate genes were from published references, GWAS catalog, HGMD and HPO. (B) CPO genes. (C) CLO genes. (D) CLP genes. (E) Candidate CPO genes. (F) Candidate CLO genes. (G) Candidate CLP genes. (H) Candidate CL/P genes. (i-l) Network of candidate gene ontologies by DAVID and EnrichmentMap. The node area indicates the FDR q-value. (i) Candidate CPO genes. (j) Candidate CLO genes. (K) Candidate CLP genes. (L) Candidate CL/P genes.
(JPG)

S7 Fig Clustering analysis of functional ontology FDR q-values of NSOFC Candidate genes. The FDR q-value cutoff was 0.001 for this cluster. Blank cells indicate that no ontology was found in that GO term in the corresponding disease.
(PDF)

S1 Table The 16 previously reported loci associated with CL/P that also showed a marginal association (*P*<0.05) in the present study with CPO or CLO. (PDF)

S2 Table Discovery and replication results of the rest SNPs selected for validation in the typical GWAS. (PDF)

S3 Table The association of 537 markers (*P*< 9×10^−7^) for CPO or CLO in the *IRF6* locus LD in chromosome 1q32.2 in the discovery stage. (PDF)

S4 Table The regulatory elements in the HaploReg database in the *IRF6* region. (PDF)

S5 Table Candidate genes for NSOFC. (PDF)

S6 Table Discovery and replication results of the rest SNPs selected for validation by gene network and ontology analysis. (PDF)
