# Supplementary figures and images for: Genetic factors define CPO and CLO subtypes of nonsyndromicorofacial cleft
Source: PLoS Genet. 2019 Oct 14;15(10):e1008357. doi: 10.1371/journal.pgen.1008357 (PMC6812857; doi:10.1371/journal.pgen.1008357)

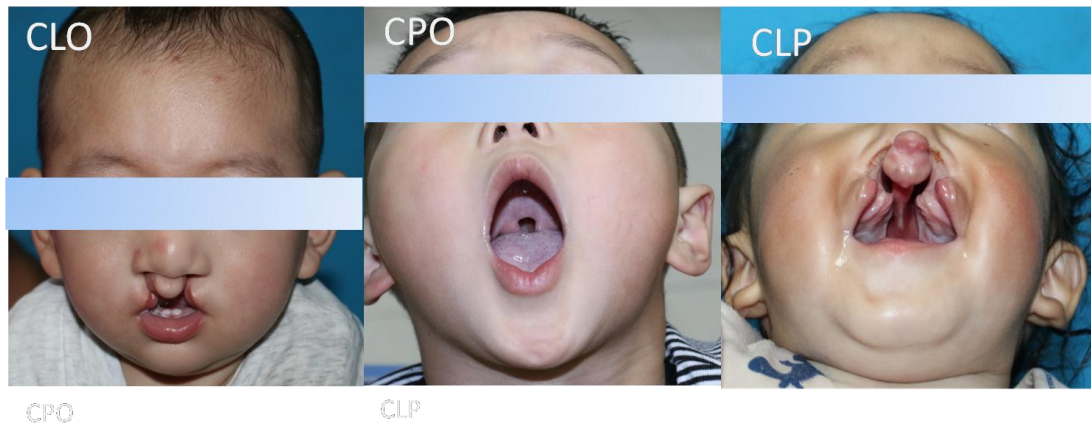

Supplement: S1 Fig — Bilateral cleft lip only (CLO), Cleft palate only (CPO),Cleft lip with cleft palate (CLP).The pictures were authorized for use in this paper. (PDF) [file pgen.1008357.s002.pdf]

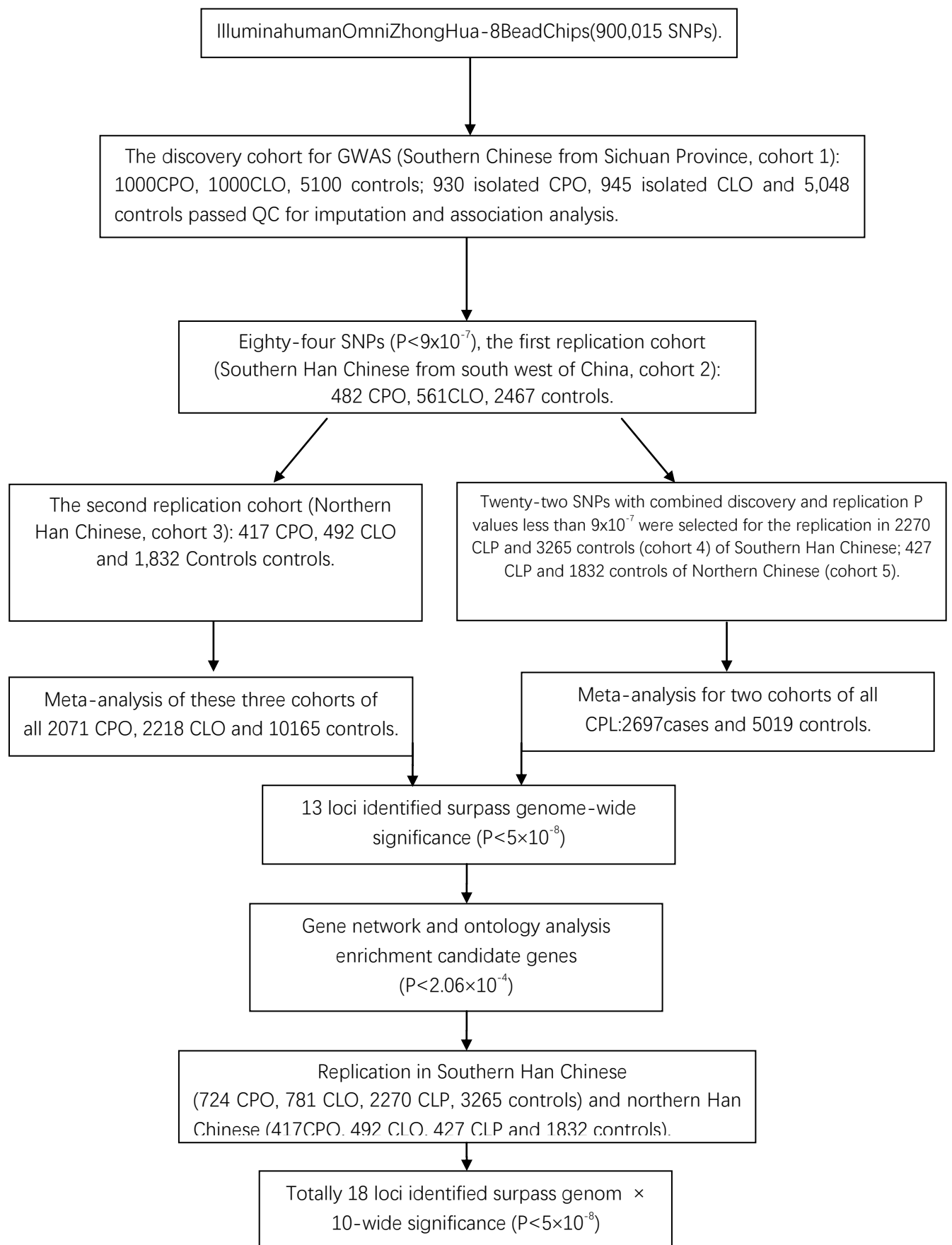

Supplement: S2 Fig — (PDF) [file pgen.1008357.s003.pdf]

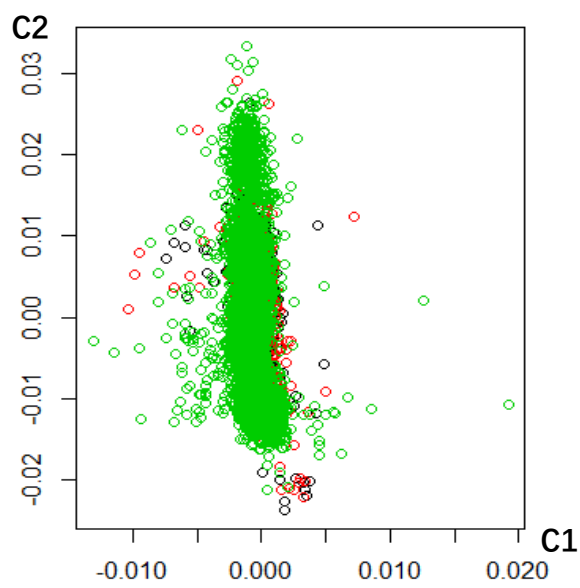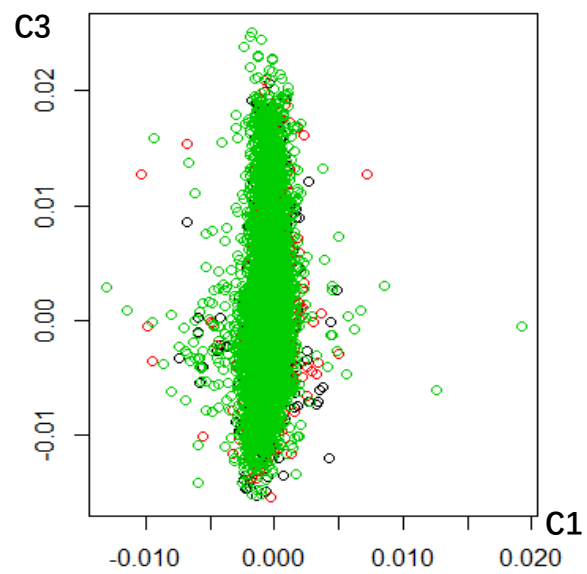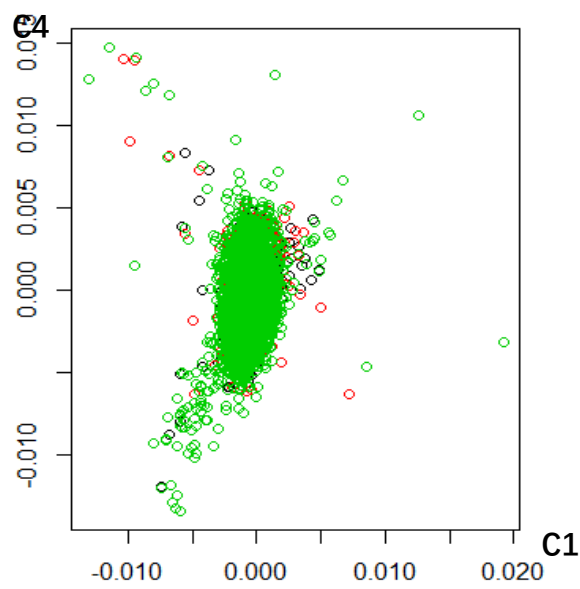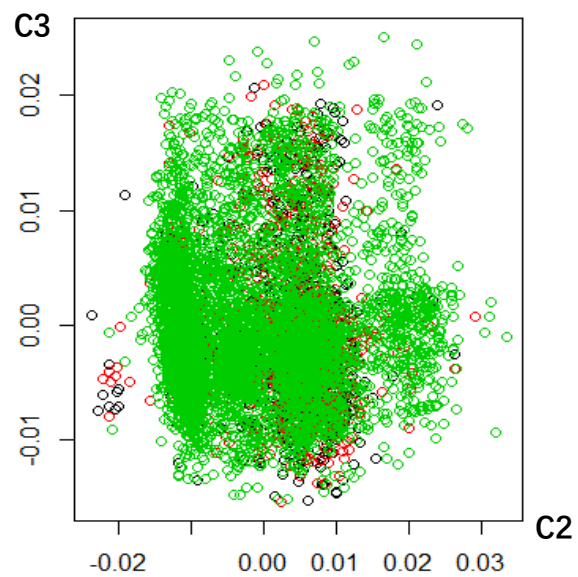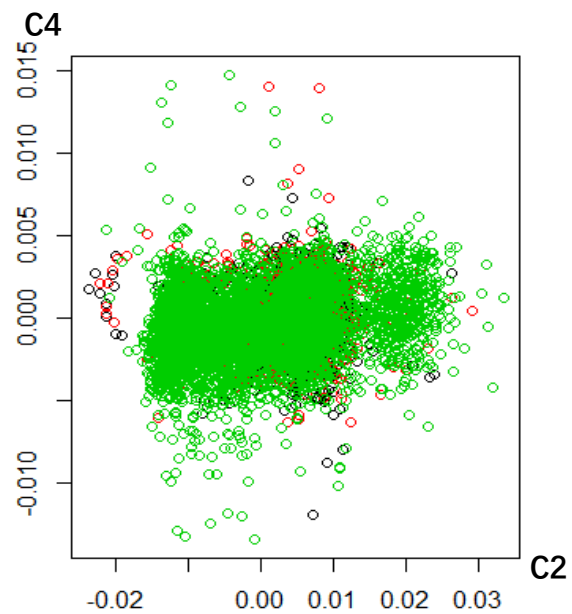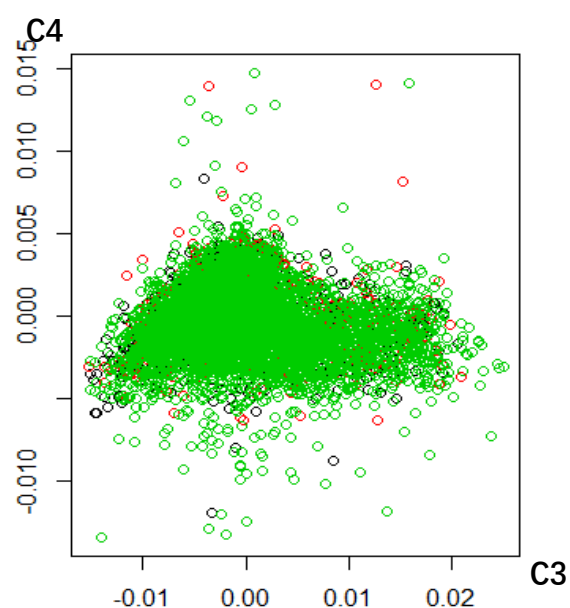

Supplement: S3 Fig — Principal component analysis (PCA) for the GWAS samples: 930 CPO patients (BLACK), 945 CLO patients (RED) and 5,048 control individuals (GREEN). Although there are a bit structure among the controls in C2 vs. C3 and C2 vs. C4, the values are very low. (PDF) [file pgen.1008357.s004.pdf]

CPO

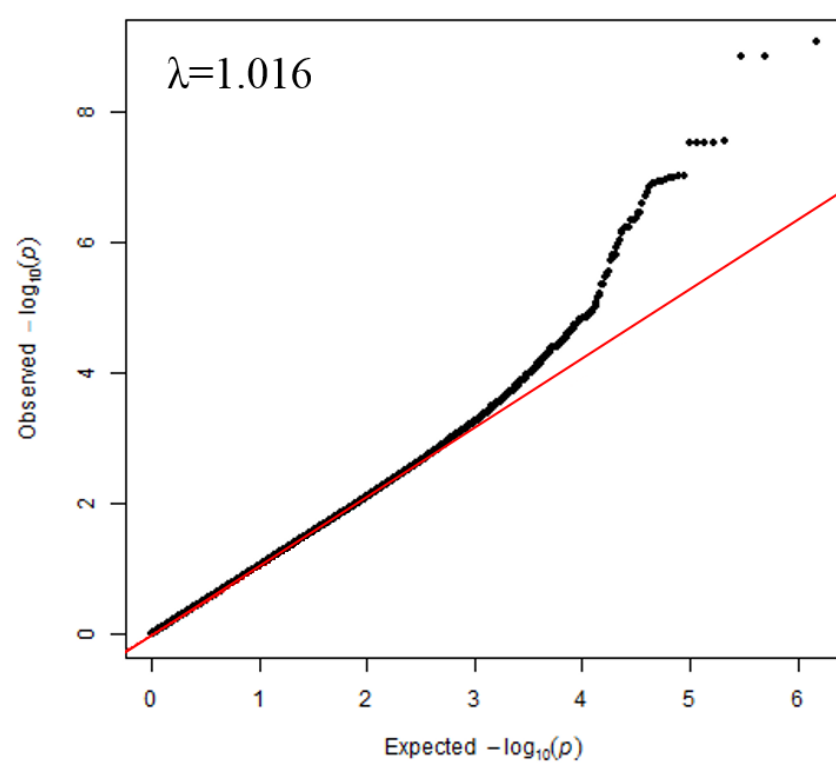

CLO

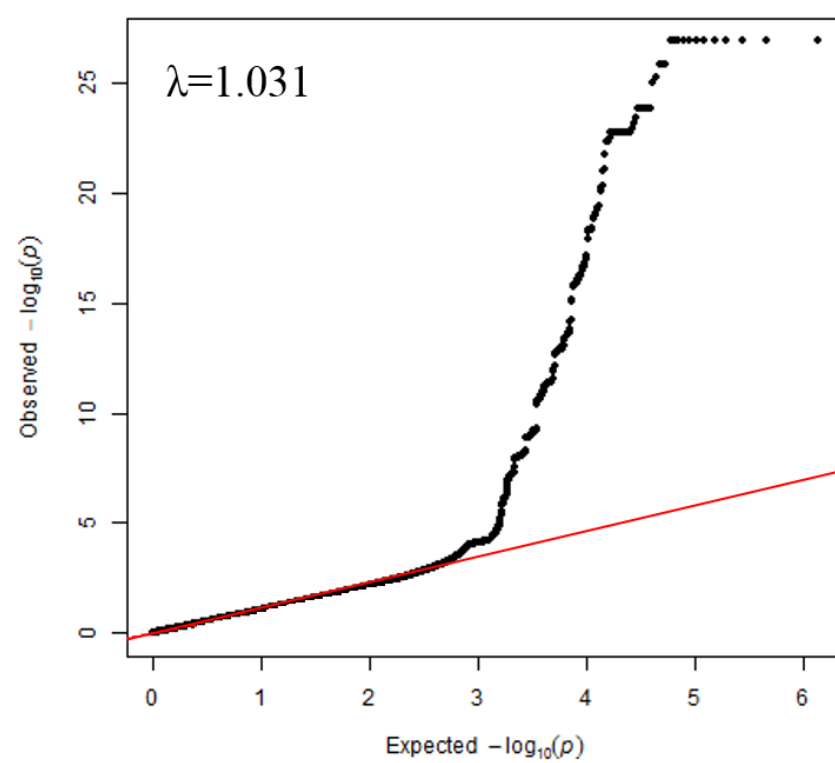

Supplement: S4 Fig — (PDF) [file pgen.1008357.s005.pdf]

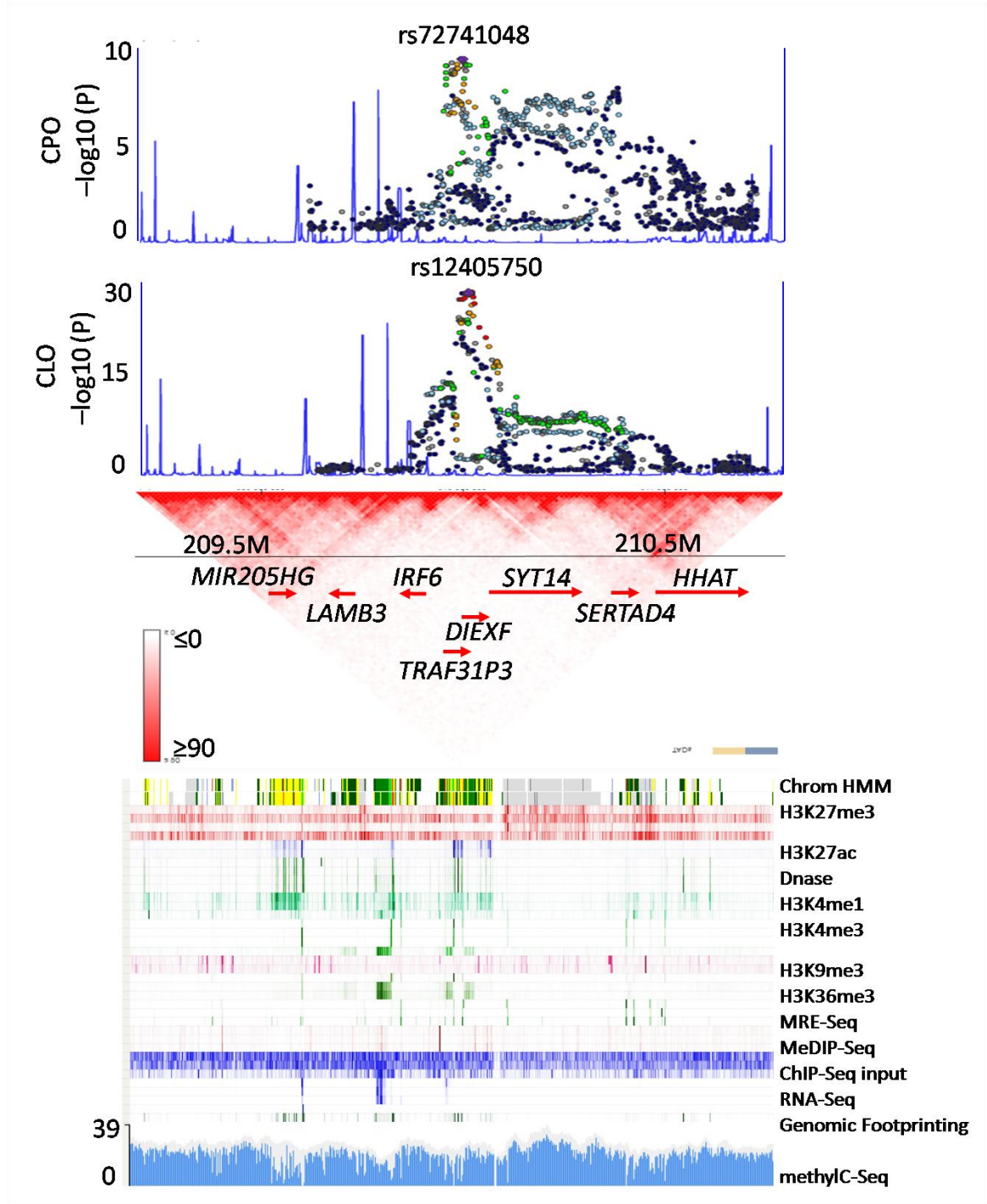

Supplement: S5 Fig — Most of the associated SNPs in theIRF6 region were located in the 5’UTR and intronic regions, containing enrichment signals of active transcription start site (TSS), transcription, enhancers and ChIP-seq chromatin profiling signals. (PDF) [file pgen.1008357.s006.pdf]

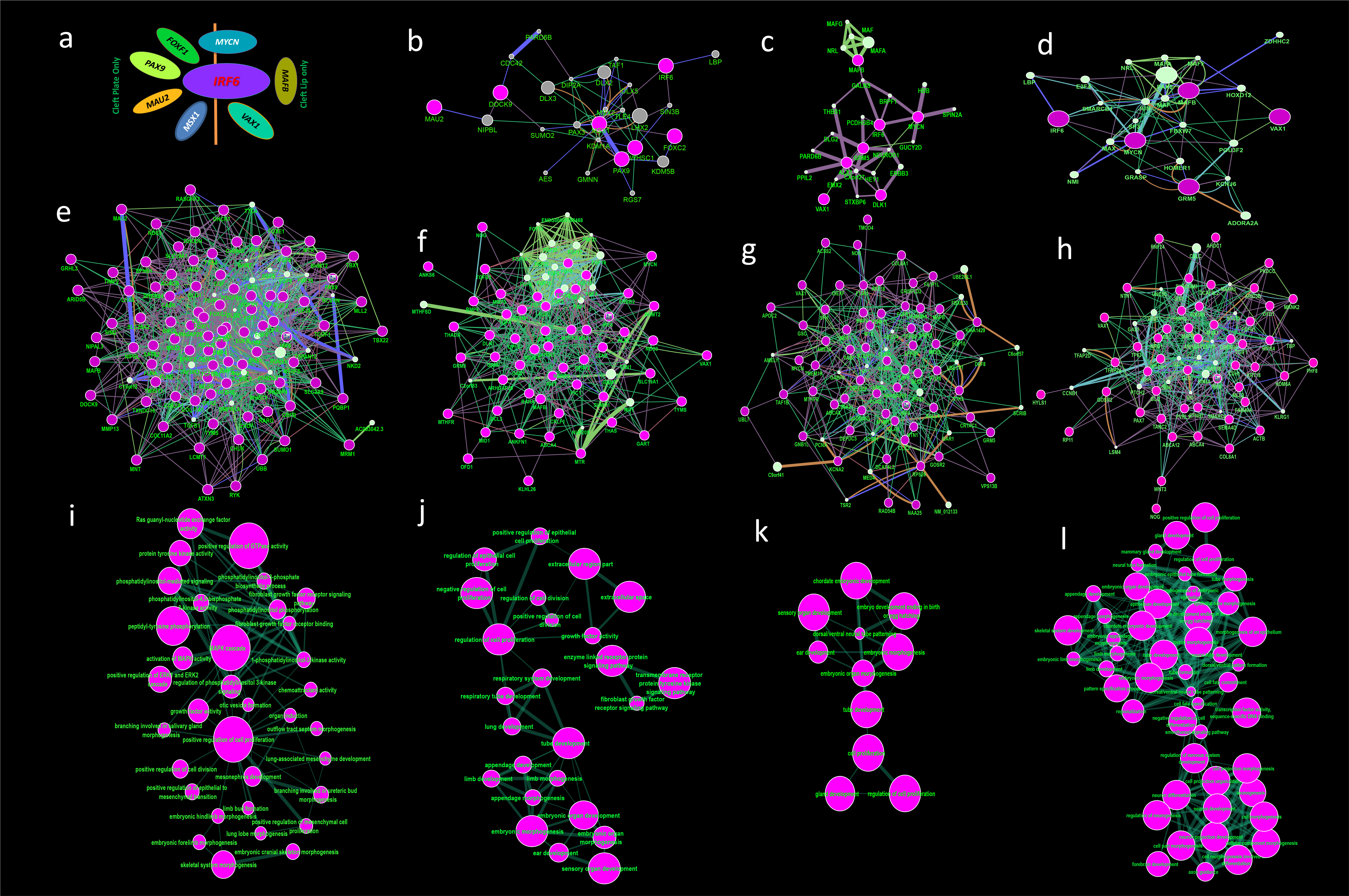

Supplement: S6 Fig — (a)The sketch contribution map of statistically significant transcription factors validated for CPO or CLO. (b-h) Gene networks analyzed by GeneMANIA. The queried genes were shown in the pink nodes. The predicted genes were shown in the light green nodes. The blue lines between nodes indicate a physical interaction; green lines indicated genetic interactions; light red lines indicated co-localization; and brown lines indicated predictions. The candidate genes were from published references, GWAS catalog, HGMD and HPO. (B) CPO genes. (C) CLO genes. (D) CLP genes. (E) Candidate CPO genes. (F) Candidate CLO genes. (G) Candidate CLP genes. (H) Candidate CL/P genes. (i-l) Network of candidate gene ontologies by DAVID and EnrichmentMap. The node area indicates the FDR q-value. (i) Candidate CPO genes. (j) Candidate CLO genes. (K) Candidate CLP genes. (L) Candidate CL/P genes. (JPG) [file pgen.1008357.s007.jpg]

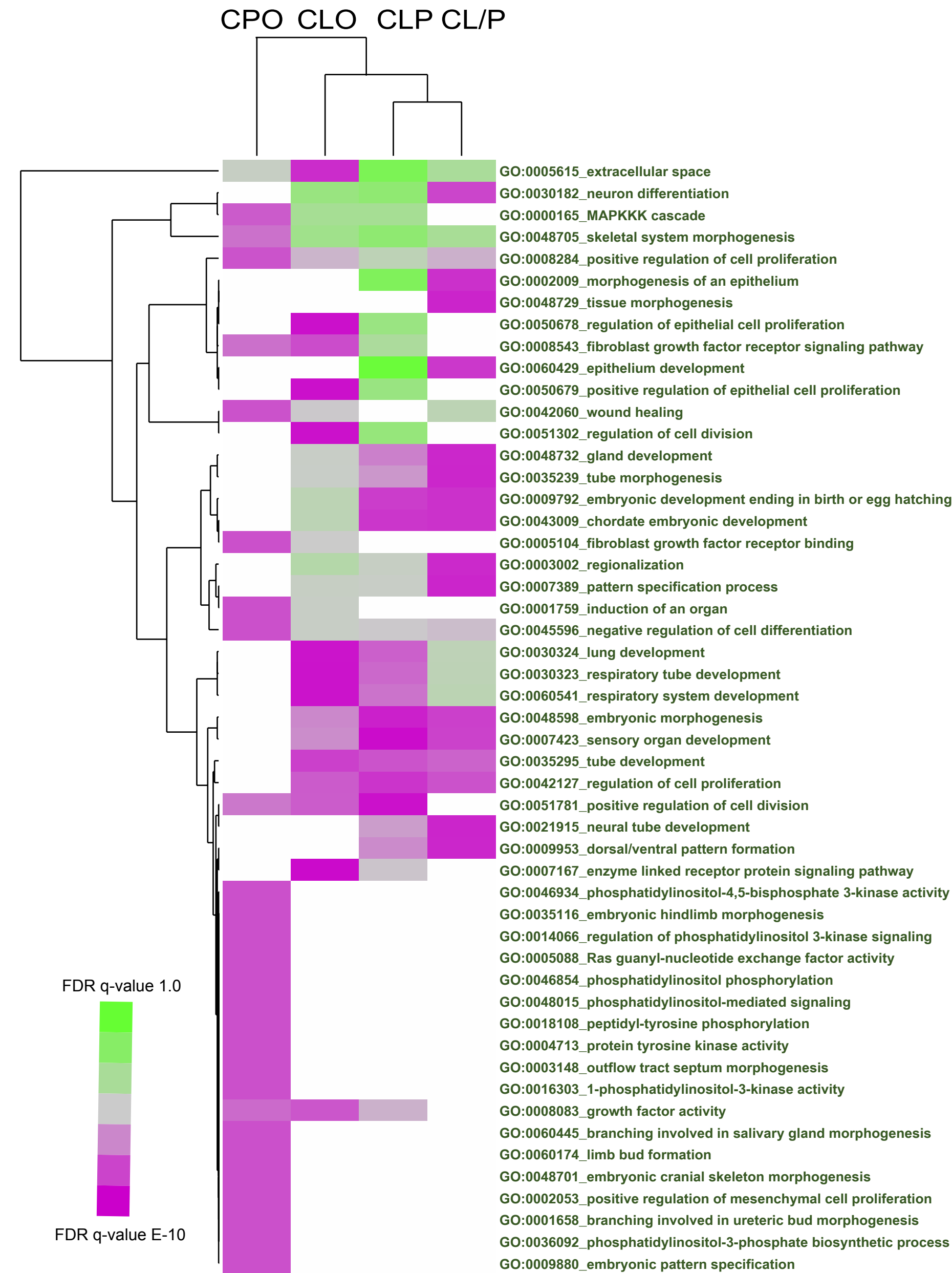

Supplement: S7 Fig — The FDR q-value cutoff was 0.001 for this cluster. Blank cells indicate that no ontology was found in that GO term in the corresponding disease. (PDF) [file pgen.1008357.s008.pdf]
