# Supplementary material for: Genetic factors define CPO and CLO subtypes of nonsyndromicorofacial cleft
Source: PLoS Genet. 2019 Oct 14;15(10):e1008357. doi: 10.1371/journal.pgen.1008357 (PMC6812857; doi:10.1371/journal.pgen.1008357)
Supplement: S1 Table — (PDF) [file pgen.1008357.s009.pdf]

Supplementary Table 1. The 22 previously reported loci associated with CL/P, CPO or CLP that also showed a marginal association (*P*<0.05) in the present study with CPO or CLO.

CHR	SNP	GENE	Cytoband	BP (hg19)	A1	A2	F_A-CPO	F_U-CPO	P-CPO	OR-CPO	F_A-CLO	F_U-CLO	P-CLO	OR-CLO	PUBMED ID	Reported Phenotype
1	rs6704504	*PAX7*	1p36.13	18926589	C	T	0.086	0.098	0.126	0.866	0.079	0.098	0.017	0.795	31172578	CLP
1	rs9439716	*PAX7*	1p36.13	18978263	C	A	0.351	0.320	0.015	1.147	0.337	0.320	0.164	1.081	31172578	CLP
1	rs61776460	*GRHL3*	1p36.11	24879226	G	A	0.063	0.048	9.31 ×10-3	1.343	0.046	0.048	0.793	0.968	31172578	CPO
1	rs560426	*ABCA4*	1p22.1	94553438	C	T	0.288	0.300	0.327	0.944	0.339	0.300	0.002	1.193	22863734	CL/P
1	rs560426	*ARHGAP29*	1p22.1-21.3	94553438	C	T	0.288	0.300	0.327	0.944	0.339	0.300	1.79 ×10-3	1.193	31172578	CLP
1	rs66515264	*ARHGAP29*	1p22.1-21.3	94558110	T	G	0.054	0.056	0.668	0.951	0.074	0.056	4.83 ×10-3	1.342	31172578	CLP
2	rs287980	*YWHAQ;TAF1B*	2p25.1	9971366	G	A	0.265	0.271	0.605	0.969	0.248	0.271	0.049	0.887	28232668	CLP
2	rs287982	*YWHAQ;TAF1B*	2p25.1	9972442	C	T	0.265	0.271	0.605	0.969	0.248	0.271	0.049	0.887	28232668	CLP
3	rs7632427	*EPHA3*	3p11.1	89534377	C	T	0.182	0.176	0.605	1.037	0.150	0.176	0.009	0.826	22863734	CL/P
3	rs813218	*CLO8A1, CMSS1*	3q12.1	99592596	T	C	0.332	0.322	0.423	1.047	0.289	0.322	0.009	0.859	22863734	CL/P
3	rs1474306	*C3orf58;LNCSRLR*	3q24	145361479	T	C	0.363	0.326	0.003	1.180	0.332	0.326	0.614	1.029	30452639	CL/P
3	rs6768171	*C3orf58;LNCSRLR*	3q24	145361918	T	G	0.363	0.326	0.003	1.180	0.333	0.326	0.594	1.031	30452639	CL/P
4	rs908822	*LOC285419*	4q28.1	124906257	T	C	0.090	0.069	2.34 ×10-3	1.340	0.076	0.069	0.258	1.121	28232668	CLP
5	rs604328	*UGT3A2*	5p13.2	36042219	C	T	0.066	0.048	3.15 ×10-3	1.390	0.060	0.048	0.0398	1.265	31172578	CPO
5	rs10462065	*NNT;FGF10*	5p12	44068846	A	C	0.222	0.210	0.269	1.074	0.234	0.210	0.028	1.148	28232668	CLP
8	rs13317	*BAG4,FGFR1*	8p11.23	38269514	C	T	0.301	0.337	4.21 ×10-3	0.848	0.313	0.337	0.060	0.899	28232668	CLP
8	rs12543318	*DCAF4L2, CTB-118P15.2*	8q21.3	88868340	A	C	0.360	0.380	0.123	0.918	0.327	0.380	3.79 ×10-5	0.794	22863734,28054174	CL/P
8	rs1034832	*DCAF4L2, CTB-118P15.2*	8q21.3	88918331	G	T	0.311	0.339	0.026	0.880	0.287	0.339	2.60 ×10-5	0.784	28232668	CLP
8	rs987525	*3' of AC068570.1*	8q24.21	129941196	A	T	0.085	0.066	5.53 ×10-3	1.314	0.080	0.066	0.030	1.241	20436469,19270707,19656524,22863734	CL/P
8	rs7017252	*3' of AC068570.1*	8q24.21	129950844	T	C	0.061	0.047	0.018	1.312	0.062	0.047	0.011	1.335	28232668	CLP
8	rs13274247	*LINC00976;LINC00977*	8q24.21	129981468	A	G	0.077	0.064	0.044	1.228	0.079	0.064	0.022	1.258	30452639	CL/P
8	rs744835	*LINC00976;LINC00977*	8q24.21	129982547	T	C	0.078	0.064	0.034	1.240	0.080	0.064	0.017	1.270	30452639	CL/P
9	rs7871395	*GADD45G*	9q22.2	92209587	T	C	0.274	0.262	0.340	1.059	0.293	0.262	0.009	1.165	28232668	CLP

12	rs705704	*RPS26*	12q13.2	56435412	A	G	0.222	0.236	0.214	0.924	0.262	0.236	0.021	1.151	28232668	CLP
12	rs2304269	*ZFC3H1, THAP2,TMEM19*	12q21.1	72080272	C	T	0.473	0.461	0.347	1.052	0.425	0.461	0.007	0.865	28232668	CLP
13	rs11620022	*3′ of RP11-501G7.1*	13q31.1	80625125	C	A	0.109	0.132	0.010	0.806	0.118	0.132	0.110	0.878	28054174,22863734,20023658	CL/P
13	rs60417080	*3′ of RP11-501G7.1*	13q31.1	80641209	A	T	0.109	0.133	8.83 ×10-3	0.803	0.120	0.133	0.159	0.893	28054174,22863734,20023658	CL/P
16	rs2283487	*3′ of RP11-462G12.2*	16p13.3	3969886	G	A	0.450	0.462	0.376	0.954	0.422	0.462	0.002	0.849	28232668	CLP
16	rs8049367	*3′ of RP11-462G12.2*	16p13.3	3980445	T	C	0.355	0.351	0.734	1.019	0.305	0.351	2.95 ×10-4	0.814	25775280	CL/P
17	rs4791774	*NTN1*	17p13.1	8932119	G	A	0.194	0.177	0.095	1.120	0.237	0.177	5.87 ×10-5	1.450	25775280	CL/P
17	rs1838105	*RP11-63A1.1,GOSR2*	17q21.32	45008935	A	G	0.388	0.388	0.946	1.004	0.421	0.388	0.009	1.150	28232668	CLP
17	rs227731	*NOG*	17q22	54773238	G	T	0.291	0.315	0.050	0.892	0.358	0.315	4.35 ×10-4	1.216	28054174,22863734,20023658	CL/P
