# Supplementary material for: Genetic factors define CPO and CLO subtypes of nonsyndromicorofacial cleft
Source: PLoS Genet. 2019 Oct 14;15(10):e1008357. doi: 10.1371/journal.pgen.1008357 (PMC6812857; doi:10.1371/journal.pgen.1008357)
Supplement: S2 Table — (PDF) [file pgen.1008357.s010.pdf]

Supplementary Table 2. Discovery and replication results of the rest SNPs selected for validation in the typical GWAS.

SNP	Loci	Affected Gene (a)	Alleles	Cohort	Discovery (c)			Replication CHS (d)			Replication CHB (e)			Combined (f)		
					MAF (b)	*P*	OR	MAF (b)	*P*	OR	MAF (b)	*P*	OR	*P*	OR	I (g)
rs994771	**4q23**	***ADH7***	G/A	CPO	0.3796/0.331	1.21 ×10-4	1.237	0.429/0.377	2.91 ×10-2	1.239	0.322/0.351	1.67 ×10-1	0.876	8.91 ×10-4	1.154	80.65
				CLO	0.4098/0.331	8.02 ×10-7	1.638	0.429/0.377	5.86 ×10-2	1.176	0.368/0.351	4.77 ×10-1	1.073	2.87 ×10-5	1.192	0
				CLP	.	.	.	0.429/0.377	1.09 ×10-3	1.161	0.384/0.351	8.39 ×10-2	1.152	2.13 ×10-4	1.159	0
rs7659550	**4q23**		G/T	CPO	0.3627/0.3029	1.87 ×10-3	1.140	0.39/0.337	1.96 ×10-2	1.262	0.343/0.349	7.65 ×10-1	0.972	7.06 ×10-3	1.124	46.82
				CLO	0.3847/0.3029	4.06 ×10-7	1.252	0.39/0.337	1.09 ×10-1	1.151	0.374/0.349	2.74 ×10-1	1.114	1.22 ×10-5	1.202	0
				CLP	.	.	.	0.39/0.337	7.82 ×10-3	1.132	0.405/0.349	3.51 ×10-3	1.268	1.74 ×10-4	1.164	31.61
rs147121504	**9q22.31**	***IARS***	T/C	CPO	0.06893/0.09854	1.21 ×10-4	0.677	0.071/0.1	3.93 ×10-2	0.686	0.071/0.114	3.93 ×10-2	0.686	1.59 ×10-6	0.681	0
				CLO	0.06599/0.09854	9.02 ×10-7	0.646	0.071/0.1	3.49 ×10-1	0.851	0.098/0.114	4.25 ×10-1	0.848	3.96 ×10-5	0.716	24.45
				CLP	.	.	.	0.071/0.1	1.92 ×10-2	0.645	0.098/0.114	4.25 ×10-1	0.848	3.02 ×10-5	0.681	27.66
rs7329196	**13q12.13**	***ATP8A2***	C/T	CPO	0.1909/0.15257	9.73 ×10-8	1.313	0.273/0.271	9.15 ×10-1	1.012	0.273/0.271	9.15 ×10-1	1.012	1.66 ×10-6	1.555	96.64
				CLO	0.1819/0.15257	2.51 ×10-7	1.179	0.305/0.271	1.26 ×10-1	1.180	0.321/0.271	3.62 ×10-2	1.326	2.89 ×10-6	1.699	92.25
				CLP	.	.	.	0.315/0.271	3.90 ×10-2	1.230	0.321/0.271	3.62 ×10-2	1.326	3.76 ×10-3	1.263	0
rs17106304	**14q24.1**	***ZFP36L1***	C/G	CPO	0.2128/0.2405	1.41 ×10-2	0.854	0.204/0.238	9.53 ×10-2	0.821	0.204/0.25	9.53 ×10-2	0.821	7.20 ×10-4	0.841	0
				CLO	0.1781/0.2405	1.76 ×10-8	0.684	0.202/0.238	7.98 ×10-2	0.809	0.319/0.25	1.04 ×10-2	1.407	2.31 ×10-5	0.796	91.36
				CLP	.	.	.	0.2/0.238	1.50 ×10-2	0.801	0.319/0.25	1.04 ×10-2	1.407	4.58 ×10-4	1.356	0
rs2236262	**14q24.1**		A/G	CPO	0.2303/0.2576	1.83 ×10-2	0.862	0.227/0.257	1.42 ×10-1	0.846	0.227/0.27	1.42 ×10-1	0.846	1.71 ×10-3	0.856	0
				CLO	0.1942/0.2576	2.37 ×10-8	0.695	0.212/0.257	3.25 ×10-2	0.776	0.333/0.27	2.31 ×10-2	1.349	6.49 ×10-6	0.789	90.14
				CLP	.	.	.	0.232/0.257	3.85 ×10-2	0.706	0.333/0.27	2.31 ×10-2	1.349	2.60 ×10-3	1.270	0
rs3742887	**14q24.1**		A/G	CPO	0.2128/0.2405	1.41 ×10-2	0.854	0.206/0.238	1.18 ×10-1	0.831	0.206/0.252	1.18 ×10-1	0.831	9.99 ×10-4	0.845	0
				CLO	0.1781/0.2405	1.76 ×10-8	0.684	0.198/0.238	5.32 ×10-2	0.790	0.32/0.252	1.21 ×10-2	1.400	1.40 ×10-5	0.791	91.16
				CLP	.	.	.	0.182/0.238	1.26 ×10-1	0.690	0.32/0.252	1.21 ×10-2	1.400	5.79 ×10-3	1.262	0
rs194778	**14q24.1**		A/T	CPO	0.2128/0.2404	1.47 ×10-2	0.854	0.211/0.241	1.72 ×10-1	0.845	0.211/0.253	1.72 ×10-1	0.845	1.89 ×10-3	0.851	0
				CLO	0.1781/0.2404	1.91 ×10-8	0.685	0.221/0.241	3.47 ×10-1	0.893	0.34/0.253	2.37 ×10-3	1.515	1.98 ×10-4	0.817	92.73
				CLP	.	.	.	0.211/0.241	1.01 ×10-4	0.625	0.34/0.253	2.37 ×10-3	1.515	3.72 ×10-1	0.922	95.7

rs448083	**19q13.42**	***LILRB2***	C/T	CPO	0.2872/0.2304	7.30 ×10-7	1.346	0.259/0.25	3.10 ×10-1	1.016	0.236/0.235	9.45 ×10-1	1.051	9.67 ×10-6	1.413	0
				CLO	0.2972/0.2304	4.93 ×10-8	1.412	0.251/0.25	3.20 ×10-1	1.003	0.247/0.235	2.45 ×10-1	1.120	7.96 ×10-6	1.355	0
				CLP	.	.	.	0.331/0.25	6.04 ×10-2	1.204	0.156/0.184	2.45 ×10-1	0.821	3.00 ×10-1	1.093	73.72
