# Supplementary material for: Genetic factors define CPO and CLO subtypes of nonsyndromicorofacial cleft
Source: PLoS Genet. 2019 Oct 14;15(10):e1008357. doi: 10.1371/journal.pgen.1008357 (PMC6812857; doi:10.1371/journal.pgen.1008357)
Supplement: S3 Table — (PDF) [file pgen.1008357.s011.pdf]

Supplementary Table 3. The association of 537 markers (*P*< 9×10^{-7}) for CPO or CLO in the *IRF6* locus LD in chromosome 1q32.2 in the discovery stage.

CHR	BP	A1_CPO	F_A_CPO	F_U_CPO	A2_CPO	P-A_CPO	OR-A_CPO	CI 95%_CPO	A1_CLO	F_A_CLO	F_U_CLO	A2_CLO	P_CLO	OR_CLO	CI 95%_CLO
1	209881045	G	0.296	0.2704	A	0.03239	1.134	1.011-1.273	G	0.1888	0.2704	A	1.28 ×10-12	0.6281	0.552-0.7148
1	209881373	T	0.2248	0.2354	G	0.3492	0.9421	0.8315-1.067	T	0.2999	0.2354	G	1.97 ×10-8	1.391	1.239-1.562
1	209882900	A	0.2248	0.2354	C	0.3492	0.9421	0.8315-1.067	A	0.2999	0.2354	C	1.97 ×10-8	1.391	1.239-1.562
1	209882906	T	0.2248	0.2354	A	0.3492	0.9421	0.8315-1.067	T	0.2999	0.2354	A	1.97 ×10-8	1.391	1.239-1.562
1	209883804	C	0.2248	0.2354	T	0.3492	0.9421	0.8315-1.067	C	0.2999	0.2354	T	1.97 ×10-8	1.391	1.239-1.562
1	209884387	G	0.2248	0.2354	A	0.3492	0.9421	0.8315-1.067	G	0.2999	0.2354	A	1.97 ×10-8	1.391	1.239-1.562
1	209884730	C	0.2248	0.2354	G	0.3492	0.9421	0.8315-1.067	C	0.2999	0.2354	G	1.97 ×10-8	1.391	1.239-1.562
1	209884953	T	0.2248	0.2354	C	0.3492	0.9421	0.8315-1.067	T	0.2999	0.2354	C	1.97 ×10-8	1.391	1.239-1.562
1	209885318	A	0.2248	0.2354	C	0.3492	0.9421	0.8315-1.067	A	0.2999	0.2354	C	1.97 ×10-8	1.391	1.239-1.562
1	209885509	G	0.296	0.2707	A	0.03476	1.132	1.009-1.271	G	0.1888	0.2707	A	1.06 ×10-12	0.6271	0.5511-0.7135
1	209886578	A	0.2248	0.2354	T	0.3492	0.9421	0.8315-1.067	A	0.2999	0.2354	T	1.97 ×10-8	1.391	1.239-1.562
1	209888435	T	0.2248	0.2354	G	0.3492	0.9421	0.8315-1.067	T	0.2994	0.2354	G	2.56 ×10-8	1.388	1.236-1.558
1	209889601	G	0.2248	0.2354	A	0.3492	0.9421	0.8315-1.067	G	0.2994	0.2354	A	2.56 ×10-8	1.388	1.236-1.558
1	209889659	G	0.2248	0.2354	A	0.3492	0.9421	0.8315-1.067	G	0.2994	0.2354	A	2.56 ×10-8	1.388	1.236-1.558
1	209889689	A	0.2248	0.2354	AC	0.3492	0.9421	0.8315-1.067	A	0.2994	0.2354	AC	2.56 ×10-8	1.388	1.236-1.558
1	209889712	A	0.2248	0.2354	G	0.3492	0.9421	0.8315-1.067	A	0.2994	0.2354	G	2.56 ×10-8	1.388	1.236-1.558
1	209889802	C	0.2248	0.2354	T	0.3492	0.9421	0.8315-1.067	C	0.2994	0.2354	T	2.56 ×10-8	1.388	1.236-1.558
1	209891450	G	0.2248	0.2354	T	0.3492	0.9421	0.8315-1.067	G	0.2994	0.2354	T	2.56 ×10-8	1.388	1.236-1.558
1	209891738	C	0.2248	0.2354	T	0.3492	0.9421	0.8315-1.067	C	0.2994	0.2354	T	2.56 ×10-8	1.388	1.236-1.558
1	209891813	A	0.296	0.2704	G	0.03239	1.134	1.011-1.273	A	0.1888	0.2704	G	1.28 ×10-12	0.6281	0.552-0.7148
1	209891906	A	0.3167	0.2964	C	0.09765	1.1	0.9826-1.232	A	0.2226	0.2964	C	5.32 ×10-10	0.6797	0.6014-0.7682
1	209892035	G	0.2916	0.2684	A	0.05174	1.122	0.9991-1.26	G	0.1867	0.2684	A	1.01 ×10-12	0.6257	0.5496-0.7123

1	209892046	A	0.2248	0.2354	G	0.3492	0.9421	0.8315-1.067	A	0.2994	0.2354	G	2.56 × 10-8	1.388	1.236-1.558
1	209892070	T	0.1953	0.1933	G	0.8474	1.013	0.8877-1.156	T	0.257	0.1933	G	3.23 × 10-9	1.444	1.278-1.631
1	209892706	A	0.2932	0.2707	G	0.05963	1.117	0.9955-1.254	A	0.1883	0.2707	G	7.45 × 10-13	0.6249	0.5491-0.7111
1	209892960	T	0.2243	0.2346	C	0.3617	0.9435	0.8326-1.069	T	0.2988	0.2346	C	2.14 × 10-8	1.391	1.239-1.561
1	209894043	A	0.2194	0.2331	T	0.2222	0.9246	0.8153-1.049	A	0.2967	0.2331	T	2.76 × 10-8	1.388	1.236-1.559
1	209894661	A	0.1685	0.17	G	0.8771	0.989	0.8601-1.137	A	0.2371	0.17	G	7.67 × 10-11	1.517	1.337-1.721
1	209894785	C	0.1942	0.211	T	0.1202	0.9011	0.7902-1.028	C	0.2784	0.211	T	1.25 × 10-9	1.443	1.281-1.625
1	209894822	AAG	0.3435	0.3124	A	0.01231	1.152	1.031-1.287	AAG	0.2409	0.3124	A	3.37 × 10-9	0.6985	0.6199-0.787
1	209895298	G	0.1942	0.211	C	0.1202	0.9011	0.7902-1.028	G	0.2784	0.211	C	1.25 × 10-9	1.443	1.281-1.625
1	209895437	T	0.1685	0.17	G	0.8771	0.989	0.8601-1.137	T	0.2371	0.17	G	7.67 × 10-11	1.517	1.337-1.721
1	209895852	G	0.1942	0.211	T	0.1202	0.9011	0.7902-1.028	G	0.2784	0.211	T	1.25 × 10-9	1.443	1.281-1.625
1	209896212	T	0.3244	0.2945	C	0.01454	1.15	1.028-1.287	T	0.2071	0.2945	C	1.43 × 10-13	0.6258	0.5522-0.7091
1	209897027	A	0.1942	0.211	G	0.1202	0.9011	0.7902-1.028	A	0.2784	0.211	G	1.25 × 10-9	1.443	1.281-1.625
1	209898539	G	0.1942	0.211	A	0.1202	0.9011	0.7902-1.028	G	0.2784	0.211	A	1.25 × 10-9	1.443	1.281-1.625
1	209900307	A	0.3244	0.2945	C	0.01454	1.15	1.028-1.287	A	0.2071	0.2945	C	1.43 × 10-13	0.6258	0.5522-0.7091
1	209900615	C	0.1942	0.211	G	0.1202	0.9011	0.7902-1.028	C	0.2784	0.211	G	1.25 × 10-9	1.443	1.281-1.625
1	209902110	C	0.1942	0.211	T	0.1202	0.9011	0.7902-1.028	C	0.2784	0.211	T	1.25 × 10-9	1.443	1.281-1.625
1	209904252	G	0.3244	0.2943	T	0.014	1.151	1.029-1.289	G	0.2071	0.2943	T	1.57 × 10-13	0.6263	0.5527-0.7096
1	209904433	A	0.1942	0.211	G	0.1202	0.9011	0.7902-1.028	A	0.2784	0.211	G	1.25 × 10-9	1.443	1.281-1.625
1	209905372	C	0.1942	0.211	A	0.1202	0.9011	0.7902-1.028	C	0.2784	0.211	A	1.25 × 10-9	1.443	1.281-1.625
1	209905734	C	0.1942	0.211	G	0.1202	0.9011	0.7902-1.028	C	0.2784	0.211	G	1.25 × 10-9	1.443	1.281-1.625
1	209906097	T	0.1565	0.1367	C	0.03362	1.171	1.012-1.356	T	0.09281	0.1367	C	6.22 × 10-7	0.6461	0.5436-0.768
1	209906675	G	0.1942	0.211	A	0.1202	0.9011	0.7902-1.028	G	0.2784	0.211	A	1.25 × 10-9	1.443	1.281-1.625
1	209906819	G	0.204	0.2205	T	0.1354	0.9064	0.7967-1.031	G	0.287	0.2205	T	3.39 × 10-9	1.423	1.266-1.601
1	209908100	A	0.2123	0.2273	G	0.1771	0.9161	0.8066-1.04	A	0.2967	0.2273	G	1.06 × 10-9	1.434	1.277-1.611
1	209908876	C	0.2101	0.2241	T	0.2045	0.9206	0.8102-1.046	C	0.2924	0.2241	T	1.65 × 10-9	1.43	1.273-1.608

1	209911003	T	0.2123	0.2273	TTTG	0.1771	0.9161	0.8066-1.04	T	0.2967	0.2273	TTTG	1.06 × 10-9	1.434	1.277-1.611
1	209912593	T	0.3239	0.2946	C	0.01706	1.147	1.025-1.283	T	0.2071	0.2946	C	1.30 × 10-13	0.6253	0.5518-0.7085
1	209912742	G	0.3239	0.2946	C	0.01706	1.147	1.025-1.283	G	0.2071	0.2946	C	1.30 × 10-13	0.6253	0.5518-0.7085
1	209913418	T	0.3239	0.2946	C	0.01706	1.147	1.025-1.283	T	0.2071	0.2946	C	1.30 × 10-13	0.6253	0.5518-0.7085
1	209913473	G	0.2588	0.3014	T	0.0004368	0.809	0.7188-0.9106	G	0.3723	0.3014	T	9.30 × 10-9	1.375	1.233-1.533
1	209913707	T	0.3244	0.295	C	0.0163	1.148	1.026-1.284	T	0.2076	0.295	C	1.51 × 10-13	0.6263	0.5528-0.7096
1	209913916	G	0.3239	0.2943	A	0.01582	1.149	1.026-1.285	G	0.2071	0.2943	A	1.57 × 10-13	0.6263	0.5527-0.7096
1	209914100	A	0.3239	0.2943	T	0.01582	1.149	1.026-1.285	A	0.2071	0.2943	T	1.57 × 10-13	0.6263	0.5527-0.7096
1	209914184	G	0.3239	0.2943	GT	0.01582	1.149	1.026-1.285	G	0.2071	0.2943	GT	1.57 × 10-13	0.6263	0.5527-0.7096
1	209914231	T	0.3239	0.2943	A	0.01582	1.149	1.026-1.285	T	0.2071	0.2943	A	1.57 × 10-13	0.6263	0.5527-0.7096
1	209914449	A	0.2095	0.2245	G	0.1776	0.9158	0.8059-1.041	A	0.2935	0.2245	G	1.13 × 10-9	1.435	1.277-1.613
1	209914553	C	0.2095	0.2245	CTTGT	0.1776	0.9158	0.8059-1.041	C	0.2935	0.2245	CTTGT	1.13 × 10-9	1.435	1.277-1.613
1	209915464	G	0.3244	0.2946	A	0.01511	1.15	1.027-1.286	G	0.2071	0.2946	A	1.30 × 10-13	0.6253	0.5518-0.7085
1	209915575	C	0.3244	0.2946	T	0.01511	1.15	1.027-1.286	C	0.2071	0.2946	T	1.30 × 10-13	0.6253	0.5518-0.7085
1	209916073	GTT	0.3244	0.2946	GT	0.01511	1.15	1.027-1.286	GTT	0.2071	0.2946	GT	1.30 × 10-13	0.6253	0.5518-0.7085
1	209916076	GT	0.3244	0.2946	G	0.01511	1.15	1.027-1.286	GT	0.2071	0.2946	G	1.30 × 10-13	0.6253	0.5518-0.7085
1	209916218	G	0.3244	0.2948	A	0.0157	1.149	1.026-1.285	G	0.2071	0.2948	A	1.18 × 10-13	0.6248	0.5514-0.7079
1	209916598	A	0.3244	0.2948	G	0.0157	1.149	1.026-1.285	A	0.2071	0.2948	G	1.18 × 10-13	0.6248	0.5514-0.7079
1	209916789	T	0.1756	0.1747	C	0.928	1.006	0.877-1.155	T	0.2457	0.1747	C	9.68 × 10-12	1.539	1.359-1.743
1	209916884	G	0.2287	0.2485	A	0.08355	0.8965	0.7921-1.015	G	0.3219	0.2485	A	3.67 × 10-10	1.435	1.282-1.608
1	209917729	A	0.3397	0.3129	G	0.03103	1.13	1.011-1.263	A	0.2312	0.3129	G	1.33 × 10-11	0.6605	0.5855-0.7452
1	209918151	A	0.2128	0.2165	G	0.7369	0.9784	0.8611-1.112	A	0.2902	0.2165	G	4.92 × 10-11	1.48	1.316-1.664
1	209919275	T	0.2128	0.2165	C	0.7369	0.9784	0.8611-1.112	T	0.2902	0.2165	C	4.92 × 10-11	1.48	1.316-1.664
1	209919564	T	0.2128	0.2165	TAA	0.7369	0.9784	0.8611-1.112	T	0.2902	0.2165	TAA	4.92 × 10-11	1.48	1.316-1.664
1	209920538	A	0.3266	0.2951	G	0.0103	1.158	1.035-1.296	A	0.2044	0.2951	G	1.62 × 10-14	0.6136	0.5413-0.6956
1	209920545	A	0.1937	0.1919	G	0.8704	1.011	0.8856-1.154	A	0.2639	0.1919	G	2.34 × 10-11	1.51	1.337-1.704

1	209922058	A	0.1942	0.1928	G	0.8918	1.009	0.8841-1.152	A	0.2645	0.1928	G	2.99 ×10-11	1.506	1.334-1.7
1	209922123	T	0.2133	0.217	G	0.7405	0.9787	0.8615-1.112	T	0.2935	0.217	G	1.01 ×10-11	1.499	1.333-1.685
1	209922396	C	0.2133	0.217	CAAAT	0.7405	0.9787	0.8615-1.112	C	0.2935	0.217	CAAAT	1.01 ×10-11	1.499	1.333-1.685
1	209922907	ACTC	0.2133	0.217	A	0.7405	0.9787	0.8615-1.112	ACTC	0.2935	0.217	A	1.01 ×10-11	1.499	1.333-1.685
1	209923154	T	0.2133	0.217	C	0.7405	0.9787	0.8615-1.112	T	0.2935	0.217	C	1.01 ×10-11	1.499	1.333-1.685
1	209924425	T	0.2133	0.2168	C	0.7519	0.9796	0.8623-1.113	T	0.294	0.2168	C	6.56 ×10-12	1.504	1.338-1.691
1	209925058	T	0.1942	0.1928	C	0.8918	1.009	0.8841-1.152	T	0.2645	0.1928	C	2.99 ×10-11	1.506	1.334-1.7
1	209925251	G	0.2133	0.2168	A	0.7519	0.9796	0.8623-1.113	G	0.294	0.2168	A	6.56 ×10-12	1.504	1.338-1.691
1	209925295	T	0.2133	0.2168	C	0.7519	0.9796	0.8623-1.113	T	0.294	0.2168	C	6.56 ×10-12	1.504	1.338-1.691
1	209926027	G	0.3271	0.2955	T	0.009819	1.159	1.036-1.297	G	0.2055	0.2955	T	2.72 ×10-14	0.6167	0.5441-0.699
1	209926069	A	0.2144	0.2168	G	0.8286	0.986	0.8681-1.12	A	0.294	0.2168	G	6.56 ×10-12	1.504	1.338-1.691
1	209926207	G	0.2144	0.217	C	0.8169	0.9851	0.8673-1.119	G	0.294	0.217	C	7.33 ×10-12	1.503	1.337-1.689
1	209926285	G	0.2002	0.1941	GA	0.563	1.039	0.9117-1.185	G	0.2656	0.1941	GA	3.87 ×10-11	1.501	1.33-1.694
1	209926295	AT	0.1953	0.1928	A	0.8105	1.016	0.8905-1.16	AT	0.2645	0.1928	A	2.99 ×10-11	1.506	1.334-1.7
1	209926468	C	0.2144	0.217	A	0.8169	0.9851	0.8673-1.119	C	0.2945	0.217	A	5.30 ×10-12	1.507	1.34-1.693
1	209927448	CTG	0.2084	0.2152	C	0.5381	0.9604	0.8446-1.092	CTG	0.2902	0.2152	C	2.06 ×10-11	1.492	1.326-1.677
1	209927710	C	0.2144	0.2172	T	0.8053	0.9841	0.8664-1.118	C	0.2945	0.2172	T	5.93 ×10-12	1.505	1.339-1.692
1	209928077	A	0.3271	0.2955	G	0.009819	1.159	1.036-1.297	A	0.2055	0.2955	G	2.72 ×10-14	0.6167	0.5441-0.699
1	209929002	A	0.215	0.2172	AAT	0.844	0.9873	0.8693-1.121	A	0.2951	0.2172	AAT	4.27 ×10-12	1.509	1.343-1.696
1	209929044	G	0.215	0.2172	C	0.844	0.9873	0.8693-1.121	G	0.2951	0.2172	C	4.27 ×10-12	1.509	1.343-1.696
1	209929299	G	0.215	0.2172	A	0.844	0.9873	0.8693-1.121	G	0.2951	0.2172	A	4.27 ×10-12	1.509	1.343-1.696
1	209929453	T	0.3271	0.2956	G	0.01022	1.158	1.035-1.296	T	0.2055	0.2956	G	2.46 ×10-14	0.6162	0.5437-0.6984
1	209929579	A	0.1953	0.1928	C	0.8105	1.016	0.8905-1.16	A	0.2645	0.1928	C	2.99 ×10-11	1.506	1.334-1.7
1	209930585	C	0.1953	0.1928	G	0.8105	1.016	0.8905-1.16	C	0.2645	0.1928	G	2.99 ×10-11	1.506	1.334-1.7
1	209930678	T	0.3271	0.2956	C	0.01022	1.158	1.035-1.296	T	0.2055	0.2956	C	2.46 ×10-14	0.6162	0.5437-0.6984
1	209930694	A	0.1953	0.1928	G	0.8105	1.016	0.8905-1.16	A	0.2645	0.1928	G	2.99 ×10-11	1.506	1.334-1.7

1	209931129	T	0.3266	0.2955	C	0.01115	1.156	1.034-1.294	T	0.206	0.2955	C	3.90 × 10-14	0.6187	0.5459-0.7012
1	209931353	A	0.2183	0.2181	C	0.9911	1.001	0.8816-1.136	A	0.2961	0.2181	C	4.38 × 10-12	1.508	1.342-1.695
1	209931799	T	0.2188	0.2178	C	0.9276	1.006	0.8862-1.142	T	0.2961	0.2178	C	3.49 × 10-12	1.511	1.344-1.698
1	209932026	G	0.326	0.2958	A	0.01367	1.152	1.029-1.289	G	0.206	0.2958	A	3.19 × 10-14	0.6177	0.5451-0.7001
1	209932297	A	0.2188	0.2183	T	0.9636	1.003	0.8837-1.138	A	0.2967	0.2183	T	3.53 × 10-12	1.51	1.344-1.697
1	209933331	G	0.2188	0.2186	A	0.9876	1.001	0.882-1.136	G	0.2967	0.2186	A	4.43 × 10-12	1.507	1.341-1.694
1	209933528	A	0.3266	0.2956	G	0.0116	1.156	1.033-1.293	A	0.206	0.2956	G	3.53 × 10-14	0.6182	0.5455-0.7006
1	209933540	G	0.2188	0.2186	A	0.9876	1.001	0.882-1.136	G	0.2967	0.2186	A	4.43 × 10-12	1.507	1.341-1.694
1	209933660	C	0.2856	0.2532	G	0.0057	1.179	1.049-1.325	C	0.177	0.2532	G	1.27 × 10-11	0.6347	0.556-0.7245
1	209933757	C	0.2188	0.2186	G	0.9876	1.001	0.882-1.136	C	0.2967	0.2186	G	4.43 × 10-12	1.507	1.341-1.694
1	209934753	C	0.3266	0.2956	T	0.0116	1.156	1.033-1.293	C	0.206	0.2956	T	3.53 × 10-14	0.6182	0.5455-0.7006
1	209934841	T	0.2188	0.2185	C	0.9756	1.002	0.8828-1.137	T	0.2967	0.2185	C	3.96 × 10-12	1.509	1.343-1.696
1	209935490	G	0.2188	0.219	A	0.9885	0.9991	0.8803-1.134	G	0.2951	0.219	A	1.46 × 10-11	1.493	1.328-1.678
1	209935491	A	0.2188	0.219	C	0.9885	0.9991	0.8803-1.134	A	0.2951	0.219	C	1.46 × 10-11	1.493	1.328-1.678
1	209935778	G	0.2194	0.2196	A	0.9801	0.9984	0.8798-1.133	G	0.2956	0.2196	A	1.65 × 10-11	1.491	1.327-1.675
1	209936064	C	0.2194	0.2186	T	0.9481	1.004	0.8849-1.14	C	0.2961	0.2186	T	6.14 × 10-12	1.504	1.338-1.69
1	209936466	G	0.2144	0.2138	A	0.9558	1.004	0.8834-1.14	G	0.2908	0.2138	A	6.10 × 10-12	1.507	1.34-1.695
1	209936571	G	0.215	0.2143	A	0.9523	1.004	0.8838-1.14	G	0.2913	0.2143	A	6.17 × 10-12	1.507	1.34-1.694
1	209936631	A	0.2128	0.2125	G	0.9786	1.002	0.8815-1.138	A	0.2902	0.2125	G	3.40 × 10-12	1.515	1.347-1.704
1	209936673	G	0.2133	0.2137	C	0.9766	0.9981	0.8784-1.134	G	0.2908	0.2137	C	5.44 × 10-12	1.509	1.342-1.697
1	209936914	T	0.2938	0.3391	C	0.0003017	0.8107	0.7235-0.9086	T	0.4249	0.3391	C	1.50 × 10-11	1.44	1.295-1.601
1	209936964	TCA	0.2943	0.3397	T	0.0002931	0.8105	0.7233-0.9082	TCA	0.4249	0.3397	T	2.18 × 10-11	1.436	1.291-1.597
1	209937168	C	0.2965	0.3401	A	0.0005179	0.8178	0.73-0.9163	C	0.4254	0.3401	A	1.97 × 10-11	1.437	1.292-1.598
1	209937212	C	0.297	0.3402	T	0.0005811	0.8194	0.7314-0.9179	C	0.4254	0.3402	T	2.16 × 10-11	1.436	1.291-1.597
1	209937251	C	0.297	0.3402	G	0.0005811	0.8194	0.7314-0.9179	C	0.4254	0.3402	G	2.16 × 10-11	1.436	1.291-1.597
1	209937382	C	0.297	0.3402	T	0.0005811	0.8194	0.7314-0.9179	C	0.4254	0.3402	T	2.16 × 10-11	1.436	1.291-1.597

1	209937693	A	0.1373	0.1352	G	0.8178	1.018	0.8742-1.186	A	0.1969	0.1352	G	6.91×10^{-11}	1.568	1.369-1.796
1	209937764	G	0.297	0.3406	A	0.0005282	0.8182	0.7303-0.9166	G	0.4254	0.3406	A	2.60×10^{-11}	1.434	1.289-1.594
1	209938075	T	0.3206	0.2855	C	0.00393	1.181	1.055-1.322	T	0.1964	0.2855	C	2.40×10^{-14}	0.6115	0.5384-0.6944
1	209938768	C	0.32	0.2853	T	0.004329	1.179	1.053-1.32	C	0.1964	0.2853	T	2.66×10^{-14}	0.612	0.5388-0.695
1	209938987	A	0.2899	0.3039	G	0.2535	0.9352	0.8336-1.049	A	0.39	0.3039	G	3.90×10^{-12}	1.464	1.314-1.632
1	209939599	T	0.2899	0.3039	C	0.2535	0.9352	0.8336-1.049	T	0.39	0.3039	C	3.90×10^{-12}	1.464	1.314-1.632
1	209939806	T	0.32	0.2853	C	0.004329	1.179	1.053-1.32	T	0.1969	0.2853	C	3.82×10^{-14}	0.614	0.5407-0.6973
1	209940560	A	0.203	0.2009	T	0.8476	1.013	0.8892-1.154	A	0.2715	0.2009	T	1.10×10^{-10}	1.482	1.315-1.671
1	209940675	T	0.203	0.2009	C	0.8476	1.013	0.8892-1.154	T	0.2715	0.2009	C	1.10×10^{-10}	1.482	1.315-1.671
1	209941043	A	0.3184	0.2837	G	0.004246	1.179	1.053-1.321	A	0.1958	0.2837	G	5.04×10^{-14}	0.6149	0.5413-0.6984
1	209942435	C	0.2079	0.2085	G	0.9522	0.9961	0.8756-1.133	C	0.2806	0.2085	G	7.77×10^{-11}	1.48	1.315-1.667
1	209942510	T	0.2949	0.3144	C	0.1141	0.912	0.8135-1.022	T	0.3997	0.3144	C	8.94×10^{-12}	1.452	1.304-1.617
1	209943156	G	0.2763	0.2961	A	0.1017	0.9073	0.8076-1.019	G	0.3755	0.2961	A	1.11×10^{-10}	1.429	1.282-1.594
1	209943893	G	0.3266	0.2875	GGTGT	0.001348	1.202	1.074-1.345	G	0.2001	0.2875	GGTGT	9.02×10^{-14}	0.62	0.5464-0.7036
1	209944167	G	0.2763	0.2961	GGT	0.1017	0.9073	0.8076-1.019	G	0.3755	0.2961	GGT	1.11×10^{-10}	1.429	1.282-1.594
1	209944185	AGT	0.1991	0.2052	AGTGT	0.5716	0.963	0.845-1.097	AGT	0.279	0.2052	AGTGT	2.23×10^{-11}	1.499	1.33-1.688
1	209944365	A	0.3271	0.2877	ATG	0.001212	1.204	1.076-1.347	A	0.2006	0.2877	ATG	1.17×10^{-13}	0.6216	0.5478-0.7053
1	209944398	C	0.2828	0.3082	T	0.03829	0.8851	0.7885-0.9935	C	0.3948	0.3082	T	3.45×10^{-12}	1.464	1.315-1.631
1	209944557	G	0.326	0.2875	GGT	0.001572	1.199	1.071-1.342	G	0.2012	0.2875	GGT	1.83×10^{-13}	0.6242	0.5501-0.7081
1	209944686	C	0.3266	0.2875	G	0.001348	1.202	1.074-1.345	C	0.2012	0.2875	G	1.83×10^{-13}	0.6242	0.5501-0.7081
1	209944801	A	0.2763	0.2961	T	0.1017	0.9073	0.8076-1.019	A	0.3755	0.2961	T	1.11×10^{-10}	1.429	1.282-1.594
1	209944847	GGT	0.2834	0.3081	G	0.04405	0.8882	0.7913-0.9969	GGT	0.3948	0.3081	G	3.12×10^{-12}	1.466	1.316-1.632
1	209945107	G	0.2763	0.2961	GGT	0.1017	0.9073	0.8076-1.019	G	0.3755	0.2961	GGT	1.11×10^{-10}	1.429	1.282-1.594
1	209946027	A	0.3282	0.2668	C	3.03×10^{-7}	1.343	1.199-1.504	A	0.1964	0.2668	C	8.58×10^{-10}	0.6716	0.5911-0.7631
1	209946707	G	0.3507	0.3636	A	0.3114	0.945	0.847-1.054	G	0.441	0.3636	A	1.89×10^{-9}	1.381	1.242-1.534
1	209950486	G	0.1679	0.1861	A	0.07743	0.8826	0.7682-1.014	G	0.25	0.1861	A	1.84×10^{-9}	1.458	1.288-1.649

1	209950681	T	0.3507	0.364	C	0.2992	0.9437	0.8458-1.053	T	0.441	0.364	C	2.22 ×10-9	1.379	1.241-1.532
1	209950760	C	0.1854	0.1918	G	0.5463	0.9595	0.839-1.097	C	0.2918	0.1918	G	4.11 ×10-20	1.737	1.543-1.956
1	209951602	A	0.3332	0.2611	G	1.72 ×10-9	1.414	1.263-1.583	A	0.198	0.2611	G	3.18 ×10-8	0.6984	0.6148-0.7935
1	209951723	T	0.3337	0.2658	G	1.54 ×10-8	1.384	1.236-1.549	T	0.1974	0.2658	G	2.56 ×10-9	0.6796	0.5982-0.7721
1	209951830	G	0.3353	0.2646	T	3.77 ×10-9	1.402	1.253-1.57	G	0.1953	0.2646	T	1.41 ×10-9	0.6744	0.5934-0.7666
1	209952865	G	0.1833	0.2417	C	1.82 ×10-7	0.7039	0.6167-0.8036	G	0.2768	0.2417	C	0.002212	1.201	1.068-1.35
1	209955848	C	0.1816	0.2402	A	1.59 ×10-7	0.7019	0.6147-0.8016	C	0.2741	0.2402	A	0.00304	1.195	1.062-1.344
1	209958140	T	0.5011	0.4534	TGC	0.0003401	1.211	1.09-1.345	T	0.3214	0.4534	TGC	6.33 ×10-24	0.5709	0.5116-0.637
1	209958350	AT	0.1816	0.2399	A	1.84 ×10-7	0.7032	0.6158-0.8031	AT	0.2741	0.2399	A	0.002757	1.197	1.064-1.346
1	209958580	C	0.5	0.4526	T	0.0003662	1.21	1.089-1.343	C	0.3219	0.4526	T	1.79 ×10-23	0.5742	0.5146-0.6407
1	209959168	T	0.4716	0.4049	C	4.33 ×10-7	1.311	1.18-1.457	T	0.2682	0.4049	C	1.44 ×10-26	0.5387	0.4803-0.6041
1	209959614	G	0.4234	0.4638	T	0.002363	0.8489	0.7637-0.9435	G	0.5907	0.4638	T	1.05 ×10-21	1.668	1.501-1.853
1	209960436	A	0.5005	0.4531	G	0.0003615	1.21	1.09-1.344	A	0.3224	0.4531	G	1.88 ×10-23	0.5745	0.5149-0.641
1	209960922	C	0.1816	0.2399	A	1.84 ×10-7	0.7032	0.6158-0.8031	C	0.2741	0.2399	A	0.002757	1.197	1.064-1.346
1	209960925	C	0.5005	0.4532	T	0.0003791	1.209	1.089-1.343	C	0.3224	0.4532	T	1.66 ×10-23	0.5741	0.5145-0.6406
1	209961023	A	0.5005	0.4532	T	0.0003791	1.209	1.089-1.343	A	0.3224	0.4532	T	1.66 ×10-23	0.5741	0.5145-0.6406
1	209962539	C	0.5005	0.4534	T	0.0003976	1.208	1.088-1.342	C	0.323	0.4534	T	2.22 ×10-23	0.5751	0.5154-0.6417
1	209962794	A	0.5011	0.4534	G	0.0003401	1.211	1.09-1.345	A	0.3235	0.4534	G	3.37 ×10-23	0.5765	0.5167-0.6432
1	209963803	A	0.5016	0.4536	G	0.0003048	1.213	1.092-1.347	A	0.324	0.4536	G	4.51 ×10-23	0.5776	0.5177-0.6444
1	209964080	T	0.4721	0.4049	C	3.49 ×10-7	1.314	1.183-1.46	T	0.2682	0.4049	C	1.44 ×10-26	0.5387	0.4803-0.6041
1	209964875	G	0.1778	0.1984	T	0.05068	0.8736	0.7628-1	G	0.3015	0.1984	T	8.86 ×10-21	1.744	1.551-1.961
1	209965283	G	0.3578	0.435	C	4.52 ×10-9	0.7236	0.6493-0.8064	G	0.5671	0.435	C	1.67 ×10-23	1.701	1.532-1.889
1	209965587	G	0.3578	0.435	C	4.52 ×10-9	0.7236	0.6493-0.8064	G	0.5671	0.435	C	1.67 ×10-23	1.701	1.532-1.889
1	209966396	TA	0.1275	0.1919	T	9.49 × 10-10	0.615	0.5285-0.7157	TA	0.1797	0.1919	T	0.2391	0.9224	0.8063-1.055
1	209966402	AAG	0.3616	0.4418	A	1.22 ×10-9	0.7157	0.6424-0.7974	AAG	0.5687	0.4418	A	8.67 ×10-22	1.666	1.5-1.85

1	209966629	A	0.4721	0.4049	G	3.49 ×10-7	1.314	1.183-1.46	A	0.2682	0.4049	G	1.44 ×10-26	0.5387	0.4803-0.6041
1	209966843	A	0.18	0.1991	G	0.0709	0.883	0.7715-1.011	A	0.3031	0.1991	G	4.46 ×10-21	1.75	1.556-1.968
1	209967380	C	0.3578	0.435	T	4.52 ×10-9	0.7236	0.6493-0.8064	C	0.5671	0.435	T	1.67 ×10-23	1.701	1.532-1.889
1	209967651	C	0.134	0.1894	A	5.21 ×10-8	0.6622	0.5704-0.7687	C	0.2049	0.1894	A	0.1388	1.103	0.9688-1.255
1	209968040	T	0.1143	0.1307	C	0.06516	0.8584	0.7298-1.01	T	0.2178	0.1307	C	4.72 ×10-20	1.852	1.621-2.115
1	209968319	C	0.3578	0.435	A	4.52 ×10-9	0.7236	0.6493-0.8064	C	0.5671	0.435	A	1.67 ×10-23	1.701	1.532-1.889
1	209968684	C	0.3578	0.435	A	4.52 ×10-9	0.7236	0.6493-0.8064	C	0.5671	0.435	A	1.67 ×10-23	1.701	1.532-1.889
1	209969183	T	0.1778	0.1986	A	0.0489	0.8727	0.7621-0.9994	T	0.2988	0.1986	A	9.21 ×10-20	1.72	1.529-1.935
1	209970355	G	0.1772	0.1971	C	0.05949	0.8777	0.7662-1.005	G	0.2977	0.1971	C	5.35 ×10-20	1.727	1.535-1.943
1	209970610	G	0.3578	0.435	A	4.52 ×10-9	0.7236	0.6493-0.8064	G	0.5671	0.435	A	1.67 ×10-23	1.701	1.532-1.889
1	209971485	G	0.1816	0.2404	T	1.47 ×10-7	0.7013	0.6141-0.8009	G	0.2736	0.2404	T	0.003705	1.19	1.058-1.339
1	209971528	GA	0.3578	0.4351	G	4.19 ×10-9	0.7232	0.6489-0.8059	GA	0.5671	0.4351	G	1.90 ×10-23	1.7	1.531-1.888
1	209971628	C	0.3578	0.4351	A	4.19 ×10-9	0.7232	0.6489-0.8059	C	0.5671	0.4351	A	1.90 ×10-23	1.7	1.531-1.888
1	209971640	G	0.3578	0.4351	C	4.19 ×10-9	0.7232	0.6489-0.8059	G	0.5671	0.4351	C	1.90 ×10-23	1.7	1.531-1.888
1	209971655	A	0.3578	0.4351	G	4.19 ×10-9	0.7232	0.6489-0.8059	A	0.5671	0.4351	G	1.90 ×10-23	1.7	1.531-1.888
1	209972075	A	0.3578	0.4351	G	4.19 ×10-9	0.7232	0.6489-0.8059	A	0.5671	0.4351	G	1.90 ×10-23	1.7	1.531-1.888
1	209972198	A	0.4743	0.4073	G	3.75 ×10-7	1.313	1.182-1.459	A	0.2698	0.4073	G	9.01 ×10-27	0.5379	0.4797-0.6031
1	209973549	G	0.3578	0.4351	A	4.19 ×10-9	0.7232	0.6489-0.8059	G	0.5671	0.4351	A	1.90 ×10-23	1.7	1.531-1.888
1	209973922	T	0.3572	0.4351	C	3.25 ×10-9	0.7214	0.6473-0.804	T	0.5671	0.4351	C	1.90 ×10-23	1.7	1.531-1.888
1	209974232	A	0.1756	0.1938	C	0.08267	0.8863	0.7733-1.016	A	0.2929	0.1938	C	1.15 ×10-19	1.724	1.531-1.94
1	209975386	G	0.1811	0.2405	A	1.04 ×10-7	0.6981	0.6112-0.7973	G	0.2736	0.2405	A	0.003886	1.189	1.057-1.338
1	209975392	C	0.4825	0.4116	T	7.98 ×10-8	1.333	1.2-1.481	C	0.2725	0.4116	T	2.89 ×10-27	0.5356	0.4778-0.6004
1	209976215	G	0.3567	0.4351	A	2.52 ×10-9	0.7197	0.6458-0.8021	G	0.5665	0.4351	A	2.85 ×10-23	1.697	1.528-1.884
1	209976646	T	0.3567	0.4351	C	2.52 ×10-9	0.7197	0.6458-0.8021	T	0.5665	0.4351	C	2.85 ×10-23	1.697	1.528-1.884
1	209977111	A	0.1761	0.1936	G	0.09547	0.8906	0.7772-1.021	A	0.2924	0.1936	G	1.51 ×10-19	1.721	1.529-1.938
1	209977226	A	0.3567	0.4351	AAC	2.52 ×10-9	0.7197	0.6458-0.8021	A	0.5665	0.4351	AAC	2.85 ×10-23	1.697	1.528-1.884

1	209977844	T	0.4825	0.4116	C	7.98 ×10-8	1.333	1.2-1.481	T	0.272	0.4116	C	1.82 ×10-27	0.5341	0.4765-0.5988
1	209978098	T	0.4825	0.4116	C	7.98 ×10-8	1.333	1.2-1.481	T	0.272	0.4116	C	1.82 ×10-27	0.5341	0.4765-0.5988
1	209978777	T	0.3567	0.4351	G	2.52 ×10-9	0.7197	0.6458-0.8021	T	0.5671	0.4351	G	1.90 ×10-23	1.7	1.531-1.888
1	209979014	G	0.355	0.4346	C	1.46 ×10-9	0.716	0.6424-0.7981	G	0.5671	0.4346	C	1.29 ×10-23	1.704	1.534-1.892
1	209979613	C	0.3567	0.4353	G	2.34 ×10-9	0.7192	0.6453-0.8016	C	0.5665	0.4353	G	3.23 ×10-23	1.695	1.527-1.883
1	209979635	C	0.3567	0.4353	T	2.34 ×10-9	0.7192	0.6453-0.8016	C	0.5665	0.4353	T	3.23 ×10-23	1.695	1.527-1.883
1	209980027	T	0.1811	0.2405	C	1.04 ×10-7	0.6981	0.6112-0.7973	T	0.2736	0.2405	C	0.003886	1.189	1.057-1.338
1	209980155	T	0.3567	0.4353	G	2.34 ×10-9	0.7192	0.6453-0.8016	T	0.5665	0.4353	G	3.23 ×10-23	1.695	1.527-1.883
1	209980489	G	0.3567	0.4353	T	2.34 ×10-9	0.7192	0.6453-0.8016	G	0.566	0.4353	T	4.84 ×10-23	1.692	1.523-1.879
1	209980757	G	0.3567	0.4353	A	2.34 ×10-9	0.7192	0.6453-0.8016	G	0.566	0.4353	A	4.84 ×10-23	1.692	1.523-1.879
1	209981321	CTTTT	0.1794	0.1972	C	0.09097	0.8899	0.7773-1.019	CTTTT	0.294	0.197	2 C	1.24 ×10-18	1.695	1.506-1.907
1	209982025	A	0.5268	0.4754	G	0.000119	1.228	1.106-1.364	A	0.3407	0.4754	G	1.36 ×10-24	0.57	0.5115-0.6353
1	209982293	G	0.1767	0.1949	A	0.08225	0.8864	0.7737-1.016	G	0.2924	0.1949	A	5.23 ×10-19	1.707	1.516-1.921
1	209982372	G	0.5268	0.4754	A	0.000119	1.228	1.106-1.364	G	0.3407	0.4754	A	1.36 ×10-24	0.57	0.5115-0.6353
1	209982408	T	0.5268	0.4754	G	0.000119	1.228	1.106-1.364	T	0.3407	0.4754	G	1.36 ×10-24	0.57	0.5115-0.6353
1	209982412	T	0.5268	0.4754	TAA	0.000119	1.228	1.106-1.364	T	0.3407	0.4754	TAA	1.36 ×10-24	0.57	0.5115-0.6353
1	209982515	C	0.1767	0.1949	T	0.08225	0.8864	0.7737-1.016	C	0.2924	0.1949	T	5.23 ×10-19	1.707	1.516-1.921
1	209982738	G	0.4814	0.4111	A	1.02 ×10-7	1.33	1.197-1.477	G	0.272	0.4111	A	2.71 ×10-27	0.5352	0.4775-0.6
1	209982923	A	0.5268	0.4754	G	0.000119	1.228	1.106-1.364	A	0.3407	0.4754	G	1.36 ×10-24	0.57	0.5115-0.6353
1	209983331	G	0.5268	0.4754	A	0.000119	1.228	1.106-1.364	G	0.3407	0.4754	A	1.36 ×10-24	0.57	0.5115-0.6353
1	209983900	T	0.4814	0.4111	C	1.02 ×10-7	1.33	1.197-1.477	T	0.272	0.4111	C	2.71 ×10-27	0.5352	0.4775-0.6
1	209983916	C	0.4814	0.4111	T	1.02 ×10-7	1.33	1.197-1.477	C	0.272	0.4111	T	2.71 ×10-27	0.5352	0.4775-0.6
1	209984013	T	0.4814	0.4111	G	1.02 ×10-7	1.33	1.197-1.477	T	0.272	0.4111	G	2.71 ×10-27	0.5352	0.4775-0.6
1	209984470	G	0.4814	0.4111	T	1.02 ×10-7	1.33	1.197-1.477	G	0.272	0.4111	T	2.71 ×10-27	0.5352	0.4775-0.6
1	209984824	T	0.4814	0.4111	G	1.02 ×10-7	1.33	1.197-1.477	T	0.272	0.4111	G	2.71 ×10-27	0.5352	0.4775-0.6
1	209985136	T	0.4814	0.4112	C	1.09 ×10-7	1.329	1.196-1.476	T	0.272	0.4112	C	2.37 ×10-27	0.5349	0.4772-0.5996

1	209986672	T	0.1767	0.1951	C	0.07958	0.8855	0.7729-1.015	T	0.2924	0.1951	C	6.10 ×10-19	1.705	1.514-1.919
1	209987801	A	0.1767	0.1949	C	0.08225	0.8864	0.7737-1.016	A	0.2924	0.1949	C	5.23 ×10-19	1.707	1.516-1.921
1	209989092	T	0.5881	0.5063	A	8.64 × 10-10	1.392	1.252-1.548	T	0.3675	0.5063	A	9.56 ×10-26	0.5665	0.5091-0.6304
1	209989270	A	0.1767	0.1951	G	0.07958	0.8855	0.7729-1.015	A	0.2929	0.1951	G	3.98 ×10-19	1.709	1.518-1.924
1	209990087	GTGTA	0.4803	0.4107	G	1.39 ×10-7	1.326	1.194-1.473	GTGTA	0.2715	0.41	7 G	2.22 ×10-27	0.5345	0.4768-0.5992
1	209990549	C	0.1772	0.1953	T	0.08617	0.8879	0.7751-1.017	C	0.2929	0.1953	T	4.65 ×10-19	1.707	1.517-1.922
1	209992043	A	0.1772	0.1953	G	0.08617	0.8879	0.7751-1.017	A	0.2929	0.1953	G	4.65 ×10-19	1.707	1.517-1.922
1	209992127	A	0.5377	0.4824	G	3.40 ×10-5	1.248	1.124-1.386	A	0.3487	0.4824	G	3.71 ×10-24	0.5745	0.5157-0.6399
1	209992501	A	0.4912	0.418	G	3.21 ×10-8	1.344	1.21-1.493	A	0.2795	0.418	G	6.50 ×10-27	0.54	0.4822-0.6049
1	209993366	A	0.1882	0.2465	AT	2.36 ×10-7	0.7085	0.6215-0.8078	A	0.2795	0.2465	AT	0.004256	1.186	1.055-1.333
1	209993366	ATT	0.4732	0.4084	AT	9.15 ×10-7	1.301	0.6215-0.8078	A	0.2795	0.2465	AT	0.004256	1.186	1.055-1.333
1	209993801	G	0.3397	0.3514	A	0.3601	0.9498	0.8506-1.061	G	0.4549	0.3514	A	7.08 ×10-16	1.541	1.387-1.712
1	209994012	G	0.1827	0.2386	C	5.62 ×10-7	0.7136	0.625-0.8148	G	0.2752	0.2386	C	0.001343	1.212	1.078-1.363
1	209994715	T	0.2303	0.3037	C	1.18 ×10-9	0.6859	0.6071-0.7748	T	0.3439	0.3037	C	0.001094	1.201	1.076-1.341
1	209995470	A	0.4962	0.421	G	1.42 ×10-8	1.354	1.219-1.504	A	0.2849	0.421	G	5.70 ×10-26	0.5478	0.4893-0.6132
1	209996542	G	0.2314	0.3059	T	9.35 × 10-10	0.6831	0.6048-0.7715	G	0.3439	0.3059	T	0.002033	1.189	1.065-1.328
1	209997123	C	0.2347	0.3072	CA	2.15 ×10-9	0.6914	0.6125-0.7806	C	0.3455	0.3072	CA	0.001906	1.19	1.066-1.329
1	209997557	GCA	0.3282	0.3482	G	0.1151	0.9146	0.8184-1.022	GCA	0.4662	0.3482	G	3.89 ×10-20	1.635	1.471-1.816
1	209997700	A	0.1838	0.2394	G	6.59 ×10-7	0.7156	0.6269-0.8168	A	0.2741	0.2394	G	0.002376	1.2	1.067-1.35
1	209998053	G	0.2341	0.3076	A	1.38 ×10-9	0.6883	0.6097-0.777	G	0.345	0.3076	A	0.002415	1.186	1.062-1.324
1	209998467	C	0.1761	0.1959	T	0.05978	0.8775	0.7658-1.005	C	0.2902	0.1959	T	6.90 ×10-18	1.678	1.491-1.889
1	209999051	T	0.2341	0.3076	G	1.38 ×10-9	0.6883	0.6097-0.777	T	0.345	0.3076	G	0.002415	1.186	1.062-1.324
1	209999135	C	0.1833	0.2394	G	5.09 ×10-7	0.7129	0.6245-0.8139	C	0.2741	0.2394	G	0.002376	1.2	1.067-1.35
1	209999630	G	0.2341	0.3072	A	1.62 ×10-9	0.6893	0.6106-0.7782	G	0.345	0.3072	A	0.002204	1.187	1.064-1.326

1	210000057	CT	0.2341	0.3072	C	1.62 ×10-9	0.6893	0.6106-0.7782	CT	0.345	0.3072	C	0.002204	1.187	1.064-1.326
1	210000148	C	0.2341	0.3072	T	1.62 ×10-9	0.6893	0.6106-0.7782	C	0.345	0.3072	T	0.002204	1.187	1.064-1.326
1	210000175	AT	0.2341	0.3072	A	1.62 ×10-9	0.6893	0.6106-0.7782	AT	0.345	0.3072	A	0.002204	1.187	1.064-1.326
1	210000282	C	0.1833	0.2391	T	5.87 ×10-7	0.7142	0.6256-0.8154	C	0.2741	0.2391	T	0.00215	1.202	1.069-1.352
1	210000585	C	0.233	0.3047	CT	3.03 ×10-9	0.6932	0.6139-0.7828	C	0.3412	0.3047	CT	0.003021	1.182	1.058-1.319
1	210000693	A	0.2341	0.3072	C	1.62 ×10-9	0.6893	0.6106-0.7782	A	0.345	0.3072	C	0.002204	1.187	1.064-1.326
1	210001048	C	0.2341	0.3072	A	1.62 ×10-9	0.6893	0.6106-0.7782	C	0.345	0.3072	A	0.002204	1.187	1.064-1.326
1	210001125	G	0.2341	0.3072	GC	1.62 ×10-9	0.6893	0.6106-0.7782	G	0.345	0.3072	GC	0.002204	1.187	1.064-1.326
1	210001233	C	0.1772	0.1963	T	0.0706	0.8823	0.7702-1.011	C	0.2902	0.1963	T	9.27 ×10-18	1.675	1.488-1.885
1	210001586	G	0.2341	0.3072	A	1.62 ×10-9	0.6893	0.6106-0.7782	G	0.345	0.3072	A	0.002204	1.187	1.064-1.326
1	210001713	A	0.2341	0.3072	C	1.62 ×10-9	0.6893	0.6106-0.7782	A	0.345	0.3072	C	0.002204	1.187	1.064-1.326
1	210002095	G	0.2341	0.3072	GAC	1.62 ×10-9	0.6893	0.6106-0.7782	G	0.345	0.3072	GAC	0.002204	1.187	1.064-1.326
1	210002099	G	0.2341	0.3072	C	1.62 ×10-9	0.6893	0.6106-0.7782	G	0.345	0.3072	C	0.002204	1.187	1.064-1.326
1	210002123	A	0.186	0.243	AT	3.82 ×10-7	0.7117	0.6239-0.8119	A	0.2736	0.243	AT	0.00774	1.173	1.043-1.32
1	210002353	A	0.2341	0.3072	G	1.62 ×10-9	0.6893	0.6106-0.7782	A	0.345	0.3072	G	0.002204	1.187	1.064-1.326
1	210002838	T	0.18	0.2324	C	2.15 ×10-6	0.7249	0.6343-0.8284	T	0.2677	0.2324	C	0.001843	1.207	1.072-1.359
1	210002994	AATTT	0.2341	0.3072	A	1.62 ×10-9	0.6893	0.6106-0.7782	AATTT	0.345	0.307	2 A	0.002204	1.187	1.064-1.326
1	210003388	C	0.1838	0.2377	A	1.34 ×10-6	0.7221	0.6326-0.8243	C	0.2731	0.2377	A	0.001959	1.205	1.071-1.355
1	210007416	T	0.4847	0.4144	A	1.06 ×10-7	1.329	1.197-1.476	T	0.2886	0.4144	A	1.77 ×10-22	0.5734	0.5123-0.6416
1	210009372	A	0.2341	0.3072	G	1.62 ×10-9	0.6893	0.6106-0.7782	A	0.345	0.3072	G	0.002204	1.187	1.064-1.326
1	210012102	A	0.1811	0.1982	G	0.1041	0.8942	0.7814-1.023	A	0.2902	0.1982	G	5.30 ×10-17	1.654	1.469-1.862
1	210014093	A	0.1816	0.1999	T	0.08436	0.8883	0.7764-1.016	A	0.2908	0.1999	T	1.47 ×10-16	1.641	1.458-1.847
1	210014603	C	0.1816	0.1997	G	0.08714	0.8892	0.7772-1.017	C	0.2902	0.1997	G	1.89 ×10-16	1.638	1.456-1.844
1	210014752	T	0.1833	0.2361	C	2.08 ×10-6	0.7261	0.636-0.8291	T	0.2725	0.2361	C	0.001371	1.212	1.077-1.364
1	210015568	GCT	0.1816	0.1999	G	0.08436	0.8883	0.7764-1.016	GCT	0.2908	0.1999	G	1.47 ×10-16	1.641	1.458-1.847
1	210019824	A	0.1816	0.1997	G	0.08714	0.8892	0.7772-1.017	A	0.2908	0.1997	G	1.28 ×10-16	1.643	1.459-1.849

1	210020013	A	0.1816	0.1999	C	0.08436	0.8883	0.7764-1.016	A	0.2908	0.1999	C	1.47 ×10-16	1.641	1.458-1.847
1	210020971	T	0.1833	0.2362	C	1.95 ×10-6	0.7255	0.6354-0.8283	T	0.2725	0.2362	C	0.001444	1.211	1.076-1.363
1	210022179	A	0.1816	0.1997	G	0.08714	0.8892	0.7772-1.017	A	0.2908	0.1997	G	1.28 ×10-16	1.643	1.459-1.849
1	210022893	G	0.1816	0.1992	C	0.09593	0.892	0.7796-1.021	G	0.2902	0.1992	C	1.24 ×10-16	1.644	1.46-1.85
1	210022901	T	0.4836	0.4147	C	1.91 ×10-7	1.322	1.19-1.468	T	0.2935	0.4147	C	5.49 ×10-21	0.5861	0.524-0.6556
1	210027026	G	0.1915	0.2391	A	2.16 ×10-5	0.7538	0.6615-0.859	G	0.324	0.2391	A	2.51 ×10-13	1.526	1.362-1.71
1	210027565	G	0.1165	0.1415	C	0.006287	0.8001	0.6817-0.9392	G	0.2173	0.1415	C	5.71 ×10-15	1.684	1.476-1.921
1	210030755	G	0.4847	0.4245	T	5.60 ×10-6	1.275	1.148-1.416	G	0.3069	0.4245	T	1.14 ×10-19	0.6002	0.5372-0.6705
1	210031154	A	0.1417	0.1657	G	0.01408	0.831	0.7167-0.9635	A	0.2457	0.1657	G	7.35 ×10-15	1.64	1.447-1.859
1	210032055	G	0.1729	0.2089	C	0.0007602	0.7917	0.6908-0.9072	G	0.2859	0.2089	C	3.84 ×10-12	1.517	1.348-1.707
1	210032392	A	0.1461	0.1695	G	0.01757	0.8378	0.7239-0.9697	A	0.2403	0.1695	G	6.79 ×10-12	1.55	1.367-1.757
1	210038317	A	0.494	0.4469	AG	0.0004029	1.208	1.088-1.342	A	0.3348	0.4469	AG	1.01 ×10-17	0.6228	0.5586-0.6943
1	210041297	T	0.145	0.1705	G	0.009805	0.8246	0.7123-0.9547	T	0.2361	0.1705	G	2.11 ×10-10	1.503	1.325-1.705
1	210042077	T	0.483	0.4195	C	1.60 ×10-6	1.293	1.164-1.436	T	0.3117	0.4195	C	8.24 ×10-17	0.6265	0.561-0.6998
1	210042456	C	0.4956	0.4466	T	0.0002275	1.218	1.097-1.352	C	0.3385	0.4466	T	1.47 ×10-16	0.6342	0.569-0.7069
1	210043135	G	0.145	0.1712	A	0.008136	0.8208	0.709-0.9502	G	0.2361	0.1712	A	3.34 ×10-10	1.496	1.319-1.697
1	210043652	CT	0.145	0.1712	C	0.008136	0.8208	0.709-0.9502	CT	0.2366	0.1712	C	2.42 ×10-10	1.5	1.323-1.702
1	210046786	A	0.4803	0.4147	G	7.02 ×10-7	1.304	1.174-1.449	A	0.3058	0.4147	G	3.42 ×10-17	0.6216	0.5563-0.6946
1	210048819	T	0.494	0.4453	G	0.0002486	1.216	1.095-1.351	T	0.3364	0.4453	G	8.41 ×10-17	0.6315	0.5665-0.704
1	210049297	A	0.2659	0.2254	G	0.0003559	1.244	1.103-1.403	A	0.1604	0.2254	G	1.70 ×10-9	0.6564	0.572-0.7533
1	210049893	C	0.4776	0.4131	T	1.05 ×10-6	1.299	1.169-1.443	C	0.3042	0.4131	T	3.34 ×10-17	0.6212	0.5558-0.6942
1	210049901	T	0.4765	0.4126	C	1.31 ×10-6	1.296	1.167-1.44	T	0.3042	0.4126	C	4.58 ×10-17	0.6224	0.5569-0.6956
1	210050077	A	0.4765	0.4126	T	1.31 ×10-6	1.296	1.167-1.44	A	0.3026	0.4126	T	1.55 ×10-17	0.6177	0.5526-0.6905
1	210050518	C	0.4765	0.4121	A	1.08 ×10-6	1.299	1.169-1.442	C	0.3026	0.4121	A	2.13 ×10-17	0.619	0.5538-0.6919
1	210051377	A	0.1499	0.1767	G	0.007635	0.8217	0.7111-0.9494	A	0.242	0.1767	G	4.04 ×10-10	1.487	1.313-1.685
1	210051454	A	0.4666	0.4035	G	1.61 ×10-6	1.294	1.164-1.437	A	0.2999	0.4035	G	7.98 ×10-16	0.6334	0.5665-0.7082

1	210051740	CG	0.1499	0.1767	C	0.007635	0.8217	0.7111-0.9494	CG	0.242	0.1767	C	4.04 ×10-10	1.487	1.313-1.685
1	210051748	A	0.4748	0.4114	AAATT	1.56 ×10-6	1.294	1.164-1.437	A	0.302	0.4114	AAATT	2.27 ×10-17	0.6191	0.5538-0.6921
1	210051750	G	0.1461	0.1745	A	0.004352	0.809	0.6992-0.9361	G	0.2334	0.1745	A	1.36 ×10-8	1.44	1.269-1.634
1	210053096	A	0.1559	0.1821	G	0.009865	0.8293	0.7193-0.9561	A	0.2441	0.1821	G	4.12 ×10-9	1.45	1.28-1.642
1	210054211	A	0.4633	0.403	G	4.49 ×10-6	1.279	1.151-1.421	A	0.2956	0.403	G	6.51 ×10-17	0.6218	0.5559-0.6955
1	210054435	C	0.4628	0.4028	T	5.14 ×10-6	1.277	1.15-1.419	C	0.2956	0.4028	T	7.23 ×10-17	0.6222	0.5563-0.696
1	210054718	T	0.4628	0.4026	C	4.84 ×10-6	1.278	1.15-1.42	T	0.2951	0.4026	C	5.61 ×10-17	0.621	0.5552-0.6947
1	210055630	T	0.1559	0.1821	C	0.009865	0.8293	0.7193-0.9561	T	0.2441	0.1821	C	4.12 ×10-9	1.45	1.28-1.642
1	210056359	C	0.4606	0.4013	T	6.52 ×10-6	1.274	1.147-1.416	C	0.2945	0.4013	T	9.05 ×10-17	0.6229	0.5568-0.6968
1	210056576	A	0.2659	0.2261	G	0.0004489	1.239	1.099-1.398	A	0.1588	0.2261	G	4.56 ×10-10	0.6461	0.5628-0.7418
1	210056906	CAG	0.4322	0.3837	C	0.0002053	1.222	1.099-1.359	CAG	0.2902	0.3837	C	2.13 ×10-13	0.6568	0.5868-0.7351
1	210057532	C	0.4759	0.4214	A	3.74 ×10-5	1.247	1.123-1.385	C	0.3385	0.4214	A	1.81 ×10-10	0.7028	0.6304-0.7835
1	210057547	G	0.2516	0.2372	A	0.2066	1.081	0.9578-1.22	G	0.1781	0.2372	A	8.49 ×10-8	0.6968	0.6102-0.7957
1	210057690	T	0.2522	0.2374	C	0.195	1.083	0.9598-1.223	T	0.1781	0.2374	C	7.86 ×10-8	0.6962	0.6097-0.7949
1	210057717	G	0.2522	0.2374	A	0.195	1.083	0.9598-1.223	G	0.1781	0.2374	A	7.86 ×10-8	0.6962	0.6097-0.7949
1	210058242	G	0.2527	0.2377	C	0.1888	1.085	0.9609-1.224	G	0.177	0.2377	C	3.85 ×10-8	0.6898	0.604-0.7879
1	210058735	T	0.2527	0.2376	C	0.1839	1.085	0.9618-1.225	T	0.177	0.2376	C	4.17 ×10-8	0.6904	0.6045-0.7886
1	210058753	G	0.2527	0.2376	T	0.1839	1.085	0.9618-1.225	G	0.1776	0.2376	T	5.51 ×10-8	0.693	0.6068-0.7914
1	210059338	A	0.2527	0.2377	G	0.1888	1.085	0.9609-1.224	A	0.1781	0.2377	G	6.73 ×10-8	0.6949	0.6086-0.7935
1	210059792	A	0.2527	0.2377	G	0.1888	1.085	0.9609-1.224	A	0.1781	0.2377	G	6.73 ×10-8	0.6949	0.6086-0.7935
1	210060201	G	0.2533	0.2377	A	0.1733	1.088	0.9637-1.227	G	0.1781	0.2377	A	6.73 ×10-8	0.6949	0.6086-0.7935
1	210060376	C	0.2533	0.2377	A	0.1733	1.088	0.9637-1.227	C	0.1781	0.2377	A	6.73 ×10-8	0.6949	0.6086-0.7935
1	210060512	G	0.2527	0.2377	A	0.1888	1.085	0.9609-1.224	G	0.1781	0.2377	A	6.73 ×10-8	0.6949	0.6086-0.7935
1	210060563	G	0.2549	0.2389	A	0.1611	1.09	0.9662-1.23	G	0.1792	0.2389	A	6.80 ×10-8	0.6955	0.6093-0.794
1	210060639	T	0.2527	0.2377	C	0.1888	1.085	0.9609-1.224	T	0.1781	0.2377	C	6.73 ×10-8	0.6949	0.6086-0.7935
1	210061320	T	0.2522	0.2377	C	0.2054	1.081	0.9581-1.221	T	0.1781	0.2377	C	6.73 ×10-8	0.6949	0.6086-0.7935

1	210061609	G	0.2527	0.2376	A	0.1839	1.085	0.9618-1.225	G	0.1781	0.2376	A	7.28 × 10-8	0.6955	0.6091-0.7942
1	210061617	G	0.2527	0.2376	A	0.1839	1.085	0.9618-1.225	G	0.1781	0.2376	A	7.28 × 10-8	0.6955	0.6091-0.7942
1	210061693	C	0.2527	0.2376	G	0.1839	1.085	0.9618-1.225	C	0.1781	0.2376	G	7.28 × 10-8	0.6955	0.6091-0.7942
1	210061753	G	0.2527	0.2376	C	0.1839	1.085	0.9618-1.225	G	0.1781	0.2376	C	7.28 × 10-8	0.6955	0.6091-0.7942
1	210061873	C	0.2527	0.2376	T	0.1839	1.085	0.9618-1.225	C	0.1781	0.2376	T	7.28 × 10-8	0.6955	0.6091-0.7942
1	210061900	T	0.2527	0.2376	C	0.1839	1.085	0.9618-1.225	T	0.1781	0.2376	C	7.28 × 10-8	0.6955	0.6091-0.7942
1	210062371	G	0.2527	0.2376	A	0.1839	1.085	0.9618-1.225	G	0.1776	0.2376	A	5.51 × 10-8	0.693	0.6068-0.7914
1	210062385	T	0.2527	0.2376	C	0.1839	1.085	0.9618-1.225	T	0.1776	0.2376	C	5.51 × 10-8	0.693	0.6068-0.7914
1	210062413	A	0.2527	0.2376	G	0.1839	1.085	0.9618-1.225	A	0.1776	0.2376	G	5.51 × 10-8	0.693	0.6068-0.7914
1	210062444	A	0.2527	0.2376	G	0.1839	1.085	0.9618-1.225	A	0.1776	0.2376	G	5.51 × 10-8	0.693	0.6068-0.7914
1	210062455	T	0.2527	0.2376	C	0.1839	1.085	0.9618-1.225	T	0.1776	0.2376	C	5.51 × 10-8	0.693	0.6068-0.7914
1	210062857	T	0.2527	0.2376	C	0.1839	1.085	0.9618-1.225	T	0.1776	0.2376	C	5.51 × 10-8	0.693	0.6068-0.7914
1	210063307	A	0.2527	0.2376	G	0.1839	1.085	0.9618-1.225	A	0.1776	0.2376	G	5.51 × 10-8	0.693	0.6068-0.7914
1	210064037	C	0.2527	0.2376	T	0.1839	1.085	0.9618-1.225	C	0.1776	0.2376	T	5.51 × 10-8	0.693	0.6068-0.7914
1	210064104	T	0.2527	0.2376	A	0.1839	1.085	0.9618-1.225	T	0.1776	0.2376	A	5.51 × 10-8	0.693	0.6068-0.7914
1	210064601	G	0.2522	0.2376	A	0.2001	1.082	0.9589-1.222	G	0.1781	0.2376	A	7.28 × 10-8	0.6955	0.6091-0.7942
1	210064703	A	0.2522	0.2376	G	0.2001	1.082	0.9589-1.222	A	0.1781	0.2376	G	7.28 × 10-8	0.6955	0.6091-0.7942
1	210064774	T	0.2522	0.2376	A	0.2001	1.082	0.9589-1.222	T	0.1781	0.2376	A	7.28 × 10-8	0.6955	0.6091-0.7942
1	210065331	A	0.2522	0.2376	C	0.2001	1.082	0.9589-1.222	A	0.1781	0.2376	C	7.28 × 10-8	0.6955	0.6091-0.7942
1	210065554	G	0.2522	0.2376	A	0.2001	1.082	0.9589-1.222	G	0.1781	0.2376	A	7.28 × 10-8	0.6955	0.6091-0.7942
1	210066093	C	0.2522	0.2376	T	0.2001	1.082	0.9589-1.222	C	0.1781	0.2376	T	7.28 × 10-8	0.6955	0.6091-0.7942
1	210066475	G	0.2522	0.2376	A	0.2001	1.082	0.9589-1.222	G	0.1781	0.2376	A	7.28 × 10-8	0.6955	0.6091-0.7942
1	210066477	TAA	0.2522	0.2367	T	0.1754	1.087	0.9633-1.227	TAA	0.1781	0.2367	T	1.07 × 10-7	0.6987	0.6119-0.7979
1	210066563	G	0.2522	0.2374	A	0.195	1.083	0.9598-1.223	G	0.1781	0.2374	A	7.86 × 10-8	0.6962	0.6097-0.7949
1	210066733	T	0.2527	0.2372	A	0.1743	1.087	0.9635-1.227	T	0.1781	0.2372	A	8.49 × 10-8	0.6968	0.6102-0.7957
1	210066804	T	0.2527	0.2372	G	0.1743	1.087	0.9635-1.227	T	0.1781	0.2372	G	8.49 × 10-8	0.6968	0.6102-0.7957

1	210066842	A	0.2527	0.2372	G	0.1743	1.087	0.9635-1.227	A	0.1781	0.2372	G		8.49 ×10-8	0.6968	0.6102-0.7957
1	210066875	A	0.2527	0.2372	G	0.1743	1.087	0.9635-1.227	A	0.1781	0.2372	G		8.49 ×10-8	0.6968	0.6102-0.7957
1	210067133	T	0.2533	0.2372	C	0.1597	1.091	0.9664-1.231	T	0.1781	0.2372	C		8.49 ×10-8	0.6968	0.6102-0.7957
1	210067388	C	0.483	0.4257	CT	1.50 ×10-5	1.261	1.135-1.4	C	0.3525	0.4257	CT		1.91 ×10-8	0.7344	0.6593-0.818
1	210067436	A	0.2533	0.2372	G	0.1597	1.091	0.9664-1.231	A	0.1781	0.2372	G		8.49 ×10-8	0.6968	0.6102-0.7957
1	210067916	T	0.2533	0.2372	C	0.1597	1.091	0.9664-1.231	T	0.1781	0.2372	C		8.49 ×10-8	0.6968	0.6102-0.7957
1	210068117	G	0.3747	0.4463	A	6.26 ×10-8	0.7437	0.6679-0.828	G	0.5279	0.4463	A		6.57 ×10-10	1.388	1.25-1.54
1	210068227	T	0.2538	0.2369	C	0.138	1.096	0.971-1.237	T	0.1786	0.2369	C		1.30 ×10-7	0.7006	0.6137-0.8
1	210069196	T	0.2538	0.2369	C	0.138	1.096	0.971-1.237	T	0.1786	0.2369	C		1.30 ×10-7	0.7006	0.6137-0.8
1	210072343	A	0.2538	0.2376	G	0.1544	1.092	0.9675-1.232	A	0.1776	0.2376	G		5.51 ×10-8	0.693	0.6068-0.7914
1	210074195	T	0.2527	0.2371	C	0.1697	1.088	0.9644-1.229	T	0.177	0.2371	C		5.28 ×10-8	0.6923	0.6062-0.7908
1	210075317	A	0.3889	0.3675	C	0.09584	1.096	0.9839-1.22	A	0.3047	0.3675	C		7.27 ×10-7	0.7545	0.6747-0.8436
1	210075755	G	0.2527	0.2371	A	0.1697	1.088	0.9644-1.229	G	0.177	0.2371	A		5.28 ×10-8	0.6923	0.6062-0.7908
1	210076223	A	0.3889	0.3675	G	0.09584	1.096	0.9839-1.22	A	0.3047	0.3675	G		7.27 ×10-7	0.7545	0.6747-0.8436
1	210076234	C	0.3889	0.3675	T	0.09584	1.096	0.9839-1.22	C	0.3047	0.3675	T		7.27 ×10-7	0.7545	0.6747-0.8436
1	210076884	A	0.3758	0.4463	T	9.92 ×10-8	0.7471	0.6711-0.8319	A	0.529	0.4463	T		3.92 ×10-10	1.394	1.256-1.547
1	210076950	T	0.4852	0.4234	C	3.00 ×10-6	1.284	1.156-1.426	T	0.3439	0.4234	C		9.98 ×10-10	0.7139	0.6406-0.7956
1	210077087	A	0.4852	0.4234	G	3.00 ×10-6	1.284	1.156-1.426	A	0.3439	0.4234	G		9.98 ×10-10	0.7139	0.6406-0.7956
1	210077113	G	0.3753	0.4461	A	8.44 ×10-8	0.7459	0.6699-0.8305	G	0.5295	0.4461	A		2.79 ×10-10	1.397	1.259-1.551
1	210077605	G	0.3753	0.4461	A	8.44 ×10-8	0.7459	0.6699-0.8305	G	0.5295	0.4461	A		2.79 ×10-10	1.397	1.259-1.551
1	210077882	A	0.4852	0.4234	C	3.00 ×10-6	1.284	1.156-1.426	A	0.3439	0.4234	C		9.98 ×10-10	0.7139	0.6406-0.7956
1	210078053	<CN0>	0.3758	0.4459	G	1.14 ×10-7	0.7481	0.672-0.833	<CN0>	0.5295	0.445	9	G	2.57 ×10-10	1.398	1.26-1.552
1	210081921	A	0.25	0.2332	T	0.1401	1.096	0.9704-1.237	A	0.1754	0.2332	T		1.37 ×10-7	0.6994	0.612-0.7992
1	210086341	T	0.3786	0.4463	C	3.05 ×10-7	0.7559	0.679-0.8415	T	0.5279	0.4463	C		6.57 ×10-10	1.388	1.25-1.54
1	210086539	A	0.3786	0.4463	G	3.05 ×10-7	0.7559	0.679-0.8415	A	0.5279	0.4463	G		6.57 ×10-10	1.388	1.25-1.54
1	210087236	T	0.372	0.4378	C	6.07 ×10-7	0.7607	0.6831-0.8471	T	0.5204	0.4378	C		3.95 ×10-10	1.393	1.256-1.546

1	210092938	A	0.3654	0.4326	G	3.29 × 10-7	0.7552	0.6779-0.8413	A	0.5198	0.4326	G	3.85 × 10-11	1.42	1.279-1.576
1	210092962	GC	0.3654	0.4321	G	4.00 × 10-7	0.7567	0.6792-0.843	GC	0.5193	0.4321	G	3.91 × 10-11	1.42	1.279-1.575
1	210092986	C	0.3676	0.4356	T	2.45 × 10-7	0.7531	0.6761-0.8389	C	0.522	0.4356	T	6.04 × 10-11	1.415	1.275-1.57
1	210093001	T	0.2522	0.2372	C	0.19	1.084	0.9607-1.224	T	0.177	0.2372	C	4.88 × 10-8	0.6917	0.6056-0.79
1	210093031	A	0.4863	0.4235	G	2.13 × 10-6	1.289	1.16-1.431	A	0.3444	0.4235	G	1.20 × 10-9	0.7151	0.6417-0.7969
1	210093087	G	0.3786	0.4463	GAAA	3.05 × 10-7	0.7559	0.679-0.8415	G	0.5279	0.4463	GAAA	6.57 × 10-10	1.388	1.25-1.54
1	210093097	G	0.3753	0.4421	A	4.18 × 10-7	0.758	0.6808-0.844	G	0.5263	0.4421	A	1.87 × 10-10	1.402	1.263-1.556
1	210093107	T	0.4852	0.4237	TC	3.39 × 10-6	1.282	1.154-1.424	T	0.3444	0.4237	TC	1.11 × 10-9	0.7146	0.6412-0.7964
1	210093494	T	0.3786	0.4463	C	3.05 × 10-7	0.7559	0.679-0.8415	T	0.5279	0.4463	C	6.57 × 10-10	1.388	1.25-1.54
1	210093571	G	0.3786	0.4463	A	3.05 × 10-7	0.7559	0.679-0.8415	G	0.5279	0.4463	A	6.57 × 10-10	1.388	1.25-1.54
1	210093585	C	0.3786	0.4463	A	3.05 × 10-7	0.7559	0.679-0.8415	C	0.5279	0.4463	A	6.57 × 10-10	1.388	1.25-1.54
1	210093644	G	0.2527	0.2372	C	0.1743	1.087	0.9635-1.227	G	0.1776	0.2372	C	6.44 × 10-8	0.6943	0.6079-0.7928
1	210094817	T	0.4869	0.4239	A	1.97 × 10-6	1.29	1.161-1.432	T	0.3444	0.4239	A	1.03 × 10-9	0.7141	0.6408-0.7958
1	210094835	C	0.3786	0.4463	T	3.05 × 10-7	0.7559	0.679-0.8415	C	0.5279	0.4463	T	6.57 × 10-10	1.388	1.25-1.54
1	210095016	T	0.3786	0.4463	C	3.05 × 10-7	0.7559	0.679-0.8415	T	0.5279	0.4463	C	6.57 × 10-10	1.388	1.25-1.54
1	210095057	A	0.4869	0.4239	G	1.97 × 10-6	1.29	1.161-1.432	A	0.345	0.4239	G	1.33 × 10-9	0.7158	0.6424-0.7977
1	210095201	T	0.4874	0.4239	C	1.61 × 10-6	1.293	1.164-1.436	T	0.3455	0.4239	C	1.72 × 10-9	0.7175	0.6439-0.7996
1	210095235	G	0.3786	0.4463	A	3.05 × 10-7	0.7559	0.679-0.8415	G	0.5279	0.4463	A	6.57 × 10-10	1.388	1.25-1.54
1	210095527	T	0.3786	0.4463	C	3.05 × 10-7	0.7559	0.679-0.8415	T	0.5279	0.4463	C	6.57 × 10-10	1.388	1.25-1.54
1	210095899	G	0.3796	0.4469	A	3.64 × 10-7	0.7574	0.6804-0.8431	G	0.5279	0.4469	A	9.04 × 10-10	1.384	1.247-1.536
1	210096995	C	0.4179	0.4917	A	3.16 × 10-8	0.7423	0.6678-0.8251	C	0.5842	0.4917	A	2.85 × 10-12	1.453	1.308-1.613
1	210099983	G	0.4902	0.4249	C	8.35 × 10-7	1.301	1.172-1.446	G	0.3476	0.4249	C	3.01 × 10-9	0.7214	0.6475-0.8038
1	210100459	C	0.378	0.4448	T	4.40 × 10-7	0.7587	0.6815-0.8447	C	0.5231	0.4448	T	3.11 × 10-9	1.369	1.234-1.519
1	210102027	G	0.378	0.4448	A	4.40 × 10-7	0.7587	0.6815-0.8447	G	0.5231	0.4448	A	3.11 × 10-9	1.369	1.234-1.519
1	210104501	A	0.2522	0.2337	T	0.1048	1.106	0.9793-1.248	A	0.1786	0.2337	T	5.37 × 10-7	0.713	0.6244-0.8142
1	210104598	G	0.4907	0.4249	A	6.76 × 10-7	1.304	1.174-1.449	G	0.3498	0.4249	A	8.16 × 10-9	0.7283	0.6537-0.8113

1	210104743	T	0.4907	0.4249	A	6.76 ×10-7	1.304	1.174-1.449	T	0.3498	0.4249	A	8.16 ×10-9	0.7283	0.6537-0.8113
1	210105338	G	0.378	0.4449	A	4.13 ×10-7	0.7582	0.681-0.8441	G	0.5231	0.4449	A	3.36 ×10-9	1.368	1.233-1.518
1	210105775	C	0.378	0.4449	A	4.13 ×10-7	0.7582	1.18-1.455	T	0.3509	0.4253	A	1.07 ×10-8	0.7302	0.6555-0.8135
1	210105775	T	0.4923	0.4253	A	4.34 ×10-7	1.31	1.18-1.455	T	0.3509	0.4253	A	1.07 ×10-8	0.7302	0.6555-0.8135
1	210107020	A	0.4923	0.4253	G	4.34 ×10-7	1.31	1.18-1.455	A	0.3519	0.4253	G	1.73 ×10-8	0.7337	0.6586-0.8173
1	210107230	G	0.378	0.4449	A	4.13 ×10-7	0.7582	0.681-0.8441	G	0.5231	0.4449	A	3.36 ×10-9	1.368	1.233-1.518
1	210107289	T	0.4923	0.4258	TCAAA	5.29 ×10-7	1.308	1.177-1.452	T	0.3519	0.4258	TCAAA	1.40 ×10-8	0.7322	0.6573-0.8156
1	210107579	C	0.378	0.4448	A	4.40 ×10-7	0.7587	0.6815-0.8447	C	0.5231	0.4448	A	3.11 ×10-9	1.369	1.234-1.519
1	210108196	T	0.4923	0.4262	A	6.04 ×10-7	1.306	1.176-1.45	T	0.3519	0.4262	A	1.21 ×10-8	0.7312	0.6564-0.8145
1	210108207	T	0.378	0.4449	G	4.13 ×10-7	0.7582	0.681-0.8441	T	0.5231	0.4449	G	3.36 ×10-9	1.368	1.233-1.518
1	210108350	C	0.378	0.4449	T	4.13 ×10-7	0.7582	0.681-0.8441	C	0.5231	0.4449	T	3.36 ×10-9	1.368	1.233-1.518
1	210108473	G	0.378	0.4449	A	4.13 ×10-7	0.7582	0.681-0.8441	G	0.5231	0.4449	A	3.36 ×10-9	1.368	1.233-1.518
1	210108635	C	0.2533	0.2346	CTACG	0.1003	1.107	0.9806-1.249	C	0.1792	0.2346	CTACG	4.83 ×10-7	0.7123	0.6239-0.8133
1	210109286	A	0.378	0.4449	T	4.13 ×10-7	0.7582	0.681-0.8441	A	0.5231	0.4449	T	3.36 ×10-9	1.368	1.233-1.518
1	210110529	A	0.378	0.4448	G	4.40 ×10-7	0.7587	0.6815-0.8447	A	0.5231	0.4448	G	3.11 ×10-9	1.369	1.234-1.519
1	210110537	C	0.378	0.4448	T	4.40 ×10-7	0.7587	0.6815-0.8447	C	0.5231	0.4448	T	3.11 ×10-9	1.369	1.234-1.519
1	210111733	A	0.3764	0.4416	G	7.80 ×10-7	0.7631	0.6854-0.8496	A	0.5193	0.4416	G	4.03 ×10-9	1.366	1.231-1.516
1	210112077	A	0.5082	0.4416	C	5.53 ×10-7	1.307	1.177-1.451	A	0.3653	0.4416	C	5.76 ×10-9	0.7279	0.654-0.8102
1	210115714	G	0.4984	0.4345	C	1.51 ×10-6	1.293	1.164-1.436	G	0.3589	0.4345	C	7.31 ×10-9	0.7287	0.6544-0.8114
1	210119391	G	0.3725	0.438	A	7.06 ×10-7	0.7619	0.6842-0.8485	G	0.5161	0.438	A	3.23 ×10-9	1.369	1.233-1.519
1	210120914	T	0.2533	0.2395	C	0.2303	1.077	0.9542-1.215	T	0.1829	0.2395	C	3.30 ×10-7	0.7108	0.6232-0.8106
1	210121141	C	0.3725	0.4376	T	8.02 ×10-7	0.763	0.6851-0.8497	C	0.5161	0.4376	T	2.77 ×10-9	1.371	1.235-1.521
1	210122652	A	0.5011	0.4361	G	1.01 ×10-6	1.299	1.169-1.442	A	0.36	0.4361	G	5.70 ×10-9	0.7272	0.6531-0.8096
1	210123526	A	0.5011	0.4363	G	1.08 ×10-6	1.298	1.168-1.441	A	0.3605	0.4363	G	6.77 ×10-9	0.7284	0.6542-0.811
1	210123774	G	0.5011	0.4363	A	1.08 ×10-6	1.298	1.168-1.441	G	0.3605	0.4363	A	6.77 ×10-9	0.7284	0.6542-0.811
1	210124279	G	0.5011	0.4365	A	1.15 ×10-6	1.297	1.168-1.44	G	0.3605	0.4365	A	6.28 ×10-9	0.7279	0.6538-0.8104

1	210124855	T	0.3709	0.4375	C	4.48 × 10-7	0.7581	0.6807-0.8443	T	0.5156	0.4375	C	3.28 × 10-9	1.369	1.233-1.519
1	210127195	G	0.3714	0.4373	C	5.93 × 10-7	0.7604	0.6828-0.8469	G	0.5156	0.4373	C	3.04 × 10-9	1.369	1.234-1.52
1	210128231	G	0.5011	0.4368	A	1.31 × 10-6	1.295	1.166-1.438	G	0.3605	0.4368	A	5.41 × 10-9	0.7269	0.6529-0.8093
1	210130204	A	0.5011	0.437	T	1.39 × 10-6	1.294	1.165-1.437	A	0.3611	0.437	T	6.43 × 10-9	0.7281	0.654-0.8106
1	210131602	T	0.5016	0.4375	C	1.37 × 10-6	1.294	1.166-1.438	T	0.3637	0.4375	C	1.74 × 10-8	0.7351	0.6604-0.8183
1	210132438	A	0.3714	0.4373	C	5.93 × 10-7	0.7604	0.6828-0.8469	A	0.5156	0.4373	C	3.04 × 10-9	1.369	1.234-1.52
1	210134382	G	0.5016	0.4375	A	1.37 × 10-6	1.294	1.166-1.438	G	0.3637	0.4375	A	1.74 × 10-8	0.7351	0.6604-0.8183
1	210134548	A	0.5016	0.4375	G	1.37 × 10-6	1.294	1.166-1.438	A	0.3637	0.4375	G	1.74 × 10-8	0.7351	0.6604-0.8183
1	210134631	C	0.5016	0.4375	T	1.37 × 10-6	1.294	1.166-1.438	C	0.3637	0.4375	T	1.74 × 10-8	0.7351	0.6604-0.8183
1	210136902	T	0.5016	0.4375	C	1.37 × 10-6	1.294	1.166-1.438	T	0.3637	0.4375	C	1.74 × 10-8	0.7351	0.6604-0.8183
1	210141332	A	0.2571	0.2415	T	0.1752	1.087	0.9636-1.226	A	0.184	0.2415	T	2.29 × 10-7	0.7081	0.6211-0.8074
1	210141333	T	0.3665	0.432	A	6.60 × 10-7	0.7608	0.6829-0.8475	T	0.507	0.432	A	1.29 × 10-8	1.352	1.218-1.5
1	210142978	A	0.3714	0.438	G	4.58 × 10-7	0.7584	0.681-0.8446	A	0.515	0.438	G	5.28 × 10-9	1.363	1.228-1.512
1	210143254	C	0.5016	0.4375	T	1.37 × 10-6	1.294	1.166-1.438	C	0.3637	0.4375	T	1.74 × 10-8	0.7351	0.6604-0.8183
1	210143601	A	0.5016	0.4375	G	1.37 × 10-6	1.294	1.166-1.438	A	0.3637	0.4375	G	1.74 × 10-8	0.7351	0.6604-0.8183
1	210146965	A	0.2571	0.2409	G	0.1572	1.091	0.967-1.23	A	0.1835	0.2409	G	2.37 × 10-7	0.7082	0.621-0.8075
1	210147301	G	0.256	0.2395	T	0.1506	1.092	0.9683-1.232	G	0.1835	0.2395	T	4.28 × 10-7	0.7133	0.6255-0.8135
1	210147382	T	0.3709	0.4381	C	3.45 × 10-7	0.7561	0.6789-0.8421	T	0.5156	0.4381	C	4.46 × 10-9	1.365	1.23-1.515
1	210147560	A	0.3709	0.4381	G	3.45 × 10-7	0.7561	0.6789-0.8421	A	0.5156	0.4381	G	4.46 × 10-9	1.365	1.23-1.515
1	210147622	A	0.3709	0.4381	G	3.45 × 10-7	0.7561	0.6789-0.8421	A	0.5156	0.4381	G	4.46 × 10-9	1.365	1.23-1.515
1	210147988	T	0.3709	0.4385	C	3.03 × 10-7	0.7551	0.678-0.8409	T	0.5156	0.4385	C	5.20 × 10-9	1.363	1.228-1.513
1	210148346	T	0.5016	0.4375	C	1.37 × 10-6	1.294	1.166-1.438	T	0.3637	0.4375	C	1.74 × 10-8	0.7351	0.6604-0.8183
1	210149956	G	0.3704	0.4385	C	2.42 × 10-7	0.7533	0.6764-0.839	G	0.5156	0.4385	C	5.20 × 10-9	1.363	1.228-1.513
1	210149996	A	0.3704	0.4386	G	2.27 × 10-7	0.7528	0.6759-0.8384	A	0.5156	0.4386	G	5.61 × 10-9	1.362	1.227-1.512
1	210150326	G	0.5016	0.4375	A	1.37 × 10-6	1.294	1.166-1.438	G	0.3637	0.4375	A	1.74 × 10-8	0.7351	0.6604-0.8183
1	210150702	T	0.372	0.4396	C	2.98 × 10-7	0.7551	0.678-0.8409	T	0.5156	0.4396	C	8.83 × 10-9	1.357	1.222-1.505

1	210151419	G	0.5016	0.4375	C	1.37 ×10-6	1.294	1.166-1.438	G	0.3637	0.4375	C	1.74 ×10-8	0.7351	0.6604-0.8183
1	210152344	T	0.3687	0.4386	C	1.15 ×10-7	0.7475	0.6711-0.8326	T	0.5156	0.4386	C	5.61 ×10-9	1.362	1.227-1.512
1	210152452	G	0.5016	0.4375	C	1.37 ×10-6	1.294	1.166-1.438	G	0.3637	0.4375	C	1.74 ×10-8	0.7351	0.6604-0.8183
1	210152515	T	0.3682	0.4385	C	9.81 ×10-8	0.7463	0.67-0.8312	T	0.5145	0.4385	C	8.43 ×10-9	1.357	1.223-1.506
1	210154641	G	0.3676	0.4381	C	8.93 ×10-8	0.7455	0.6693-0.8304	G	0.5139	0.4381	C	9.22 ×10-9	1.356	1.222-1.505
1	210155915	G	0.3676	0.438	GCA	9.56 ×10-8	0.746	0.6698-0.831	G	0.5139	0.438	GCA	8.55 ×10-9	1.357	1.223-1.506
1	210155923	C	0.3676	0.4378	T	1.02 ×10-7	0.7465	0.6702-0.8315	C	0.5139	0.4378	T	7.93 ×10-9	1.358	1.224-1.507
1	210156022	C	0.5016	0.4375	T	1.37 ×10-6	1.294	1.166-1.438	C	0.3637	0.4375	T	1.74 ×10-8	0.7351	0.6604-0.8183
1	210156647	T	0.3676	0.4376	C	1.10 ×10-7	0.747	0.6707-0.8321	T	0.5134	0.4376	C	9.35 ×10-9	1.356	1.222-1.505
1	210159278	T	0.3676	0.4376	C	1.10 ×10-7	0.747	0.6707-0.8321	T	0.5129	0.4376	C	1.19 ×10-8	1.353	1.219-1.501
1	210159746	T	0.3676	0.4376	C	1.10 ×10-7	0.747	0.6707-0.8321	T	0.5129	0.4376	C	1.19 ×10-8	1.353	1.219-1.501
1	210159957	C	0.2588	0.242	CAA	0.146	1.093	0.9694-1.233	C	0.1867	0.242	CAA	6.70 ×10-7	0.7189	0.6309-0.8191
1	210160689	G	0.4995	0.4366	A	2.26 ×10-6	1.287	1.159-1.43	G	0.3637	0.4366	A	2.49 ×10-8	0.7376	0.6626-0.8211
1	210160735	G	0.2566	0.2405	T	0.1626	1.09	0.966-1.229	G	0.1835	0.2405	T	2.75 ×10-7	0.7094	0.6221-0.809
1	210161060	G	0.3676	0.4376	A	1.10 ×10-7	0.747	0.6707-0.8321	G	0.5129	0.4376	A	1.19 ×10-8	1.353	1.219-1.501
1	210161061	C	0.3676	0.4376	T	1.10 ×10-7	0.747	0.6707-0.8321	C	0.5129	0.4376	T	1.19 ×10-8	1.353	1.219-1.501
1	210161570	C	0.256	0.2404	T	0.1727	1.087	0.964-1.227	C	0.1835	0.2404	T	2.96 ×10-7	0.7101	0.6227-0.8097
1	210162953	G	0.256	0.2404	A	0.1727	1.087	0.964-1.227	G	0.1835	0.2404	A	2.96 ×10-7	0.7101	0.6227-0.8097
1	210163621	A	0.256	0.2404	G	0.1727	1.087	0.964-1.227	A	0.1835	0.2404	G	2.96 ×10-7	0.7101	0.6227-0.8097
1	210163943	T	0.4989	0.4368	TAA	2.95 ×10-6	1.284	1.156-1.426	T	0.3632	0.4368	TAA	1.83 ×10-8	0.7354	0.6606-0.8187
1	210164099	T	0.3682	0.4375	TA	1.47 ×10-7	0.7493	0.6727-0.8346	T	0.5123	0.4375	TA	1.40 ×10-8	1.351	1.217-1.499
1	210165671	G	0.256	0.2404	A	0.1727	1.087	0.964-1.227	G	0.1835	0.2404	A	2.96 ×10-7	0.7101	0.6227-0.8097
1	210167034	A	0.366	0.4363	G	9.48 ×10-8	0.7458	0.6695-0.8308	A	0.5123	0.4363	G	8.28 ×10-9	1.357	1.223-1.506
1	210167986	A	0.366	0.4356	G	1.24 ×10-7	0.7478	0.6713-0.833	A	0.5123	0.4356	G	6.12 ×10-9	1.361	1.226-1.51
1	210169084	A	0.3654	0.4353	G	1.13 ×10-7	0.747	0.6706-0.8322	A	0.5123	0.4353	G	5.25 ×10-9	1.363	1.228-1.512
1	210169658	G	0.3649	0.4353	A	9.01 ×10-8	0.7453	0.669-0.8303	G	0.5123	0.4353	A	5.25 ×10-9	1.363	1.228-1.512

1	210170230	T	0.2582	0.2405	C	0.1239	1.099	0.9745-1.239	T	0.1845	0.2405	C	4.62 ×10-7	0.7145	0.6267-0.8146
1	210170463	G	0.2588	0.2417	A	0.138	1.095	0.9712-1.235	G	0.1845	0.2417	A	2.77 ×10-7	0.71	0.6228-0.8094
1	210173470	G	0.3638	0.4346	A	7.46 ×10-8	0.7438	0.6676-0.8286	G	0.5123	0.4346	A	3.87 ×10-9	1.367	1.231-1.517
1	210173529	A	0.3638	0.4345	C	7.99 ×10-8	0.7443	0.6681-0.8292	A	0.5123	0.4345	C	3.58 ×10-9	1.368	1.232-1.518
1	210173559	A	0.3638	0.4345	G	7.99 ×10-8	0.7443	0.6681-0.8292	A	0.5123	0.4345	G	3.58 ×10-9	1.368	1.232-1.518
1	210174099	G	0.3638	0.4345	C	7.99 ×10-8	0.7443	0.6681-0.8292	G	0.5113	0.4345	C	5.83 ×10-9	1.362	1.227-1.511
1	210174417	G	0.5	0.437	A	2.09 ×10-6	1.289	1.16-1.431	G	0.3653	0.437	A	4.37 ×10-8	0.7418	0.6664-0.8256
1	210174722	A	0.2593	0.242	G	0.1333	1.096	0.9723-1.236	A	0.1851	0.242	G	3.10 ×10-7	0.7113	0.624-0.8107
1	210174923	T	0.3632	0.434	C	7.79 ×10-8	0.744	0.6678-0.8289	T	0.5113	0.434	C	4.64 ×10-9	1.364	1.229-1.514
1	210174981	T	0.5	0.437	TAAG	2.09 ×10-6	1.289	1.16-1.431	T	0.3659	0.437	TAAG	5.52 ×10-8	0.7435	0.668-0.8275
1	210176554	A	0.5093	0.4476	T	3.54 ×10-6	1.281	1.154-1.423	A	0.3729	0.4476	T	1.23 ×10-8	0.7338	0.6595-0.8164
1	210176904	G	0.5093	0.4476	A	3.54 ×10-6	1.281	1.154-1.423	G	0.3729	0.4476	A	1.23 ×10-8	0.7338	0.6595-0.8164
1	210176923	AT	0.5208	0.4585	A	3.01 ×10-6	1.283	1.156-1.425	AT	0.3852	0.4585	A	2.51 ×10-8	0.7399	0.6653-0.8227
1	210177234	G	0.5093	0.4476	A	3.54 ×10-6	1.281	1.154-1.423	G	0.3729	0.4476	A	1.23 ×10-8	0.7338	0.6595-0.8164
1	210177281	T	0.3523	0.4207	A	1.80 ×10-7	0.749	0.6718-0.835	T	0.4979	0.4207	A	4.46 ×10-9	1.365	1.23-1.515
1	210177973	A	0.3523	0.4204	G	2.05 ×10-7	0.75	0.6727-0.8361	A	0.4973	0.4204	G	4.88 ×10-9	1.364	1.229-1.514
1	210178767	A	0.2691	0.2538	G	0.1891	1.083	0.9616-1.219	A	0.1964	0.2538	G	3.87 ×10-7	0.7183	0.6319-0.8165
1	210180988	A	0.5093	0.4476	T	3.54 ×10-6	1.281	1.154-1.423	A	0.3729	0.4476	T	1.23 ×10-8	0.7338	0.6595-0.8164
1	210181612	T	0.3507	0.4184	G	2.32 ×10-7	0.7507	0.6733-0.837	T	0.4941	0.4184	G	8.36 ×10-9	1.358	1.223-1.507
1	210186038	C	0.5088	0.4472	T	3.82 ×10-6	1.28	1.153-1.421	C	0.3729	0.4472	T	1.43 ×10-8	0.7348	0.6604-0.8175
1	210188154	T	0.2702	0.254	C	0.1639	1.088	0.9663-1.224	T	0.1969	0.254	C	4.64 ×10-7	0.7201	0.6335-0.8185
1	210188353	A	0.5088	0.4472	AT	3.82 ×10-6	1.28	1.153-1.421	A	0.3729	0.4472	AT	1.43 ×10-8	0.7348	0.6604-0.8175
1	210190104	T	0.5088	0.4471	C	3.60 ×10-6	1.281	1.153-1.422	T	0.3723	0.4471	C	1.21 ×10-8	0.7336	0.6593-0.8162
1	210190242	T	0.349	0.4182	C	1.25 ×10-7	0.7458	0.6688-0.8317	T	0.4925	0.4182	C	1.59 ×10-8	1.35	1.216-1.498
1	210190755	C	0.5088	0.4471	CTGCT	3.60 ×10-6	1.281	1.153-1.422	C	0.3723	0.4471	CTGCT	1.21 ×10-8	0.7336	0.6593-0.8162
1	210191645	G	0.3485	0.4177	A	1.22 ×10-7	0.7456	0.6686-0.8314	G	0.493	0.4177	A	9.98 ×10-9	1.356	1.221-1.505

1	210192249	A	0.3479	0.4177	G	9.69 ×10-8	0.7438	0.6669-0.8294	A	0.493	0.4177	G	9.98 ×10-9	1.356	1.221-1.505
1	210194685	G	0.5088	0.4469	A	3.39 ×10-6	1.282	1.154-1.423	G	0.3723	0.4469	A	1.30 ×10-8	0.7341	0.6597-0.8168
1	210195000	C	0.3463	0.4162	T	8.93 ×10-8	0.7429	0.6661-0.8286	C	0.4903	0.4162	T	1.67 ×10-8	1.349	1.216-1.498
1	210195465	T	0.2702	0.2545	C	0.1774	1.085	0.9638-1.221	T	0.1969	0.2545	C	3.74 ×10-7	0.7182	0.6319-0.8163
1	210196010	A	0.5088	0.4469	G	3.39 ×10-6	1.282	1.154-1.423	A	0.3723	0.4469	G	1.30 ×10-8	0.7341	0.6597-0.8168
1	210198888	G	0.3457	0.4171	C	5.00 ×10-8	0.7386	0.6623-0.8238	G	0.492	0.4171	C	1.19 ×10-8	1.353	1.22-1.502
1	210200144	A	0.3457	0.4171	ATTAT	5.00 ×10-8	0.7386	0.6623-0.8238	A	0.492	0.4171	ATTAT	1.19 ×10-8	1.353	1.22-1.502
1	210201065	G	0.3457	0.4167	A	5.75 ×10-8	0.7396	0.6632-0.8249	G	0.492	0.4167	A	1.02 ×10-8	1.355	1.221-1.504
1	210201247	G	0.3457	0.4166	A	6.16 ×10-8	0.7401	0.6636-0.8255	G	0.492	0.4166	A	9.50 ×10-9	1.356	1.222-1.505
1	210201867	T	0.2702	0.2546	C	0.182	1.084	0.9629-1.22	T	0.1964	0.2546	C	2.69 ×10-7	0.7152	0.6291-0.8129
1	210201902	A	0.2702	0.2546	G	0.182	1.084	0.9629-1.22	A	0.1964	0.2546	G	2.69 ×10-7	0.7152	0.6291-0.8129
1	210204005	C	0.3457	0.4164	T	6.60 ×10-8	0.7406	0.6641-0.8261	C	0.492	0.4164	T	8.80 ×10-9	1.357	1.223-1.506
1	210204596	G	0.3457	0.4164	A	6.60 ×10-8	0.7406	0.6641-0.8261	G	0.492	0.4164	A	8.80 ×10-9	1.357	1.223-1.506
1	210205055	G	0.5088	0.4471	C	3.60 ×10-6	1.281	1.153-1.422	G	0.3723	0.4471	C	1.21 ×10-8	0.7336	0.6593-0.8162
1	210205999	T	0.3654	0.432	TA	4.27 ×10-7	0.7572	0.6797-0.8435	T	0.5064	0.432	TA	1.63 ×10-8	1.349	1.216-1.497
1	210206084	C	0.3463	0.4172	CTGT	5.91 ×10-8	0.7399	0.6634-0.8252	C	0.4925	0.4172	CTGT	1.01 ×10-8	1.355	1.221-1.504
1	210206277	AT	0.2702	0.255	A	0.1917	1.082	0.9612-1.218	AT	0.1969	0.255	A	3.01 ×10-7	0.7163	0.6303-0.8142
1	210206353	G	0.3479	0.4167	A	1.46 ×10-7	0.7468	0.6697-0.8328	G	0.493	0.4167	A	6.32 ×10-9	1.361	1.226-1.511
1	210208472	T	0.5088	0.4471	C	3.60 ×10-6	1.281	1.153-1.422	T	0.3718	0.4471	C	9.46 ×10-9	0.7319	0.6578-0.8144
1	210208941	A	0.349	0.4174	G	1.76 ×10-7	0.7484	0.6711-0.8345	A	0.4941	0.4174	G	5.29 ×10-9	1.363	1.228-1.513
1	210209952	G	0.2702	0.2546	C	0.182	1.084	0.9629-1.22	G	0.1969	0.2546	C	3.48 ×10-7	0.7176	0.6314-0.8156
1	210210874	GT	0.3452	0.4149	G	9.70 ×10-8	0.7434	0.6665-0.8292	GT	0.4903	0.4149	G	9.15 ×10-9	1.357	1.222-1.506
1	210211912	A	0.349	0.4189	G	9.55 ×10-8	0.7438	0.667-0.8294	A	0.4989	0.4189	G	1.14 ×10-9	1.381	1.245-1.533
1	210212880	C	0.5088	0.4472	G	3.82 ×10-6	1.28	1.153-1.421	C	0.3723	0.4472	G	1.12 ×10-8	0.7331	0.6589-0.8157
