# Supplementary material for: Genetic factors define CPO and CLO subtypes of nonsyndromicorofacial cleft
Source: PLoS Genet. 2019 Oct 14;15(10):e1008357. doi: 10.1371/journal.pgen.1008357 (PMC6812857; doi:10.1371/journal.pgen.1008357)
Supplement: S4 Table — (PDF) [file pgen.1008357.s012.pdf]

Supplementary Table 4. The regulatory elements in the HaploReg database in the *IRF6* region.

Query SNP:rs72741048 and variants with r2>= 0.4

pos (hg19)	LD (r^2)	LD (D')	variant	Ref	Alt	Promoter histone marks	Enhancer histone marks	DNAse	Proteins bound	eQTL tissues	Motifs changed	Drivers disrupted	GENCODE genes	dbSNP func annot
chr1:209946027	0.41	0.92	rs2235370	C	A	BLD	4 organs				7 altered motifs		TRAF3IP3	intronic
chr1:209947548	0.46	0.97	rs11119345	G	A		BLD, THYM	BRST			Pou2f2		TRAF3IP3	intronic
chr1:209947885	0.44	0.94	rs4140636	T	C		4 organs	BRST,BRST			HNF1		TRAF3IP3	intronic
chr1:209950132	0.44	0.94	rs12058100	C	T		BLD	BRST,BRST			En-1,Nkx2,STAT		TRAF3IP3	intronic
chr1:209951602	0.44	0.94	rs12083685	G	A		BRST, BLD, SKIN	BRST,BRST					TRAF3IP3	intronic
chr1:209951723	0.46	0.97	rs41274834	G	T		BRST, BLD, SKIN	BRST,BRST			GR,HDAC2,p300		TRAF3IP3	intronic
chr1:209951830	0.44	0.94	rs41274836	T	G		BRST, BLD, SKIN	BRST,BRST			7 altered motifs		TRAF3IP3	intronic
chr1:209952266	0.44	0.94	rs12081405	T	A		BRST, BLD, SKIN	BRST,BRST	POL2B		Mef2		TRAF3IP3	intronic
chr1:209958140	0.45	0.69	rs58641937	TGC	T	24 organs		53 organs	26 bound proteins		E2F,GCM,TCF12		235bp 5' of C1orf74	
chr1:209958580	0.45	0.69	rs4844895	T	C	22 organs	12 organs	11 organs			4 altered motifs		455bp 3' of IRF6	
chr1:209959168	0.68	0.98	rs1856161	C	T	BLD, HRT	8 organs	5 organs			Ets,Spdef		IRF6	3'-UTR
chr1:209960436	0.45	0.69	rs2235372	G	A	HRT	14 organs	BRST,SKIN,HRT			CEBPB,Myf,Pax-4		RP3-434O14.8	3'-UTR
chr1:209960925	0.45	0.69	rs742214	T	C	BRN, HRT	19 organs	5 organs			15 altered motifs		RP3-434O14.8	3'-UTR

chr1:209961023	0.45	0.69	rs742215	T	A	BRN, HRT	18 organs	8 organs	FOXA1			RP3-434O14.8	3'-UTR
chr1:209962539	0.45	0.69	rs7522250	T	C		LIV, GI	BRST,BRST,SKIN				IRF6	intronic
chr1:209962794	0.45	0.69	rs2073485	G	A		LIV, GI	4 organs	GATA2	9 altered motifs		IRF6	intronic
chr1:209963803	0.45	0.69	rs2235373	G	A	MUS, LNG	12 organs	6 organs		Ik-1		IRF6	intronic
chr1:209964080	0.68	0.98	rs2235371	C	T	MUS, LNG	13 organs	BRST,BRST,SKIN		CTCF,ERalpha-a		IRF6	missense
chr1:209965283	0.73	0.99	rs926348	G	C		8 organs	BRST,BRST,SKIN	POL2B	Ets,STAT,p53		IRF6	intronic
chr1:209965587	0.73	0.99	rs2235375	G	C		10 organs	BRST,SKIN,SKIN	STAT3			IRF6	intronic
chr1:209966629	0.68	0.98	rs17015217	G	A		BRST	BRST		4 altered motifs		IRF6	intronic
chr1:209967380	0.73	-0.99	rs2179254	T	C		BRST	4 organs	POL2B	Gcm1,Zfp187		IRF6	intronic
chr1:209968319	0.73	-0.99	rs6685182	A	C		BRST	BRST,BRST,SKIN		AP-1,AP-2,Elf5		IRF6	intronic
chr1:209968684	0.73	0.99	rs2013162	C	A		SKIN, GI, BRST	4 organs	POL2B	Foxp3,NRSF		IRF6	synonymous
chr1:209970610	0.62	0.86	rs59043219	G	A		BRST, GI			BDP1,LUN-1		IRF6	intronic
chr1:209971628	0.73	0.99	rs2236907	C	A	ESC	11 organs	8 organs	CTCF,RAD21			IRF6	intronic
chr1:209971640	0.73	0.99	rs2236908	G	C	ESC	11 organs	7 organs	CTCF,RAD21	LRH1		IRF6	intronic
chr1:209971655	0.73	0.99	rs2236909	A	G	ESC	11 organs	7 organs	CTCF,RAD21	6 altered motifs		IRF6	intronic
chr1:209972075	0.66	-0.91	rs2005982	G	A		BRST			NF-kappaB		IRF6	intronic
chr1:209972198	0.68	0.98	rs4844494	G	A		BRST			NF-I,TLX1::NFIC,YY1		IRF6	intronic
chr1:209973549	0.73	0.99	rs2294408	G	A	LIV	BRST, SKIN	SKIN,BRST,SKIN				IRF6	intronic
chr1:209973922	0.64	0.88	rs1160411	T	C	LIV	BRST, SKIN	BRST,BRST		4 altered motifs		IRF6	intronic
chr1:209975392	0.68	0.98	rs2235377	T	C	9 organs	16 organs	6 organs	POL2B,POL2			IRF6	intronic
chr1:209976215	0.74	0.99	rs2073486	G	A	6 organs	9 organs	4 organs		4 altered motifs		IRF6	intronic
chr1:209976646	0.74	0.99	rs2073487	T	C	7 organs	6 organs	BRST,SKIN,BRST	GATA1	Bcl6b,HMG-IY		IRF6	intronic

chr1:209977844	0.68	0.98	rs12405750	C	T	15 organs	BRST, SKIN, PLCNT	4 organs	POL2B	Gfi1,Mef2,Pou5f1	IRF6	intronic
chr1:209978098	0.68	0.98	rs3753517	C	T	18 organs	BRST, SKIN, BLD	BRST,SKIN,GI		Foxf1,Foxi1,Pou1f1	IRF6	intronic
chr1:209978777	0.74	0.99	rs17015250	T	G	23 organs	BLD	12 organs		Irf,Smad,TAL1	IRF6	intronic
chr1:209979014	0.74	0.99	rs12403599	G	C	24 organs	MUS, BLD	14 organs	CTCF,RAD21		IRF6	intronic
chr1:209979613	0.74	0.99	rs7545538	C	G	24 organs		46 organs	16 bound proteins	10 altered motifs	147bp 5' of IRF6	
chr1:209979635	0.74	0.99	rs7545542	C	T	24 organs		43 organs	16 bound proteins	HNF4	169bp 5' of IRF6	
chr1:209979997	0.73	0.98	rs151296501	9-mer	A	24 organs		19 organs		4 altered motifs	531bp 5' of IRF6	
chr1:209980155	0.74	0.99	rs6659549	T	G	18 organs	14 organs	BRST,SKIN			689bp 5' of IRF6	
chr1:209980489	0.72	0.98	rs2357229	G	T	BRST, SKIN, MUS	13 organs	BRST,BRST		6 altered motifs	1kb 5' of IRF6	
chr1:209980757	0.74	0.99	rs1005287	G	A			4 organs			1.3kb 5' of IRF6	
chr1:209982025	0.45	0.69	rs6540559	G	A		17 organs	SKIN		4 altered motifs	2.6kb 5' of IRF6	
chr1:209982372	0.45	0.69	rs6696825	A	G		12 organs			AP-1,ERalpha-a,NRSF	2.9kb 5' of IRF6	
chr1:209982738	0.69	0.99	rs17015255	A	G		11 organs	BRST,SKIN,BRST		CDP,Sin3Ak-20	3.3kb 5' of IRF6	
chr1:209982923	0.45	0.69	rs17015259	G	A		12 organs	5 organs			3.5kb 5' of IRF6	
chr1:209983331	0.45	0.69	rs764093	A	G		14 organs	5 organs		BDP1,GR,RFX5	3.9kb 5' of IRF6	

chr1:209983900	0.7	1	rs11119346	C	T	4 organs			PLZF,Pou5f1,SRF	4.4kb 5' of IRF6
chr1:209983916	0.7	1	rs11119347	T	C	4 organs			PLZF	4.5kb 5' of IRF6
chr1:209984013	0.7	1	rs2069068	G	T	5 organs	ESC		AIRE,Nr2e3,Nr2f2	4.5kb 5' of IRF6
chr1:209984470	0.7	1	rs75477785	T	G	BRST, SKIN		GATA2	EBF,GR,Hsf	5kb 5' of IRF6
chr1:209984824	0.7	1	rs10489343	G	T				Bcl6b,Foxo,Pou1f1	5.4kb 5' of IRF6
chr1:209985136	0.7	1	rs77037440	C	T	BRST, SKIN				5.7kb 5' of IRF6
chr1:209985951	0.62	1	rs12064398	A	C	BRST, SKIN			Zfp691	6.5kb 5' of IRF6
chr1:209985952	0.62	1	rs12069187	G	T	BRST, SKIN			4 altered motifs	6.5kb 5' of IRF6
chr1:209986054	0.7	1	rs150541980	GTGAC	G	BRST, SKIN			Ets,Ik-1	6.6kb 5' of IRF6
chr1:209986747	0.7	1	rs12566152	C	T	BRST, SKIN				7.3kb 5' of IRF6
chr1:209987624	0.7	1	rs10779514	C	T			TCF4	AP-1,BHLHE40,Maf	8.2kb 5' of IRF6
chr1:209987632	0.68	0.98	rs10779515	G	A				BHLHE40,Hand1,Smad	8.2kb 5' of IRF6
chr1:209987712	0.7	1	rs35431945	AT	A	BRST			13 altered motifs	8.2kb 5' of IRF6
chr1:209987919	0.7	1	rs10863789	T	C				Evi-1,Ik-2	8.5kb 5' of IRF6
chr1:209988047	0.7	1	rs10863790	A	C		IPSC			8.6kb 5' of IRF6
chr1:209988274	0.7	1	rs79384526	A	G	8 organs	BRST		Pou3f3	8.8kb 5' of IRF6

chr1:209988527	0.7	1	rs77249837	G	A		10 organs			HNF1,Pax-6		9.1kb 5' of IRF6
chr1:209988809	0.7	1	rs75889558	G	T		12 organs	BRST,SKIN,BRST		Nkx3,Pou3f2,Pou3f4		9.3kb 5' of IRF6
chr1:209988989	0.7	1	rs17015268	G	A		12 organs	8 organs	NFKB,STAT3	PLZF,TCF4		9.5kb 5' of IRF6
chr1:209989092	1	1	rs72741048	A	T	BLD, PANC, SKIN	16 organs	4 organs	NFKB,STAT3	6 altered motifs		9.6kb 5' of IRF6
chr1:209989232	0.7	1	rs76145088	A	G	6 organs	20 organs	5 organs	POL2,POL2B	4 altered motifs		9.8kb 5' of IRF6
chr1:209989281	0.7	1	rs77542756	G	A	6 organs	20 organs	5 organs	POL2,POL2B	RP58		9.8kb 5' of IRF6
chr1:209989729	0.7	1	rs11119348	A	C	5 organs	21 organs	6 organs	POL2B	9 altered motifs		10kb 5' of IRF6
chr1:209992127	0.45	0.69	rs12070337	G	A					Irf,Mef2		9.2kb 5' of DIEXF
chr1:209992501	0.69	0.99	rs1109430	G	A		BRST, GI	ESDR,GI				8.9kb 5' of DIEXF
chr1:209994715	0.46	-1	rs598514	C	T		BRST, SKIN	SKIN		7 altered motifs		6.6kb 5' of DIEXF
chr1:209995470	0.69	0.99	rs12075674	G	A		SKIN			Pou2f2,Pou3f2		5.9kb 5' of DIEXF
chr1:209996542	0.43	-0.97	rs9430018	T	G	16 organs	19 organs	30 organs	4 bound proteins			4.8kb 5' of DIEXF
chr1:209997123	0.43	-0.95	rs5780539	CA	C	16 organs	21 organs	8 organs	4 bound proteins	Bbx,Hbp1,Pou5f1	HBP1_1,Enh_c159	4.2kb 5' of DIEXF
chr1:209998053	0.43	-0.96	rs616702	A	G		11 organs			EWSR1-FLI1,Pou1f1		3.3kb 5' of DIEXF
chr1:209999030	0.43	-0.96	rs201011609	G	7-mer		19 organs	4 organs		7 altered motifs		2.3kb 5' of DIEXF
chr1:209999051	0.43	-0.96	rs12731696	G	T		19 organs	4 organs		5 altered motifs		2.3kb 5' of DIEXF

chr1:209999630	0.43	-0.96	rs3766616	A	G	MUS, BRST	17 organs	10 organs	JUND	SRF,TAL1		1.7kb 5' of DIEXF	
chr1:210000148	0.43	-0.96	rs646596	T	C	BLD	13 organs			PRDM1		1.2kb 5' of DIEXF	
chr1:210000693	0.43	-0.96	rs659611	C	A	13 organs	15 organs			HNF4,SETDB1,Znf143		658bp 5' of DIEXF	
chr1:210001048	0.43	-0.96	rs660975	A	C	24 organs		36 organs	8 bound proteins	NF-kappaB,Zbtb3		303bp 5' of DIEXF	
chr1:210001125	0.43	-0.96	rs3215480	GC	G	24 organs		48 organs	25 bound proteins	AhR,Pax-4		226bp 5' of DIEXF	
chr1:210001586	0.43	-0.96	rs616227	A	G	24 organs		42 organs	CTCF	GATA,Ik-1		DIEXF	intronic
chr1:210001713	0.43	-0.96	rs674346	C	A	24 organs		27 organs	CTCF,EGR1	11 altered motifs	ELF3_1,Pro_c33	DIEXF	intronic
chr1:210002353	0.43	-0.96	rs10863792	G	A					5 altered motifs		DIEXF	intronic
chr1:210007416	0.64	0.99	rs12080691	A	T					Fox,Foxa,Foxp1		DIEXF	intronic
chr1:210009372	0.42	-0.95	rs592164	G	A		11 organs	6 organs	CFOS,JUND	RXRA,TAL1		DIEXF	intronic
chr1:210022901	0.62	0.98	rs4329516	C	T		BRST, SKIN			Myc		DIEXF	intronic
chr1:210030755	0.46	0.85	rs4844496	T	G					Irf,PPAR		5.2kb 3' of DIEXF	3'-UTR
chr1:210042077	0.43	0.84	rs78355191	C	T		FAT, BRN, SKIN			Foxk1,Foxo,SIX5		17kb 3' of DIEXF	
chr1:210046786	0.42	0.83	rs1883332	G	A	5 organs	16 organs	10 organs	STAT3	Pou3f3		21kb 3' of DIEXF	
chr1:210049893	0.42	0.83	rs12063989	T	C		11 organs	SKIN		6 altered motifs		24kb 3' of DIEXF	
chr1:210049901	0.42	0.83	rs12078157	C	T		11 organs	SKIN		6 altered motifs		24kb 3' of DIEXF	
chr1:210050077	0.42	0.83	rs12064085	T	A		10 organs	SKIN				25kb 3' of DIEXF	
chr1:210050518	0.42	0.83	rs12064211	A	C		10 organs	CRVX	8 bound proteins	Pax-5		25kb 3' of DIEXF	

chr1:210051454	0.42	0.83	rs12062600	G	A		SKIN			Cdc5,Mef2,Sox		26kb 3' of DIEXF
chr1:210051748	0.41	0.82	rs150128380	AAATT	A		SKIN			14 altered motifs		26kb 3' of DIEXF
chr1:210054211	0.41	0.82	rs12563527	G	A		MUS, BRN, SKIN			8 altered motifs		29kb 3' of DIEXF
chr1:210054435	0.41	0.82	rs11119359	T	C		MUS, BRN, SKIN			LBP-1		29kb 3' of DIEXF
chr1:210054718	0.41	0.82	rs4844908	C	T		BRN, SKIN		MAFK			29kb 3' of DIEXF
chr1:210096995	0.42	-0.67	rs11119368	A	C					GR,VDR		15kb 5' of SYT14
