# Supplementary material for: Genetic factors define CPO and CLO subtypes of nonsyndromicorofacial cleft
Source: PLoS Genet. 2019 Oct 14;15(10):e1008357. doi: 10.1371/journal.pgen.1008357 (PMC6812857; doi:10.1371/journal.pgen.1008357)
Supplement: S5 Table — (PDF) [file pgen.1008357.s013.pdf]

Supplementary Table 5. Candidate genes for NSOFC.

Phenotype	Genes	Source type	Reference or database
Cleft palate only	*POMGNT2*; *WHSC1*; *MSX1*; *DOCK9*; *PAX9*; *FOXC2*; *MAU2*; *IRF6*	Susceptibility loci	The presentstudy
cleft lip only	GRM5	suscepbility loci	this study
cleft lip only	ALX1	suscepbility loci	this study
cleft lip only	DLK1	suscepbility loci	this study
cleft lip only	MYCN	suscepbility loci	this study
cleft lip only	VAX1	suscepbility loci	this study
cleft lip only	MAFB	suscepbility loci	this study
cleft lip only	IRF6	suscepbility loci	this study
cleft lip and palate	GRM5	suscepbility loci	this study
cleft lip and palate	IRF6	suscepbility loci	this study
cleft lip and palate	MYCN	suscepbility loci	this study
cleft lip and palate	VAX1	suscepbility loci	this study
cleft lip and palate	MAFB	suscepbility loci	this study
causal role in cleft lip and palate	MTHFR	mutation	Mosseyet al., 2009
causal role in cleft lip and palate	TGFA	mutation	Cleft lip and palate Peter A Mossey, Julian Little et al, 2009
causal role in cleft lip and palate	D4S175	mutation	Cleft lip and palate Peter A Mossey, Julian Little et al, 2009
causal role in cleft lip and palate	F13A1	mutation	Cleft lip and palate Peter A Mossey, Julian Little et al, 2009
causal role in cleft lip and palate	TGFB3	mutation	Cleft lip and palate Peter A Mossey, Julian Little et al, 2009
causal role in cleft lip and palate	D17S250	mutation	Cleft lip and palate Peter A Mossey, Julian Little et al, 2009
causal role in cleft lip and palate	APOC2	mutation	Cleft lip and palate Peter A Mossey, Julian Little et al, 2009
palate development	Osr2	development	Cleft lip and palate Peter A Mossey, Julian Little et al, 2009
palate development	Lhx8	development	Cleft lip and palate Peter A Mossey, Julian Little et al, 2009
palate development	Msx1	development	Cleft lip and palate Peter A Mossey, Julian Little et al, 2009
palate development	Fgf10	development	Cleft lip and palate Peter A Mossey, Julian Little et al, 2009
palate development	Fgfr2b	development	Cleft lip and palate Peter A Mossey, Julian Little et al, 2009
palate development	Tgfb2	development	Cleft lip and palate Peter A Mossey, Julian Little et al, 2009
palate development	Tgfbr2	development	Cleft lip and palate Peter A Mossey, Julian Little et al, 2009
palate development	FGF10	development	Cleft lip and palate Peter A Mossey, Julian Little et al, 2009

palate development	FGFR2b	development	Cleft lip and palate Peter A Mossey, Julian Little et al, 2009
palate development	Msx1	development	Cleft lip and palate Peter A Mossey, Julian Little et al, 2009
palate development	Bmp4	development	Cleft lip and palate Peter A Mossey, Julian Little et al, 2009
palate development	Shh	development	Cleft lip and palate Peter A Mossey, Julian Little et al, 2009
palate development	Bmp2	development	Cleft lip and palate Peter A Mossey, Julian Little et al, 2009
palate development	Msx1	development	Cleft lip and palate Peter A Mossey, Julian Little et al, 2009
palate development	Bmp2	development	Cleft lip and palate Peter A Mossey, Julian Little et al, 2009
palate development	Bmp4	development	Cleft lip and palate Peter A Mossey, Julian Little et al, 2009
palate development	Shh	development	Cleft lip and palate Peter A Mossey, Julian Little et al, 2009
palate development	Bmp4	development	Cleft lip and palate Peter A Mossey, Julian Little et al, 2009
palate development	JAG2	development	Cleft lip and palate Peter A Mossey, Julian Little et al, 2009
palate development	TGFA	development	Cleft lip and palate Peter A Mossey, Julian Little et al, 2009
palate development	EGFR	development	Cleft lip and palate Peter A Mossey, Julian Little et al, 2009
palate development	TGFβ3	development	Cleft lip and palate Peter A Mossey, Julian Little et al, 2009
palate development	TIMP2	development	Cleft lip and palate Peter A Mossey, Julian Little et al, 2009
palate development	MMP13	development	Cleft lip and palate Peter A Mossey, Julian Little et al, 2009
palate development	SHH	development	Cleft lip and palate Peter A Mossey, Julian Little et al, 2009
palate development	MSX1	development	Cleft lip and palate Peter A Mossey, Julian Little et al, 2009
palate development	MSX2	development	Cleft lip and palate Peter A Mossey, Julian Little et al, 2009
Cleft palate, non-syndromic	ACACB	mutation	HGMD
Cleft lip with or without cleft palate	ARHGAP29	mutation	HGMD
Cleft palate	ARHGAP29	mutation	HGMD
Cleft palate, non-syndromic	ARID5B	mutation	HGMD
Cleft palate	FOXE1	mutation	HGMD
Cleft palate, non-syndromic	GRHL3	mutation	HGMD
Cleft palate with no lip pits	IRF6	mutation	HGMD
Cleft palate	MAFB	mutation	HGMD
Cleft lip and/or palate	ABCA12	mutation	HGMD
Cleft lip and palate, non-syndromic	ACSS2	mutation	HGMD
Cleft lip and/or palate	AHDC1	mutation	HGMD
Cleft lip and palate, with enamel defects	AMELX	mutation	HGMD
Cleft lip and palate, non-syndromic	ARHGAP29	mutation	HGMD
Cleft lip and palate, non-syndromic ?	ARHGAP29	mutation	HGMD

Cleft lip, non-syndromic	ARHGAP29	mutation	HGMD
Cleft lip with or without cleft palate	ARHGAP29	mutation	HGMD
Cleft lip	ARHGAP29	mutation	HGMD
Cleft lip and palate	BMP4	mutation	HGMD
Cleft lip and palate, non-syndromic	ACSS2	mutation	HGMD
Cleft lip and palate, with enamel defects	AMELX	mutation	HGMD
Cleft lip and palate, non-syndromic	ARHGAP29	mutation	HGMD
Cleft lip and palate, non-syndromic ?	ARHGAP29	mutation	HGMD
Cleft lip and palate	BMP4	mutation	HGMD
Cleft lip and palate, incomplete, association with	CRISPLD2	mutation	HGMD
Cleft lip and palate	FGF8	mutation	HGMD
Cleft lip and palate	FGFR1	mutation	HGMD
Cleft lip and palate	FGFR2	mutation	HGMD
cleft lip ±cleft palate	ACTB	genetic	Dixonet al., 2011
cleft lip ±cleft palate	CDH1	genetic	Cleft lip and palate: understanding genetic and environmental influences Michael J. Dixonet al, 2011
cleft lip ±cleft palate	EFNB1	genetic	Cleft lip and palate: understanding genetic and environmental influences Michael J. Dixonet al, 2011
cleft lip ±cleft palate	ESCO2	genetic	Cleft lip and palate: understanding genetic and environmental influences Michael J. Dixonet al, 2011
cleft lip ±cleft palate	GLI2	genetic	Cleft lip and palate: understanding genetic and environmental influences Michael J. Dixonet al, 2011
cleft lip ±cleft palate	GLI3	genetic	Cleft lip and palate: understanding genetic and environmental influences Michael J. Dixonet al, 2011
cleft lip ±cleft palate	HYLS1	genetic	Cleft lip and palate: understanding genetic and environmental influences Michael J. Dixonet al, 2011
cleft lip ±cleft palate	IRF6	genetic	Cleft lip and palate: understanding genetic and environmental influences Michael J. Dixonet al, 2011
cleft lip ±cleft palate	PHF8	genetic	Cleft lip and palate: understanding genetic and environmental influences Michael J. Dixonet al, 2011
cleft lip ±cleft palate	PTCH1	genetic	Cleft lip and palate: understanding genetic and environmental influences Michael J. Dixonet al, 2011
cleft lip ±cleft palate	PVRL1	genetic	Cleft lip and palate: understanding genetic and environmental influences Michael J. Dixonet al, 2011
cleft lip ±cleft palate	SHH	genetic	Cleft lip and palate: understanding genetic and environmental influences Michael J. Dixonet al, 2011
cleft lip ±cleft palate	SIX3	genetic	Cleft lip and palate: understanding genetic and environmental influences Michael J. Dixonet al, 2011
cleft lip ±cleft palate	TFAP2A	genetic	Cleft lip and palate: understanding genetic and environmental influences Michael J. Dixonet al, 2011
cleft lip ±cleft palate	TGIF1	genetic	Cleft lip and palate: understanding genetic and environmental influences Michael J. Dixonet al, 2011
cleft lip ±cleft palate	TP63	genetic	Cleft lip and palate: understanding genetic and environmental influences Michael J. Dixonet al, 2011
cleft lip ±cleft palate	TP63	genetic	Cleft lip and palate: understanding genetic and environmental influences Michael J. Dixonet al, 2011
cleft lip ±cleft palate	WNT3	genetic	Cleft lip and palate: understanding genetic and environmental influences Michael J. Dixonet al, 2011
cleft palate only	BCOR	genetic	Cleft lip and palate: understanding genetic and environmental influences Michael J. Dixonet al, 2011
cleft palate only	CHD7	genetic	Cleft lip and palate: understanding genetic and environmental influences Michael J. Dixonet al, 2011

cleft palate only	CHRNG	genetic	Cleft lip and palate: understanding genetic and environmental influences Michael J. Dixonet al, 2011
cleft palate only	COL11A1	genetic	Cleft lip and palate: understanding genetic and environmental influences Michael J. Dixonet al, 2011
cleft palate only	COL11A2	genetic	Cleft lip and palate: understanding genetic and environmental influences Michael J. Dixonet al, 2011
cleft palate only	COL2A1	genetic	Cleft lip and palate: understanding genetic and environmental influences Michael J. Dixonet al, 2011
cleft palate only	DHCR24	genetic	Cleft lip and palate: understanding genetic and environmental influences Michael J. Dixonet al, 2011
cleft palate only	DHCR7	genetic	Cleft lip and palate: understanding genetic and environmental influences Michael J. Dixonet al, 2011
cleft palate only	DHODH	genetic	Cleft lip and palate: understanding genetic and environmental influences Michael J. Dixonet al, 2011
cleft palate only	EFNB1	genetic	Cleft lip and palate: understanding genetic and environmental influences Michael J. Dixonet al, 2011
cleft palate only	FGFR1	genetic	Cleft lip and palate: understanding genetic and environmental influences Michael J. Dixonet al, 2011
cleft palate only	FGFR2	genetic	Cleft lip and palate: understanding genetic and environmental influences Michael J. Dixonet al, 2011
cleft palate only	FGFR2	genetic	Cleft lip and palate: understanding genetic and environmental influences Michael J. Dixonet al, 2011
cleft palate only	FLNA	genetic	Cleft lip and palate: understanding genetic and environmental influences Michael J. Dixonet al, 2011
cleft palate only	FLNB	genetic	Cleft lip and palate: understanding genetic and environmental influences Michael J. Dixonet al, 2011
cleft palate only	FOXC2	genetic	Cleft lip and palate: understanding genetic and environmental influences Michael J. Dixonet al, 2011
cleft palate only	FOXE1	genetic	Cleft lip and palate: understanding genetic and environmental influences Michael J. Dixonet al, 2011
cleft palate only	GLI3	genetic	Cleft lip and palate: understanding genetic and environmental influences Michael J. Dixonet al, 2011
cleft palate only	IRF6	genetic	Cleft lip and palate: understanding genetic and environmental influences Michael J. Dixonet al, 2011
cleft palate only	KCNJ2	genetic	Cleft lip and palate: understanding genetic and environmental influences Michael J. Dixonet al, 2011
cleft palate only	MLL2	genetic	Cleft lip and palate: understanding genetic and environmental influences Michael J. Dixonet al, 2011
cleft palate only	NIPBL	genetic	Cleft lip and palate: understanding genetic and environmental influences Michael J. Dixonet al, 2011
cleft palate only	PQBP1	genetic	Cleft lip and palate: understanding genetic and environmental influences Michael J. Dixonet al, 2011
cleft palate only	SATB2	genetic	Cleft lip and palate: understanding genetic and environmental influences Michael J. Dixonet al, 2011
cleft palate only	SLC26A2	genetic	Cleft lip and palate: understanding genetic and environmental influences Michael J. Dixonet al, 2011
cleft palate only	SOX9	genetic	Cleft lip and palate: understanding genetic and environmental influences Michael J. Dixonet al, 2011
cleft palate only	SOX9	genetic	Cleft lip and palate: understanding genetic and environmental influences Michael J. Dixonet al, 2011
cleft palate only	TBX1	genetic	Cleft lip and palate: understanding genetic and environmental influences Michael J. Dixonet al, 2011
cleft palate only	TBX22	genetic	Cleft lip and palate: understanding genetic and environmental influences Michael J. Dixonet al, 2011
cleft palate only	TCOF1	genetic	Cleft lip and palate: understanding genetic and environmental influences Michael J. Dixonet al, 2011
cleft palate only	TGFBR1	genetic	Cleft lip and palate: understanding genetic and environmental influences Michael J. Dixonet al, 2011
cleft palate only	TGFBR2	genetic	Cleft lip and palate: understanding genetic and environmental influences Michael J. Dixonet al, 2011
cleft palate only	TWIST1	genetic	Cleft lip and palate: understanding genetic and environmental influences Michael J. Dixonet al, 2011
Midline cleft lip	MID1	genetic	Cleft lip and palate: understanding genetic and environmental influences Michael J. Dixonet al, 2011
Midline cleft lip	OFD1	genetic	Cleft lip and palate: understanding genetic and environmental influences Michael J. Dixonet al, 2011

Nonsyndromic cleft lip with or without cleft palate	FGFR1	genetic	Leslie & Marazita, 2013
Nonsyndromic cleft lip with or without cleft palate	GLI2	genetic	Genetics of Cleft Lip and Cleft Palate Elizabeth J. Leslie et al., 2013
Nonsyndromic cleft lip with or without cleft palate	GLI3	genetic	Genetics of Cleft Lip and Cleft Palate Elizabeth J. Leslie et al., 2013
Nonsyndromic cleft lip with or without cleft palate	IRF6	genetic	Genetics of Cleft Lip and Cleft Palate Elizabeth J. Leslie et al., 2013
Nonsyndromic cleft lip with or without cleft palate	IRF6	genetic	Genetics of Cleft Lip and Cleft Palate Elizabeth J. Leslie et al., 2013
Nonsyndromic cleft lip with or without cleft palate	MID1	genetic	Genetics of Cleft Lip and Cleft Palate Elizabeth J. Leslie et al., 2013
Nonsyndromic cleft lip with or without cleft palate	MLL2	genetic	Genetics of Cleft Lip and Cleft Palate Elizabeth J. Leslie et al., 2013
Nonsyndromic cleft lip with or without cleft palate	KDM6A	genetic	Genetics of Cleft Lip and Cleft Palate Elizabeth J. Leslie et al., 2013
Nonsyndromic cleft lip with or without cleft palate	MSX1	genetic	Genetics of Cleft Lip and Cleft Palate Elizabeth J. Leslie et al., 2013
Nonsyndromic cleft lip with or without cleft palate	OFD1	genetic	Genetics of Cleft Lip and Cleft Palate Elizabeth J. Leslie et al., 2013
Nonsyndromic cleft lip with or without cleft palate	PHF8	genetic	Genetics of Cleft Lip and Cleft Palate Elizabeth J. Leslie et al., 2013
Nonsyndromic cleft lip with or without cleft palate	PTCH1	genetic	Genetics of Cleft Lip and Cleft Palate Elizabeth J. Leslie et al., 2013
Nonsyndromic cleft lip with or without cleft palate	PVRL1	genetic	Genetics of Cleft Lip and Cleft Palate Elizabeth J. Leslie et al., 2013
Nonsyndromic cleft lip with or without cleft palate	RIPK4	genetic	Genetics of Cleft Lip and Cleft Palate Elizabeth J. Leslie et al., 2013
Nonsyndromic cleft lip with or without cleft palate	SHH	genetic	Genetics of Cleft Lip and Cleft Palate Elizabeth J. Leslie et al., 2013
Nonsyndromic cleft lip with or without cleft palate	SIX3	genetic	Genetics of Cleft Lip and Cleft Palate Elizabeth J. Leslie et al., 2013
Nonsyndromic cleft lip with or without cleft palate	TFAP2A	genetic	Genetics of Cleft Lip and Cleft Palate Elizabeth J. Leslie et al., 2013
Nonsyndromic cleft lip with or without cleft palate	TGIF	genetic	Genetics of Cleft Lip and Cleft Palate Elizabeth J. Leslie et al., 2013
Nonsyndromic cleft lip with or without cleft palate	TP63	genetic	Genetics of Cleft Lip and Cleft Palate Elizabeth J. Leslie et al., 2013
Nonsyndromic cleft lip with or without cleft palate	TP63	genetic	Genetics of Cleft Lip and Cleft Palate Elizabeth J. Leslie et al., 2013
cleft lip with and cleft palate	CDH1	genetic	Genetics of Cleft Lip and Cleft Palate Elizabeth J. Leslie et al., 2013
cleft lip with and cleft palate	WNT3	genetic	Genetics of Cleft Lip and Cleft Palate Elizabeth J. Leslie et al., 2013
Nonsyndromic cleft lip with or without cleft palate	CDH1	genetic	Genetics of Cleft Lip and Cleft Palate Elizabeth J. Leslie et al., 2013
Nonsyndromic cleft lip with or without cleft palate	WNT3	genetic	Genetics of Cleft Lip and Cleft Palate Elizabeth J. Leslie et al., 2013
Cleft palate	BCOR	genetic	Genetics of Cleft Lip and Cleft Palate Elizabeth J. Leslie et al., 2013
Cleft palate	CHD7	genetic	Genetics of Cleft Lip and Cleft Palate Elizabeth J. Leslie et al., 2013
Cleft palate	CHRNG	genetic	Genetics of Cleft Lip and Cleft Palate Elizabeth J. Leslie et al., 2013
Cleft palate	COL11A1	genetic	Genetics of Cleft Lip and Cleft Palate Elizabeth J. Leslie et al., 2013
Cleft palate	COL11A2	genetic	Genetics of Cleft Lip and Cleft Palate Elizabeth J. Leslie et al., 2013
Cleft palate	COL2A1	genetic	Genetics of Cleft Lip and Cleft Palate Elizabeth J. Leslie et al., 2013
Cleft palate	DHODH	genetic	Genetics of Cleft Lip and Cleft Palate Elizabeth J. Leslie et al., 2013
Cleft palate	FGFR2	genetic	Genetics of Cleft Lip and Cleft Palate Elizabeth J. Leslie et al., 2013
Cleft palate	FGFR2	genetic	Genetics of Cleft Lip and Cleft Palate Elizabeth J. Leslie et al., 2013

Cleft palate	FLNA	genetic	Genetics of Cleft Lip and Cleft Palate Elizabeth J. Leslie et al., 2013
Cleft palate	FOXE1	genetic	Genetics of Cleft Lip and Cleft Palate Elizabeth J. Leslie et al., 2013
Cleft palate	NIPBL	genetic	Genetics of Cleft Lip and Cleft Palate Elizabeth J. Leslie et al., 2013
Cleft palate	SATB2	genetic	Genetics of Cleft Lip and Cleft Palate Elizabeth J. Leslie et al., 2013
Cleft palate	SOX9	genetic	Genetics of Cleft Lip and Cleft Palate Elizabeth J. Leslie et al., 2013
Cleft palate	SOX9	genetic	Genetics of Cleft Lip and Cleft Palate Elizabeth J. Leslie et al., 2013
Cleft palate	TBX1	genetic	Genetics of Cleft Lip and Cleft Palate Elizabeth J. Leslie et al., 2013
Cleft palate	TBX22	genetic	Genetics of Cleft Lip and Cleft Palate Elizabeth J. Leslie et al., 2013
Cleft palate	TCOF1	genetic	Genetics of Cleft Lip and Cleft Palate Elizabeth J. Leslie et al., 2013
Cleft palate	TGFBR1	genetic	Genetics of Cleft Lip and Cleft Palate Elizabeth J. Leslie et al., 2013
Cleft palate	TWIST1	genetic	Genetics of Cleft Lip and Cleft Palate Elizabeth J. Leslie et al., 2013
Cleft palate	TGFBR2	genetic	Genetics of Cleft Lip and Cleft Palate Elizabeth J. Leslie et al., 2013
Cleft lip	intergenic	suscepbility loci	GWAS catalog
Cleft lip	IRF6	suscepbility loci	GWAS catalog
Nonsyndromic cleft lip with or without cleft palate	IRF6	suscepbility loci	GWAS catalog
Nonsyndromic cleft lip with or without cleft palate	VAX1	suscepbility loci	GWAS catalog
Nonsyndromic cleft lip with or without cleft palate	CREBBP, ADCY9	suscepbility loci	GWAS catalog
Nonsyndromic cleft lip with or without cleft palate	NTN1	suscepbility loci	GWAS catalog
Nonsyndromic cleft lip with or without cleft palate	MAFB	suscepbility loci	GWAS catalog
Nonsyndromic cleft lip with cleft palate	FAM49A	suscepbility loci	GWAS catalog
Nonsyndromic cleft lip with cleft palate	IRF6, DIEXF	suscepbility loci	GWAS catalog
Nonsyndromic cleft lip with cleft palate	ABCA4	suscepbility loci	GWAS catalog
Nonsyndromic cleft lip with cleft palate	COL8A1	suscepbility loci	GWAS catalog
Nonsyndromic cleft lip with cleft palate	DCAF4L2	suscepbility loci	GWAS catalog
Nonsyndromic cleft lip with cleft palate	VAX1	suscepbility loci	GWAS catalog
Nonsyndromic cleft lip with cleft palate	TMEM19	suscepbility loci	GWAS catalog
Nonsyndromic cleft lip with cleft palate	COL8A1	suscepbility loci	GWAS catalog
Nonsyndromic cleft lip with cleft palate	KIAA1429	suscepbility loci	GWAS catalog
Nonsyndromic cleft lip with cleft palate	PTCH1	suscepbility loci	GWAS catalog
Nonsyndromic cleft lip with cleft palate	NTN1	suscepbility loci	GWAS catalog
Nonsyndromic cleft lip with cleft palate	TAF1B	suscepbility loci	GWAS catalog
Nonsyndromic cleft lip with cleft palate	LOC100506207	suscepbility loci	GWAS catalog
Nonsyndromic cleft lip with cleft palate	RAD54B	suscepbility loci	GWAS catalog

Nonsyndromic cleft lip with cleft palate	CREBBP, ADCY9	suscepbility loci	GWAS catalog
Nonsyndromic cleft lip with cleft palate	MSX1	suscepbility loci	GWAS catalog
Nonsyndromic cleft lip with cleft palate	FGFR1	suscepbility loci	GWAS catalog
Nonsyndromic cleft lip with cleft palate	PAX7	suscepbility loci	GWAS catalog
Nonsyndromic cleft lip with cleft palate	GADD45G	suscepbility loci	GWAS catalog
Nonsyndromic cleft lip with cleft palate	KRT18	suscepbility loci	GWAS catalog
Nonsyndromic cleft lip with cleft palate	RPS26	suscepbility loci	GWAS catalog
Nonsyndromic cleft lip with cleft palate	SPRY2	suscepbility loci	GWAS catalog
Nonsyndromic cleft lip with cleft palate	GSC	suscepbility loci	GWAS catalog
Nonsyndromic cleft lip with cleft palate	GOSR2	suscepbility loci	GWAS catalog
Nonsyndromic cleft lip with cleft palate	NOG, C17orf67	suscepbility loci	GWAS catalog
Nonsyndromic cleft lip with cleft palate	MAFB	suscepbility loci	GWAS catalog
Nonsyndromic cleft lip with cleft palate	FGF10	suscepbility loci	GWAS catalog
Nonsyndromic cleft lip with cleft palate	MYC, LOC728724	suscepbility loci	GWAS catalog
Cleft lip with or without cleft palate	IRF6	suscepbility loci	GWAS catalog
Cleft lip with or without cleft palate	NTN1	suscepbility loci	GWAS catalog
Cleft lip with or without cleft palate	ARHGAP29	suscepbility loci	GWAS catalog
Cleft lip with or without cleft palate	PAX7	suscepbility loci	GWAS catalog
Cleft lip with or without cleft palate	MAFB	suscepbility loci	GWAS catalog
Cleft lip with or without cleft palate	DCAF4L2	suscepbility loci	GWAS catalog
Cleft lip with or without cleft palate	VAX1	suscepbility loci	GWAS catalog
Cleft lip with or without cleft palate	SPRY2	suscepbility loci	GWAS catalog
Cleft lip with or without cleft palate	NOG	suscepbility loci	GWAS catalog
Cleft lip with or without cleft palate	FAM49A	suscepbility loci	GWAS catalog
Cleft lip with or without cleft palate	TP63	suscepbility loci	GWAS catalog
Cleft lip with or without cleft palate	ARID3B	suscepbility loci	GWAS catalog
Cleft lip with or without cleft palate	COL8A1	suscepbility loci	GWAS catalog
Cleft lip with or without cleft palate	SHROOM3	suscepbility loci	GWAS catalog
Cleft lip with or without cleft palate	KRT18	suscepbility loci	GWAS catalog
Cleft lip with or without cleft palate	PIK3R1	suscepbility loci	GWAS catalog
Cleft lip with or without cleft palate	NRG1	suscepbility loci	GWAS catalog
Cleft lip with or without cleft palate	GOSR2, WNT9B	suscepbility loci	GWAS catalog
Cleft lip with or without cleft palate	PAX7	suscepbility loci	GWAS catalog

Cleft lip with or without cleft palate	DCAF4L2	suscepbility loci	GWAS catalog
Cleft lip with or without cleft palate	intergenic	suscepbility loci	GWAS catalog
Cleft lip with or without cleft palate	NTN1	suscepbility loci	GWAS catalog
Cleft lip with or without cleft palate	TANC2	suscepbility loci	GWAS catalog
Cleft lip with or without cleft palate	ARHGAP29	suscepbility loci	GWAS catalog
Cleft lip with or without cleft palate	IRF6	suscepbility loci	GWAS catalog
Cleft lip with or without cleft palate	VAX1	suscepbility loci	GWAS catalog
Cleft lip with or without cleft palate	KRT18	suscepbility loci	GWAS catalog
Cleft lip with or without cleft palate	NTN1	suscepbility loci	GWAS catalog
Cleft lip with or without cleft palate	MAFB	suscepbility loci	GWAS catalog
Nonsyndromic cleft lip with or without cleft palate	C2orf91, PKDCC	suscepbility loci	GWAS catalog
Nonsyndromic cleft lip with or without cleft palate	C2orf91, PKDCC	suscepbility loci	GWAS catalog
Nonsyndromic cleft lip with or without cleft palate	TMX1	suscepbility loci	GWAS catalog
Nonsyndromic cleft lip with or without cleft palate	MKNK2	suscepbility loci	GWAS catalog
Cleft palate	UGT3A2	suscepbility loci	GWAS catalog
Cleft palate	GRHL3	suscepbility loci	GWAS catalog
Cleft palate	GRHL3	suscepbility loci	GWAS catalog
Cleft palate	LCMT1	suscepbility loci	GWAS catalog
Nonsyndromic cleft lip with or without cleft palate	IRF6	suscepbility loci	GWAS catalog
Nonsyndromic cleft lip with or without cleft palate	KIAA1598	suscepbility loci	GWAS catalog
Nonsyndromic cleft lip with or without cleft palate	CREBBP - LOC102724927	suscepbility loci	GWAS catalog
Nonsyndromic cleft lip with or without cleft palate	NTN1	suscepbility loci	GWAS catalog
Nonsyndromic cleft lip with or without cleft palate	LOC102724968	suscepbility loci	GWAS catalog
Nonsyndromic cleft lip with or without cleft palate	LOC105373888 - LOC105373889	suscepbility loci	GWAS catalog
Nonsyndromic cleft lip with cleft palate	GNB1L	suscepbility loci	GWAS catalog
Nonsyndromic cleft lip with cleft palate	EFHD1 - GIGYF2	suscepbility loci	GWAS catalog
Nonsyndromic cleft lip with cleft palate	FAM49A	suscepbility loci	GWAS catalog
Nonsyndromic cleft lip with cleft palate	DIEXF - SYT14	suscepbility loci	GWAS catalog
Nonsyndromic cleft lip with cleft palate	LOC105369980 - LOC105369981	suscepbility loci	GWAS catalog
Nonsyndromic cleft lip with cleft palate	NAA25	suscepbility loci	GWAS catalog
Nonsyndromic cleft lip with cleft palate	ABCA4	suscepbility loci	GWAS catalog
Nonsyndromic cleft lip with cleft palate	COL8A1	suscepbility loci	GWAS catalog
Nonsyndromic cleft lip with cleft palate	SOX5P1 - LOC100419762	suscepbility loci	GWAS catalog

Nonsyndromic cleft lip with cleft palate	VAX1	susceppbility loci	GWAS catalog
Nonsyndromic cleft lip with cleft palate	TMEM19	susceppbility loci	GWAS catalog
Nonsyndromic cleft lip with cleft palate	FILIP1L, CMSS1	susceppbility loci	GWAS catalog
Nonsyndromic cleft lip with cleft palate	KIAA1429	susceppbility loci	GWAS catalog
Nonsyndromic cleft lip with cleft palate	PTCH1	susceppbility loci	GWAS catalog
Nonsyndromic cleft lip with cleft palate	LOC101928235 - NTN1	susceppbility loci	GWAS catalog
Nonsyndromic cleft lip with cleft palate	LOC105373421 - TAF1B	susceppbility loci	GWAS catalog
Nonsyndromic cleft lip with cleft palate	LOC107986562 - LOC107986563	susceppbility loci	GWAS catalog
Nonsyndromic cleft lip with cleft palate	RAD54B	susceppbility loci	GWAS catalog
Nonsyndromic cleft lip with cleft palate	PTPN11	susceppbility loci	GWAS catalog
Nonsyndromic cleft lip with cleft palate	CLK3, LOC102723750	susceppbility loci	GWAS catalog
Nonsyndromic cleft lip with cleft palate	CREBBP - LOC102724927	susceppbility loci	GWAS catalog
Nonsyndromic cleft lip with cleft palate	LOC101928279 - LINC01396	susceppbility loci	GWAS catalog
Nonsyndromic cleft lip with cleft palate	SALL4	susceppbility loci	GWAS catalog
Nonsyndromic cleft lip with cleft palate	FGFR1	susceppbility loci	GWAS catalog
Nonsyndromic cleft lip with cleft palate	TMCO4	susceppbility loci	GWAS catalog
Nonsyndromic cleft lip with cleft palate	DLG1	susceppbility loci	GWAS catalog
Nonsyndromic cleft lip with cleft palate	RNU5 × 10-4P - KIAA2013	susceppbility loci	GWAS catalog
Nonsyndromic cleft lip with cleft palate	DLC1	susceppbility loci	GWAS catalog
Nonsyndromic cleft lip with cleft palate	PAX7	susceppbility loci	GWAS catalog
Nonsyndromic cleft lip with cleft palate	KCNA2	susceppbility loci	GWAS catalog
Nonsyndromic cleft lip with cleft palate	LOC107985869	susceppbility loci	GWAS catalog
Nonsyndromic cleft lip with cleft palate	LINC01091 - LOC105377407	susceppbility loci	GWAS catalog
Nonsyndromic cleft lip with cleft palate	STK3	susceppbility loci	GWAS catalog
Nonsyndromic cleft lip with cleft palate	VPS13B	susceppbility loci	GWAS catalog
Nonsyndromic cleft lip with cleft palate	LOC105376137 - LOC105376139	susceppbility loci	GWAS catalog
Nonsyndromic cleft lip with cleft palate	PAPPA	susceppbility loci	GWAS catalog
Nonsyndromic cleft lip with cleft palate	CRTAC1	susceppbility loci	GWAS catalog
Nonsyndromic cleft lip with cleft palate	KRT18 - EIF4B	susceppbility loci	GWAS catalog
Nonsyndromic cleft lip with cleft palate	LOC105369780	susceppbility loci	GWAS catalog
Nonsyndromic cleft lip with cleft palate	CUX2	susceppbility loci	GWAS catalog
Nonsyndromic cleft lip with cleft palate	LOC101927216	susceppbility loci	GWAS catalog
Nonsyndromic cleft lip with cleft palate	SLC25A21	susceppbility loci	GWAS catalog

Nonsyndromic cleft lip with cleft palate	LINC00640 - LOC105370496	suscepbility loci	GWAS catalog
Nonsyndromic cleft lip with cleft palate	LOC107984693 - LOC107984639	suscepbility loci	GWAS catalog
Nonsyndromic cleft lip with cleft palate	UBL7	suscepbility loci	GWAS catalog
Nonsyndromic cleft lip with cleft palate	GOSR2	suscepbility loci	GWAS catalog
Nonsyndromic cleft lip with cleft palate	NOG - C17orf67	suscepbility loci	GWAS catalog
Nonsyndromic cleft lip with cleft palate	LOC102724968 - LOC105372620	suscepbility loci	GWAS catalog
Nonsyndromic cleft lip with cleft palate	MTMR3	suscepbility loci	GWAS catalog
Nonsyndromic cleft lip with cleft palate	SFI1	suscepbility loci	GWAS catalog
Nonsyndromic cleft lip with cleft palate	DEPDC5	suscepbility loci	GWAS catalog
Nonsyndromic cleft lip with cleft palate	MYH9	suscepbility loci	GWAS catalog
Nonsyndromic cleft lip with cleft palate	LOC105373887 - LOC105373888	suscepbility loci	GWAS catalog
Nonsyndromic cleft lip with cleft palate	LINC01091	suscepbility loci	GWAS catalog
Nonsyndromic cleft lip with cleft palate	RNU6-381P - FGF10	suscepbility loci	GWAS catalog
Nonsyndromic cleft lip with cleft palate	LINC00824 - LINC00977	suscepbility loci	GWAS catalog
Nonsyndromic cleft lip with or without cleft palate	C2orf91	suscepbility loci	GWAS catalog
Nonsyndromic cleft lip with or without cleft palate	C2orf91	suscepbility loci	GWAS catalog
Nonsyndromic cleft lip with or without cleft palate	LOC105373887	suscepbility loci	GWAS catalog
Nonsyndromic cleft lip with or without cleft palate	LOC101927697 - EBF1	suscepbility loci	GWAS catalog
Nonsyndromic cleft lip with or without cleft palate	LOC442161 - TFAP2A	suscepbility loci	GWAS catalog
Nonsyndromic cleft lip with or without cleft palate	POU5F1 - PSORS1C3	suscepbility loci	GWAS catalog
Nonsyndromic cleft lip with or without cleft palate	POU5F1 - PSORS1C3	suscepbility loci	GWAS catalog
Nonsyndromic cleft lip with or without cleft palate	LOC107986952	suscepbility loci	GWAS catalog
Nonsyndromic cleft lip with or without cleft palate	SEMA4D	suscepbility loci	GWAS catalog
Nonsyndromic cleft lip with or without cleft palate	LOC105376137 - LOC105376139	suscepbility loci	GWAS catalog
Nonsyndromic cleft lip with or without cleft palate	LINC00640 - LOC105370496	suscepbility loci	GWAS catalog
Nonsyndromic cleft lip with or without cleft palate	LINC00640 - LOC105370496	suscepbility loci	GWAS catalog
Nonsyndromic cleft lip with or without cleft palate	MKNK2	suscepbility loci	GWAS catalog
Cleft lip with or without cleft palate	LINC00824 - LINC00977	suscepbility loci	GWAS catalog
Cleft lip with or without cleft palate	IRF6 - DIEXF	suscepbility loci	GWAS catalog
Cleft lip with or without cleft palate	NTN1	suscepbility loci	GWAS catalog
Cleft lip with or without cleft palate	ABCA4	suscepbility loci	GWAS catalog
Cleft lip with or without cleft palate	PAX7	suscepbility loci	GWAS catalog
Cleft lip with or without cleft palate	LOC102724968	suscepbility loci	GWAS catalog

Cleft lip with or without cleft palate	SOX5P1 - LOC100419762	suscepbility loci	GWAS catalog
Cleft lip with or without cleft palate	KIAA1598	suscepbility loci	GWAS catalog
Cleft lip with or without cleft palate	LOC101927216 - LOC105370275	suscepbility loci	GWAS catalog
Cleft lip with or without cleft palate	NOG - C17orf67	suscepbility loci	GWAS catalog
Cleft lip with or without cleft palate	LOC105373444 - FAM49A	suscepbility loci	GWAS catalog
Cleft lip with or without cleft palate	TP63	suscepbility loci	GWAS catalog
Cleft lip with or without cleft palate	ARID3B	suscepbility loci	GWAS catalog
Cleft lip with or without cleft palate	CMSS1, FILIP1L	suscepbility loci	GWAS catalog
Cleft lip with or without cleft palate	SHROOM3, LOC105377290	suscepbility loci	GWAS catalog
Cleft lip with or without cleft palate	KRT18 - EIF4B	suscepbility loci	GWAS catalog
Cleft lip with or without cleft palate	EEF1B2P2 - LOC102467655	suscepbility loci	GWAS catalog
Cleft lip with or without cleft palate	NRG1	suscepbility loci	GWAS catalog
Cleft lip with or without cleft palate	GOSR2	suscepbility loci	GWAS catalog
Cleft lip with or without cleft palate	PAX7	suscepbility loci	GWAS catalog
Cleft lip with or without cleft palate	SOX5P1 - LOC100419762	suscepbility loci	GWAS catalog
Cleft lip with or without cleft palate	LINC00824 - LINC00977	suscepbility loci	GWAS catalog
Cleft lip with or without cleft palate	NTN1	suscepbility loci	GWAS catalog
Cleft lip with or without cleft palate	TANC2	suscepbility loci	GWAS catalog
Cleft lip with or without cleft palate	RNF24	suscepbility loci	GWAS catalog
Cleft lip with or without cleft palate	ABCA4	suscepbility loci	GWAS catalog
Cleft lip with or without cleft palate	IRF6 - DIEXF	suscepbility loci	GWAS catalog
Cleft lip with or without cleft palate	CMSS1, FILIP1L	suscepbility loci	GWAS catalog
Cleft lip with or without cleft palate	KIAA1598	suscepbility loci	GWAS catalog
Cleft lip with or without cleft palate	KRT18 - EIF4B	suscepbility loci	GWAS catalog
Cleft lip with or without cleft palate	NTN1	suscepbility loci	GWAS catalog
Cleft lip with or without cleft palate	LOC102724968 - LOC105372620	suscepbility loci	GWAS catalog
Cleft palate	UGT3A2	suscepbility loci	GWAS catalog
Cleft palate	GRHL3	suscepbility loci	GWAS catalog
Cleft palate	NIPAL3 - RCAN3AS	suscepbility loci	GWAS catalog
Cleft palate	LOC101928477	suscepbility loci	GWAS catalog
Cleft palate	HNRNPA1P4 - LOC105375931	suscepbility loci	GWAS catalog
Cleft palate	LOC105375782 - CHRAC1	suscepbility loci	GWAS catalog
Cleft palate	RN7SL557P - AQP8	suscepbility loci	GWAS catalog

Cleft palate (environmental tobacco smoke interaction)	SLC2A9	suscepbility loci	GWAS catalog
Cleft lip	LINC00824 - LINC00977	suscepbility loci	GWAS catalog
Cleft lip	IRF6 - DIEXF	suscepbility loci	GWAS catalog
cleft lip	ABCA4	mix	Phynolizer
cleft lip	ANKFN1	mix	Phynolizer
cleft lip	ANKS6	mix	Phynolizer
cleft lip	BCL3	mix	Phynolizer
cleft lip	BHMT2	mix	Phynolizer
cleft lip	BMP6	mix	Phynolizer
cleft lip	CDH2	mix	Phynolizer
cleft lip	CRLF1	mix	Phynolizer
cleft lip	DPF3	mix	Phynolizer
cleft lip	ERBB2	mix	Phynolizer
cleft lip	FGF10	mix	Phynolizer
cleft lip	FGF2	mix	Phynolizer
cleft lip	FGF3	mix	Phynolizer
cleft lip	FGF7	mix	Phynolizer
cleft lip	FGF8	mix	Phynolizer
cleft lip	FGF9	mix	Phynolizer
cleft lip	FHIT	mix	Phynolizer
cleft lip	GAD1	mix	Phynolizer
cleft lip	GART	mix	Phynolizer
cleft lip	GREM1	mix	Phynolizer
cleft lip	GRM7	mix	Phynolizer
cleft lip	HIC1	mix	Phynolizer
cleft lip	IRF6	mix	Phynolizer
cleft lip	KIAA1598	mix	Phynolizer
cleft lip	KLHL26	mix	Phynolizer
cleft lip	MAFB	mix	Phynolizer
cleft lip	MMP3	mix	Phynolizer
cleft lip	MSX1	mix	Phynolizer
cleft lip	MTHFR	mix	Phynolizer

cleft lip	MTHFS	mix	Phynolizer
cleft lip	MTR	mix	Phynolizer
cleft lip	NDFIP2	mix	Phynolizer
cleft lip	NOG	mix	Phynolizer
cleft lip	NOS3	mix	Phynolizer
cleft lip	NRXN3	mix	Phynolizer
cleft palate	ATXN3	mix	Phynolizer
cleft palate	BNC2	mix	Phynolizer
cleft palate	CASK	mix	Phynolizer
cleft palate	CBFB	mix	Phynolizer
cleft palate	CHUK	mix	Phynolizer
cleft palate	COL2A1	mix	Phynolizer
cleft palate	DLG1	mix	Phynolizer
cleft palate	EGF	mix	Phynolizer
cleft palate	FZD4	mix	Phynolizer
cleft palate	MNT	mix	Phynolizer
cleft palate	MRM1	mix	Phynolizer
cleft palate	COL2A1	mix	Phynolizer
cleft palate	FGF8	mix	Phynolizer
cleft palate	IRF6	mix	Phynolizer
cleft palate	MSX1	mix	Phynolizer
cleft palate	SUMO1	mix	Phynolizer
cleft palate	TBX22	mix	Phynolizer
cleft palate	CASK	mix	Phynolizer
cleft palate	MTR	mix	Phynolizer
cleft palate	FGF10	mix	Phynolizer
cleft palate	TGFB3	mix	Phynolizer
cleft palate	RYK	mix	Phynolizer
cleft palate	PDGFRA	mix	Phynolizer
cleft palate	CBFB	mix	Phynolizer
cleft palate	TYMS	mix	Phynolizer
cleft palate	NOS3	mix	Phynolizer
cleft palate	DLG1	mix	Phynolizer

cleft palate	RARG	mix	Phynolizer
cleft palate	MNT	mix	Phynolizer
cleft palate	BHMT2	mix	Phynolizer
cleft palate	EGF	mix	Phynolizer
cleft palate	FGF3	mix	Phynolizer
cleft palate	SIM2	mix	Phynolizer
cleft palate	CHUK	mix	Phynolizer
cleft palate	FGF7	mix	Phynolizer
cleft palate	FGF9	mix	Phynolizer
cleft palate	FGF2	mix	Phynolizer
cleft palate	SLC19A1	mix	Phynolizer
cleft palate	FZD4	mix	Phynolizer
cleft palate	BNC2	mix	Phynolizer
cleft palate	MTHFS	mix	Phynolizer
cleft palate	SPRY2	mix	Phynolizer
cleft palate	SH3BP4	mix	Phynolizer
cleft palate	MRM1	mix	Phynolizer
cleft palate	ATXN3	mix	Phynolizer
cleft palate	RASGRF2	mix	Phynolizer
cleft palate	OR2AH1P	mix	Phynolizer
cleft palate	TXNDC16	mix	Phynolizer
cleft palate isolated	UBB	mix	Phynolizer
cleft lip	ABCA4	mix	Phynolizer
cleft lip	ANKFN1	mix	Phynolizer
cleft lip	ANKS6	mix	Phynolizer
cleft lip	BCL3	mix	Phynolizer
cleft lip	BHMT2	mix	Phynolizer
cleft lip	BMP6	mix	Phynolizer
cleft lip	CDH2	mix	Phynolizer
cleft lip	CRLF1	mix	Phynolizer
cleft lip	DPF3	mix	Phynolizer
cleft lip	ERBB2	mix	Phynolizer
cleft lip	FGF10	mix	Phynolizer

cleft lip	FGF2	mix	Phynolizer
cleft lip	FGF3	mix	Phynolizer
cleft lip	FGF7	mix	Phynolizer
cleft lip	FGF8	mix	Phynolizer
cleft lip	FGF9	mix	Phynolizer
cleft lip	FHIT	mix	Phynolizer
cleft lip	GAD1	mix	Phynolizer
cleft lip	GART	mix	Phynolizer
cleft lip	GREM1	mix	Phynolizer
cleft lip	GRM7	mix	Phynolizer
cleft lip	HIC1	mix	Phynolizer
cleft lip	IRF6	mix	Phynolizer
cleft lip	KIAA1598	mix	Phynolizer
cleft lip	KLHL26	mix	Phynolizer
cleft lip	MAFB	mix	Phynolizer
cleft lip	MMP3	mix	Phynolizer
cleft lip	MSX1	mix	Phynolizer
cleft lip	MTHFR	mix	Phynolizer
cleft lip	MTHFS	mix	Phynolizer
cleft lip	MTR	mix	Phynolizer
cleft lip	NDFIP2	mix	Phynolizer
cleft lip	NOG	mix	Phynolizer
cleft lip	NOS3	mix	Phynolizer
cleft lip	NRXN3	mix	Phynolizer
cleft lip	PDGFC	mix	Phynolizer
cleft lip	PVT1	mix	Phynolizer
cleft lip	RARA	mix	Phynolizer
cleft lip	RPS2P6	mix	Phynolizer
cleft lip	SLC19A1	mix	Phynolizer
cleft lip	SPRY2	mix	Phynolizer
cleft lip	SUMO1	mix	Phynolizer
cleft lip	TCN2	mix	Phynolizer
cleft lip	TGFA	mix	Phynolizer

cleft lip	TGFB3	mix	Phynolizer
cleft lip	THADA	mix	Phynolizer
cleft lip	THAS	mix	Phynolizer
cleft lip	TYMS	mix	Phynolizer
cleft palate	ATXN3	mix	Phynolizer
cleft palate	BNC2	mix	Phynolizer
cleft palate	CASK	mix	Phynolizer
cleft palate	CBFB	mix	Phynolizer
cleft palate	CHUK	mix	Phynolizer
cleft palate	COL2A1	mix	Phynolizer
cleft palate	DLG1	mix	Phynolizer
cleft palate	EGF	mix	Phynolizer
cleft palate	FZD4	mix	Phynolizer
cleft palate	MNT	mix	Phynolizer
cleft palate	MRM1	mix	Phynolizer
cleft palate	OR2AH1P	mix	Phynolizer
cleft palate	PDGFRA	mix	Phynolizer
cleft palate	RARG	mix	Phynolizer
cleft palate	RASGRF2	mix	Phynolizer
cleft palate	RYK	mix	Phynolizer
cleft palate	SH3BP4	mix	Phynolizer
cleft palate	SIM2	mix	Phynolizer
cleft palate	TBX22	mix	Phynolizer
cleft palate	TXNDC16	mix	Phynolizer
cleft palate isolated	UBB	mix	Phynolizer
cleft palate	UBB	phenotype	HPO
cleft palate	TBX22	phenotype	HPO
