# Supplementary material for: Genetic factors define CPO and CLO subtypes of nonsyndromicorofacial cleft
Source: PLoS Genet. 2019 Oct 14;15(10):e1008357. doi: 10.1371/journal.pgen.1008357 (PMC6812857; doi:10.1371/journal.pgen.1008357)
Supplement: S6 Table — (PDF) [file pgen.1008357.s014.pdf]

Supplementary Table 6. Discovery and replication results of the rest SNPs selected for validation by gene network and ontology analysis

SNP	Gene(a)	Position(hg19)	Alleles	Disease	Discovery(c)			Replication CHS(d)			Replication CHB(e)			Combined(f)		
					MAF(b)	P	OR	MAF(b)	P	OR	MAF(b)	P	OR	P	OR	I
rs10040353	*SOX30*	5:157067491	A/G	CPO	0.322/0.34	1.55 × 10-1	0.9222	0.33/0.325	7.59 × 10-1	1.020	0.361/0.35	6.40 × 10-1	1.048	5.47 × 10-1	0.977	0
				CLO	0.296/0.34	4.87 × 10-4	0.818	0.304/0.325	1.26 × 10-1	0.906	0.373/0.35	2.98 × 10-1	1.105	4.02 × 10-3	0.893	72.61
				CLP				0.322/0.325	7.68 × 10-1	0.983	0.334/0.35	5.13 × 10-1	0.932	5.66 × 10-1	0.971	0
rs10514487	*LINC01229;LOC102724084*	16:80187374	T/C	CPO	0.137/0.073	2.93 × 10-6	2.012	0.147/0.135	2.39 × 10-1	1.107	0.128/0.106	1.58 × 10-1	1.233	9.53 × 10-5	1.462	92.36
				CLO	0.13/0.073	3.51 × 10-6	1.886	0.125/0.135	3.58 × 10-1	0.921	0.118/0.105	3.74 × 10-1	1.139	2.00 × 10-6	1.309	94.29
				CLP				0.159/0.135	1.25 × 10-2	1.214	0.127/0.106	1.97 × 10-1	1.226	5.04 × 10-3	1.216	0
rs10761382	*FANCC;PTCH1*	9:98105316	C/G	CPO	0.501/0.455	6.64 × 10-4	1.199	0.462/0.464	8.81 × 10-1	0.991	0.463/0.432	1.84 × 10-1	1.136	5.75 × 10-3	1.107	64.85
				CLO	0.454/0.455	9.51 × 10-1	0.997	0.416/0.464	1.33 × 10-3	0.824	0.454/0.432	3.18 × 10-1	1.097	1.10 × 10-1	0.943	77.14
				CLP				0.457/0.464	6.28 × 10-1	0.974	0.425/0.432	8.06 × 10-1	0.975	5.87 × 10-1	0.974	0
rs10941658	*NNT;FGF10*	5:44173808	A/G	CPO	0.437/0.411	5.31 × 10-2	1.11	0.442/0.424	2.29 × 10-1	1.075	0.385/0.413	2.27 × 10-1	0.889	1.08 × 10-1	1.062	50.57
				CLO	0.458/0.411	3.78 × 10-4	1.209	0.44/0.424	2.88 × 10-1	1.066	0.432/0.414	4.13 × 10-1	1.079	6.36 × 10-4	1.134	28
				CLP				0.475/0.424	2.40 × 10-4	1.225	0.417/0.413	8.76 × 10-1	1.016	9.32 × 10-4	1.175	60.9
rs11237212	*FGF3;LOC101928443*	11:69875274	C/T	CPO	0.218/0.261	1.99 × 10-4	0.7889	0.239/0.258	1.47 × 10-1	0.904	0.263/0.257	7.88 × 10-1	1.030	9.39 × 10-4	0.867	60.24
				CLO	0.243/0.261	1.14 × 10-1	0.907	0.238/0.258	1.23 × 10-1	0.898	0.286/0.258	1.63 × 10-1	1.155	1.48 × 10-1	0.941	57.53
				CLP				0.24/0.258	1.20 × 10-1	0.905	0.231/0.257	2.30 × 10-1	0.867	5.24 × 10-2	0.897	0
rs11739623	*IRF1;IL5*	5:131864152	T/C	CPO	0.356/0.402	4.29 × 10-4	0.8227	0.403/0.39	3.76 × 10-1	1.056	0.471/0.447	3.17 × 10-1	1.100	1.46 × 10-1	0.947	83.4
				CLO	0.378/0.402	7.30 × 10-2	0.907	0.375/0.39	2.97 × 10-1	0.938	0.46/0.446	5.39 × 10-1	1.058	1.07 × 10-1	0.942	3.37
				CLP				0.376/0.39	2.69 × 10-1	0.940	0.452/0.447	8.69 × 10-1	1.017	3.75 × 10-1	0.957	0
rs117555620	*GSC*	14:95235358	A/G	CPO	0.047/0.06	2.67 × 10-2	0.7611	0.05/0.054	5.33 × 10-1	0.919	0.056/0.055	8.81 × 10-1	1.032	6.90 × 10-2	0.859	0
				CLO	0.039/0.06	5.06 × 10-4	0.636	0.063/0.054	1.92 × 10-1	1.180	0.059/0.055	7.27 × 10-1	1.072	2.36 × 10-1	0.906	83.77
				CLP				0.051/0.054	6.59 × 10-1	0.947	0.05/0.055	6.53 × 10-1	0.902	5.47 × 10-1	0.937	0
rs12051608	*KCNJ16*	17:68126634	C/G	CPO	0.173/0.209	6.83 × 10-4	0.7901	0.255/0.242	3.28 × 10-1	1.070	0.241/0.23	5.88 × 10-1	1.063	1.79 × 10-1	0.941	81.72
				CLO	0.167/0.209	7.85 × 10-5	0.760	0.256/0.242	3.10 × 10-1	1.073	0.231/0.23	9.92 × 10-1	1.001	6.24 × 10-2	0.920	84.72
				CLP				0.26/0.242	1.48 × 10-1	1.096	0.275/0.23	4.06 × 10-2	1.270	2.47 × 10-2	1.133	18.58
rs12193833	*GMDS*	6:1670604	G/T	CPO	0.517/0.437	1.93 × 10-6	1.378	0.504/0.495	5.60 × 10-1	1.036	0.485/0.479	7.92 × 10-1	1.025	4.56 × 10-5	1.184	86.91
				CLO	0.483/0.437	4.50 × 10-4	1.205	0.497/0.495	9.40 × 10-1	1.005	0.504/0.478	2.51 × 10-1	1.111	3.60 × 10-3	1.112	61.23
				CLP				0.48/0.495	2.50 × 10-1	0.939	0.48/0.479	9.75 × 10-1	1.003	3.18 × 10-1	0.953	0
rs12264204	*WDR11;FGFR2*	10:122809823	C/T	CPO	0.095/0.068	8.40 × 10-5	1.449	0.08/0.08	9.41 × 10-1	0.992	0.059/0.054	6.44 × 10-1	1.100	3.56 × 10-3	1.219	71.58
				CLO	0.091/0.068	6.59 × 10-4	1.382	0.094/0.08	8.93 × 10-2	1.197	0.071/0.054	1.33 × 10-2	1.335	7.82 × 10-5	1.300	0

				CLP				0.082/0.08	7.62 × 10-1	1.031	0.064/0.054	3.72 × 10-1	1.212	5.14 × 10-1	1.061	0
rs12431978	*BMP4;CDKN3*	14:54801587	C/A	CPO	0.458/0.415	8.90 × 10-4	1.195	0.432/0.434	8.84 × 10-1	0.991	0.399/0.394	8.34 × 10-1	1.021	2.24 × 10-2	1.089	65.77
				CLO	0.453/0.415	3.08 × 10-3	1.171	0.434/0.434	9.73 × 10-1	0.998	0.363/0.392	1.85 × 10-1	0.882	1.33 × 10-1	1.057	75.74
				CLP				0.453/0.434	1.76 × 10-1	1.078	0.425/0.394	2.04 × 10-1	1.140	7.23 × 10-2	1.092	0
rs12879197	*FOXG1-AS1*	14:29206592	A/T	CPO	0.123/0.093	1.96 × 10-4	1.365	0.114/0.122	4.07 × 10-1	0.926	0.069/0.066	7.94 × 10-1	1.051	2.94 × 10-2	1.138	79.55
				CLO	0.109/0.093	4.56 × 10-2	1.189	0.116/0.122	5.47 × 10-1	0.946	0.081/0.065	1.96 × 10-1	1.259	1.56 × 10-1	1.088	50.22
				CLP				0.115/0.122	4.85 × 10-1	0.942	0.075/0.066	5.08 × 10-1	1.140	7.02 × 10-1	0.971	0
rs12944649	*SOX15*	17:7492228	G/A	CPO	0.179/0.192	2.12 × 10-1	0.9172	0.172/0.171	9.24 × 10-1	1.008	0.221/0.24	3.52 × 10-1	0.900	2.36 × 10-1	0.945	0
				CLO	0.158/0.192	8.61 × 10-4	0.788	0.177/0.171	6.13 × 10-1	1.041	0.214/0.24	1.65 × 10-1	0.859	1.20 × 10-2	0.887	71.18
				CLP				0.172/0.171	9.53 × 10-1	1.004	0.234/0.24	7.82 × 10-1	0.967	9.23 × 10-1	0.994	0
rs13021092	*DLX2-AS1;ITGA6*	2:173279296	G/A	CPO	0.132/0.145	1.69 × 10-1	0.8979	0.124/0.127	7.07 × 10-1	0.967	0.144/0.188	1.33 × 10-2	0.727	2.78 × 10-2	0.888	39.33
				CLO	0.111/0.145	1.76 × 10-4	0.735	0.137/0.127	3.33 × 10-1	1.089	0.189/0.187	9.27 × 10-1	1.011	7.21 × 10-2	0.908	82.92
				CLP				0.117/0.127	2.36 × 10-1	0.906	0.172/0.188	4.18 × 10-1	0.898	1.52 × 10-1	0.904	0
rs13222549	*EGFR*	7:55225140	C/G	CPO	0.101/0.084	3.04 × 10-2	1.216	0.109/0.116	4.87 × 10-1	0.936	0.075/0.088	3.35 × 10-1	0.845	4.96 × 10-1	1.043	64.39
				CLO	0.116/0.084	3.60 × 10-5	1.424	0.108/0.116	4.19 × 10-1	0.926	0.081/0.088	5.66 × 10-1	0.909	3.27 × 10-2	1.136	85
				CLP				0.115/0.116	9.20 × 10-1	0.991	0.089/0.088	9.15 × 10-1	1.019	9.64 × 10-1	0.997	0
rs13267937	*MAFA-AS1;MAFA*	8:144506133	G/C	CPO	0.146/0.055	2.69 × 10-6	2.963	0.333/0.3	1.71 × 10-2	1.166	0.266/0.272	7.88 × 10-1	0.972	1.22 × 10-6	1.472	97.84
				CLO	0.167/0.055	2.62 × 10-6	3.469	0.294/0.3	6.95 × 10-1	0.975	0.279/0.272	7.37 × 10-1	1.035	7.27 × 10-2	1.100	95.31
				CLP				0.305/0.3	6.63 × 10-1	1.026	0.273/0.272	9.68 × 10-1	1.005	6.89 × 10-1	1.021	0
rs163503	*SIX3;SIX2*	2:45197139	A/G	CPO	0.093/0.065	4.28 × 10-5	1.478	0.127/0.123	6.27 × 10-1	1.046	0.105/0.145	1.25 × 10-2	0.694	6.26 × 10-2	1.119	89.75
				CLO	0.069/0.065	5.62 × 10-1	1.063	0.109/0.123	1.70 × 10-1	0.877	0.113/0.145	3.57 × 10-2	0.747	1.31 × 10-1	0.909	53.72
				CLP				0.129/0.123	4.80 × 10-1	1.061	0.087/0.145	4.69 × 10-4	0.562	3.50 × 10-1	0.932	91.39
rs17050267	*LINC01101;GLI2*	2:121303783	C/T	CPO	0.165/0.173	4.28 × 10-1	0.9448	0.156/0.147	4.46 × 10-1	1.066	0.168/0.16	6.40 × 10-1	1.062	9.32 × 10-1	1.004	0
				CLO	0.137/0.173	2.13 × 10-4	0.757	0.169/0.147	4.33 × 10-2	1.179	0.162/0.16	9.19 × 10-1	1.013	2.36 × 10-1	0.942	87.74
				CLP				0.144/0.147	7.42 × 10-1	0.975	0.19/0.16	1.17 × 10-1	1.232	6.15 × 10-1	1.034	56.46
rs1899449	*IL20RB;SOX14*	3:137250523	A/G	CPO	0.183/0.134	2.86 × 10-6	1.44	0.146/0.143	8.17 × 10-1	1.020	0.126/0.124	9.28 × 10-1	1.013	1.25 × 10-4	1.217	82.57
				CLO	0.159/0.134	6.68 × 10-3	1.221	0.153/0.143	3.69 × 10-1	1.078	0.15/0.124	1.06 × 10-1	1.243	2.33 × 10-3	1.169	0
				CLP				0.144/0.143	9.59 × 10-1	1.004	0.122/0.124	8.83 × 10-1	0.977	9.83 × 10-1	0.999	0
rs35506173	*FOXA3*	19:46374916	T/C	CPO	0.294/0.265	1.52 × 10-2	1.154	0.297/0.274	8.71 × 10-2	1.120	0.232/0.23	9.31 × 10-1	1.010	5.47 × 10-3	1.121	0
				CLO	0.309/0.265	2.34 × 10-4	1.238	0.294/0.274	1.32 × 10-1	1.105	0.241/0.228	5.23 × 10-1	1.072	1.92 × 10-4	1.163	13.76
				CLP				0.289/0.274	2.33 × 10-1	1.076	0.202/0.23	1.81 × 10-1	0.847	6.29 × 10-1	1.027	66.58
rs4674606	*EPHA4;PAX3*	2:222630718	A/G	CPO	0.26/0.302	5.56 × 10-4	0.8123	0.276/0.241	8.41 × 10-3	1.197	0.321/0.327	8.11 × 10-1	0.976	3.82 × 10-1	0.965	88.96
				CLO	0.256/0.302	1.39 × 10-4	0.796	0.274/0.241	1.29 × 10-2	1.185	0.32/0.326	7.75 × 10-1	0.972	2.22 × 10-1	0.951	89.61

				CLP				0.261/0.241	8.92 × 10-2	1.114	0.288/0.327	1.00 × 10-1	0.834	5.07 × 10-1	1.037	80.65
rs4743148	*PTCH1;LINC00476*	9:98430574	A/G	CPO	0.266/0.232	3.05 × 10-3	1.198	0.282/0.297	2.76 × 10-1	0.930	0.232/0.226	7.79 × 10-1	1.032	1.51 × 10-1	1.062	74.82
				CLO	0.283/0.232	6.95 × 10-6	1.308	0.304/0.297	6.15 × 10-1	1.033	0.203/0.227	2.06 × 10-1	0.868	3.37 × 10-3	1.128	85.1
				CLP				0.288/0.297	4.59 × 10-1	0.956	0.223/0.226	8.85 × 10-1	0.983	4.67 × 10-1	0.961	0
rs4750967	*LINC01163;MGMT*	10:130301922	G/C	CPO	0.469/0.43	2.97 × 10-3	1.173	0.464/0.443	1.56 × 10-1	1.089	0.433/0.434	9.62 × 10-1	0.995	3.69 × 10-3	1.113	17.9
				CLO	0.483/0.43	5.02 × 10-5	1.240	0.446/0.443	8.32 × 10-1	1.013	0.447/0.434	5.56 × 10-1	1.056	1.64 × 10-3	1.122	70.85
				CLP				0.446/0.443	8.49 × 10-1	1.011	0.439/0.434	8.55 × 10-1	1.019	7.94 × 10-1	1.013	0
rs4855009	*DNAJC19;SOX2-OT*	3:180739934	T/A	CPO	0.246/0.232	2.15 × 10-1	1.08	0.264/0.265	9.76 × 10-1	0.998	0.192/0.198	7.57 × 10-1	0.963	4.71 × 10-1	1.031	0
				CLO	0.27/0.232	7.31 × 10-4	1.226	0.264/0.265	9.34 × 10-1	0.994	0.181/0.198	3.31 × 10-1	0.892	5.17 × 10-2	1.085	76.54
				CLP				0.276/0.265	3.73 × 10-1	1.057	0.196/0.198	9.18 × 10-1	0.987	4.46 × 10-1	1.043	0
rs4951107	*ATP2B4*	1:203687154	T/G	CPO	0.376/0.36	1.93 × 10-1	1.075	0.405/0.383	1.22 × 10-1	1.100	0.328/0.355	2.46 × 10-1	0.890	1.54 × 10-1	1.056	41.94
				CLO	0.403/0.36	7.26 × 10-4	1.201	0.362/0.383	1.49 × 10-1	0.915	0.358/0.355	8.95 × 10-1	1.013	1.32 × 10-1	1.058	82.14
				CLP				0.37/0.383	3.50 × 10-1	0.949	0.31/0.355	6.27 × 10-2	0.818	9.11 × 10-2	0.919	32.38
rs58013292	*PAX8*	2:114028483	T/C	CPO	0.13/0.096	2.29 × 10-5	1.414	0.115/0.113	8.27 × 10-1	1.021	0.085/0.082	7.85 × 10-1	1.048	1.27 × 10-3	1.207	73.43
				CLO	0.115/0.096	1.65 × 10-2	1.225	0.11/0.113	7.39 × 10-1	0.969	0.085/0.082	8.24 × 10-1	1.038	1.24 × 10-1	1.095	43.07
				CLP				0.101/0.113	1.78 × 10-1	0.888	0.083/0.082	9.04 × 10-1	1.022	2.46 × 10-1	0.911	0
rs60693783	*SEM1;DLX6-AS1*	7:96488067	A/G	CPO	0.092/0.077	3.53 × 10-2	1.219	0.081/0.092	1.86 × 10-1	0.868	0.067/0.059	5.07 × 10-1	1.139	3.74 × 10-1	1.061	65.54
				CLO	0.104/0.077	1.77 × 10-4	1.400	0.083/0.092	2.94 × 10-1	0.895	0.072/0.059	2.87 × 10-1	1.220	1.61 × 10-2	1.168	80.81
				CLP				0.101/0.092	2.67 × 10-1	1.109	0.082/0.059	7.95 × 10-2	1.415	8.06 × 10-2	1.159	18.85
rs622755	*RAB2A;CHD7*	8:61580676	A/G	CPO	0.425/0.377	2.51 × 10-4	1.22	0.39/0.398	5.73 × 10-1	0.966	0.369/0.387	4.27 × 10-1	0.925	5.90 × 10-2	1.074	81.5
				CLO	0.382/0.377	7.22 × 10-1	1.020	0.364/0.398	2.04 × 10-2	0.867	0.394/0.387	7.60 × 10-1	1.029	2.97 × 10-1	0.962	55.69
				CLP				0.378/0.398	1.40 × 10-1	0.920	0.392/0.387	8.47 × 10-1	1.020	2.27 × 10-1	0.942	0
rs6776327	*SOX2-OT*	3:181234224	G/C	CPO	0.328/0.285	3.29 × 10-4	1.229	0.297/0.306	5.06 × 10-1	0.957	0.255/0.258	8.56 × 10-1	0.980	4.18 × 10-2	1.085	78.33
				CLO	0.317/0.285	6.91 × 10-3	1.168	0.328/0.306	1.19 × 10-1	1.105	0.253/0.258	7.89 × 10-1	0.972	6.33 × 10-3	1.114	15.32
				CLP				0.313/0.306	5.73 × 10-1	1.034	0.25/0.258	7.05 × 10-1	0.957	7.41 × 10-1	1.018	0
rs7143653	*BMP4;CDKN3*	14:54852206	C/T	CPO	0.256/0.251	6.34 × 10-1	1.03	0.292/0.263	3.64 × 10-2	1.151	0.241/0.241	9.93 × 10-1	1.001	1.01 × 10-1	1.071	0
				CLO	0.292/0.251	3.77 × 10-4	1.233	0.272/0.263	4.97 × 10-1	1.047	0.242/0.242	9.89 × 10-1	1.001	3.83 × 10-3	1.126	57.51
				CLP				0.272/0.263	4.59 × 10-1	1.047	0.247/0.241	7.84 × 10-1	1.033	4.35 × 10-1	1.044	0
rs7153737	*FOXN3*	14:89669940	C/G	CPO	0.369/0.351	1.50 × 10-1	1.083	0.446/0.414	3.15 × 10-2	1.139	0.437/0.387	3.44 × 10-2	1.228	1.71 × 10-3	1.126	0
				CLO	0.393/0.351	8.70 × 10-4	1.199	0.432/0.414	2.27 × 10-1	1.076	0.436/0.388	3.28 × 10-2	1.221	1.14 × 10-4	1.154	8.97
				CLP				0.435/0.414	1.20 × 10-1	1.090	0.371/0.387	5.25 × 10-1	0.936	2.83 × 10-1	1.054	39.7
rs7722035	*FGF1*	5:142055973	C/G	CPO	0.44/0.498	1.30 × 10-5	0.7914	0.504/0.493	4.81 × 10-1	1.043	0.495/0.5	8.46 × 10-1	0.982	1.29 × 10-2	0.912	85
				CLO	0.436/0.498	2.47 × 10-6	0.778	0.507/0.493	3.65 × 10-1	1.056	0.487/0.501	5.41 × 10-1	0.945	3.67 × 10-3	0.899	86.59

				CLP				0.482/0.507	7.00 × 10-2	0.905	0.488/0.5	6.45 × 10-1	0.954	1.69 × 10-1	0.936	37.67
rs7727165	*CPEB4;C5orf47*	5:173413036	A/C	CPO	0.093/0.026	3.75 × 10-6	3.834	0.158/0.142	1.24 × 10-1	1.138	0.127/0.124	8.85 × 10-1	1.021	6.32 × 10-6	1.583	97.64
				CLO	0.098/0.046	5.68 × 10-5	4.071	0.174/0.142	3.45 × 10-3	1.270	0.13/0.125	7.56 × 10-1	1.044	3.89 × 10-6	1.362	93.56
				CLP				0.16/0.142	6.95 × 10-2	1.150	0.12/0.124	8.01 × 10-1	0.962	1.30 × 10-1	1.110	6.06
rs78516834	*IL20RB;SOX14*	3:137466848	G/A	CPO	0.05/0.051	9.63 × 10-1	0.9944	0.063/0.054	2.18 × 10-1	1.171	0.057/0.059	9.06 × 10-1	0.976	4.83 × 10-1	1.059	0
				CLO	0.071/0.051	6.25 × 10-4	1.442	0.064/0.054	1.69 × 10-1	1.192	0.062/0.059	7.55 × 10-1	1.062	8.53 × 10-4	1.287	18.74
				CLP				0.06/0.054	3.16 × 10-1	1.126	0.047/0.059	3.03 × 10-1	0.789	6.76 × 10-1	1.045	46.73
rs79495679	*SOX5*	12:24501604	T/C	CPO	0.488/0.434	5.02 × 10-5	1.242	0.435/0.439	7.85 × 10-1	0.984	0.387/0.406	4.16 × 10-1	0.924	1.99 × 10-2	1.090	82.92
				CLO	0.447/0.434	3.32 × 10-1	1.053	0.435/0.439	7.87 × 10-1	0.984	0.426/0.405	3.38 × 10-1	1.094	3.79 × 10-1	1.033	0
				CLP				0.441/0.439	8.68 × 10-1	1.009	0.383/0.406	3.55 × 10-1	0.908	7.71 × 10-1	0.986	0
rs940224	*RORA*	15:61288213	A/G	CPO	0.388/0.288	7.39 × 10-6	1.565	0.433/0.423	4.91 × 10-1	1.043	0.424/0.385	9.72 × 10-2	1.175	7.05 × 10-5	1.280	92.06
				CLO	0.406/0.288	1.03 × 10-6	1.689	0.403/0.423	1.70 × 10-1	0.920	0.442/0.386	1.26 × 10-2	1.263	2.11 × 10-1	1.062	88.86
				CLP				0.414/0.423	5.29 × 10-1	0.966	0.433/0.385	5.45 × 10-2	1.220	7.20 × 10-1	1.018	74.83
rs9557809	*FGF14*	13:102779262	C/T	CPO	0.099/0.103	6.64 × 10-1	0.962	0.094/0.098	6.16 × 10-1	0.950	0.104/0.11	6.87 × 10-1	0.940	4.45 × 10-1	0.954	0
				CLO	0.077/0.103	9.72 × 10-4	0.727	0.093/0.098	5.36 × 10-1	0.939	0.116/0.11	6.59 × 10-1	1.066	1.94 × 10-2	0.863	66.17
				CLP				0.088/0.098	2.08 × 10-1	0.888	0.11/0.11	9.99 × 10-1	1.000	2.76 × 10-1	0.915	0
rs9601465	*SPRY2;LINC00377*	13:81408459	T/G	CPO	0.176/0.144	8.36 × 10-4	1.269	0.147/0.163	1.53 × 10-1	0.888	0.141/0.138	8.72 × 10-1	1.022	1.23 × 10-1	1.081	81.46
				CLO	0.156/0.144	2.03 × 10-1	1.098	0.16/0.163	7.95 × 10-1	0.979	0.124/0.138	3.69 × 10-1	0.885	7.05 × 10-1	1.019	14.65
				CLP				0.165/0.163	7.94 × 10-1	1.020	0.121/0.138	3.34 × 10-1	0.863	8.55 × 10-1	0.988	0
rs9866219	*LINCR-0002;FGF12*	3:191811437	A/C	CPO	0.148/0.135	1.58 × 10-1	1.113	0.154/0.151	7.29 × 10-1	1.029	0.115/0.119	8.26 × 10-1	0.968	2.66 × 10-1	1.060	0
				CLO	0.176/0.135	9.48 × 10-6	1.372	0.169/0.151	9.53 × 10-2	1.145	0.123/0.119	7.68 × 10-1	1.042	2.32 × 10-5	1.237	55.3
				CLP				0.163/0.151	2.16 × 10-1	1.098	0.113/0.119	7.12 × 10-1	0.943	3.37 × 10-1	1.068	0

(a) Most possible susceptibility or cause genes in this region; (b) MAF: Affected/Unaffected; (c) 930 CPO,945 CLO and 5068 Controls; (d) 724 CPO, 781 CLO, 2270 CLP and 3265 Controls,(e) 417 CPO,492 CLO, 427 CLP and 1832 Controls; (f) totally 17151 samples: 2071 CPO, 2218 CLO, 2697 CLP and 10165 Controls; (g) I^2 heterogeneity. CHS: Southern Chinese; CHN: Northern Chinese.
